# Impact of genotype and phenotype on cardiac biomarkers in patients with transthyretin amyloidosis – Report from the Transthyretin Amyloidosis Outcome Survey (THAOS)

**DOI:** 10.1371/journal.pone.0173086

**Published:** 2017-04-06

**Authors:** Arnt V. Kristen, Mathew S. Maurer, Claudio Rapezzi, Rajiv Mundayat, Ole B. Suhr, Thibaud Damy, on behalf of the THAOS investigators

**Affiliations:** 1 Amyloidosis Center, Department of Cardiology, Heidelberg University, Heidelberg, Germany; 2 Center for Advanced Cardiac Care, Columbia University Medical Center, New York, New York, United States of America; 3 Institute of Cardiology, University of Bologna and S. Orsola-Malpighi Hospital, Bologna, Italy; 4 Pfizer Inc., New York, New York, United States of America; 5 Department of Public Health and Clinical Medicine, Umeå University, Umeå, Sweden; 6 Amyloidosis Network, Department of Cardiology, CHU Henri Mondor, Creteil, France; University of California, Davis, UNITED STATES

## Abstract

**Aim:**

Cardiac troponins and natriuretic peptides are established for risk stratification in light-chain amyloidosis. Data on cardiac biomarkers in transthyretin amyloidosis (ATTR) are lacking.

**Methods and results:**

Patients (n = 1617) with any of the following cardiac biomarkers, BNP (n = 1079), NT-proBNP (n = 550), troponin T (n = 274), and troponin I (n = 108), available at baseline in the Transthyretin Amyloidosis Outcomes Survey (THAOS) were analyzed for differences between genotypes and phenotypes and their association with survival. Median level of BNP was 68.0 pg/mL (IQR 30.5–194.9), NT-proBNP 337.9 pg/mL (IQR 73.0–2584.0), troponin T 0.03 μg/L (IQR 0.01–0.05), and troponin I 0.08 μg/L (IQR 0.04–0.13). NT-proBNP and BNP were higher in wild-type than mutant-type ATTR, troponin T and I did not differ, respectively. Non-Val30Met patients had higher BNP, NT-proBNP and troponin T levels than Val30Met patients, but not troponin I. Late-onset Val30Met was associated with higher levels of troponin I and troponin T compared with early-onset. 115 patients died during a median follow-up of 1.2 years. Mortality increased with increasing quartiles (BNP/NT-proBNP Q1 = 1.7%, Q2 = 5.2%, Q3 = 21.7%, Q4 = 71.3%; troponin T/I Q1 = 6.5%, Q2 = 14.5%, Q3 = 33.9%, Q4 = 45.2%). Three-year overall-survival estimates for BNP/NT-proBNP and troponin T/I quartiles differed significantly (*p*<0.001). Stepwise risk stratification was achieved by combining NT-proBNP/BNP and troponin T/I. From Cox proportional hazards model, age, modified body mass index, mutation (Val30Met vs. Non-Val30Met) and BNP/NT-proBNP (Q1–Q3 pooled vs. Q4) were identified as independent predictors of survival in patients with mutant-type ATTR.

**Conclusions:**

In this ATTR patient cohort, cardiac biomarkers were abnormal in a substantial percentage of patients irrespective of genotype. Along with age, mBMI, and mutation (Val30Met vs. Non-Val30Met), cardiac biomarkers were associated with surrogates of disease severity with BNP/NT-proBNP identified as an independent predictor of survival in ATTR.

**Trial registration:**

ClinicalTrials.gov NCT00628745

## Introduction

Transthyretin (TTR) amyloidosis (ATTR) is the most common form of hereditary systemic amyloid disease with a broad spectrum of genotypes and heterogeneous phenotypes. TTR is a tetramer that binds thyroxine and retinol binding protein. Dissociation of the tetramer into monomers is regarded as the initial step into amyloid formation, and TTR mutations and advanced age increases the instability of the tetramer and facilitates its dissociation into monomers that, following conformational changes, reassembles into amyloid fibrils [1].

More than 100 amyloidogenic TTR point mutants are described, of which the TTR Val30Met preferentially gives rise to a sensorimotor amyloidotic polyneuropathy (TTR-FAP) [2], whereas other mutations, such as the Val122Ile, predispose to the development of cardiomyopathy (TTR-FAC) [3–8]. Age of disease onset and clinical presentation depend on endemic area, gender, and the underlying genotype. Moreover, wild-type (WT) TTR can assemble into amyloid fibrils, leading predominantly to an amyloid cardiomyopathy: senile systemic amyloidosis. This age- and gender-related amyloidosis is found in a large number of predominantly male patients, older than 75 years, with heart failure and preserved ejection fraction [9]. Despite the similar histological and morphological presentation of cardiac amyloid, median survival of patients with ATTR is superior to that of individuals with light-chain amyloidosis (AL) [10]. At present, clinical variables that may be useful for assessment of disease severity and prognosis in patients with ATTR are not elucidated in detail [11,12]. In patients with AL, plasma levels of cardiac troponin T and N-terminal pro-brain natriuretic peptide (NT-proBNP) provide potent clinical information [9,13–15]. Cardiac troponin T is a highly specific and sensitive marker of myocardial injury [16,17], while NT-proBNP may be considered a sensitive indicator of cardiac overload. Data on cardiac biomarkers in different types of ATTR are lacking.

In the Transthyretin Amyloidosis Outcomes Survey (THAOS), data on cardiac biomarkers troponin T, troponin I, NT-proBNP, and BNP from a large and heterogeneous cohort of patients with different types of ATTR are recorded. 2535 patients have been entered in the study as of 06 January 2015, providing a unique opportunity to identify the impact of genotypes and phenotypes on cardiac biomarkers and their association with survival.

## Methods

THAOS is an ongoing international, longitudinal, observational registry that collects data on the natural history of ATTR (ClinicalTrials.gov: NCT00628745; http://www.clinicaltrials.gov/ct2/show/NCT00628745), open to all patients with hereditary or WT ATTR and asymptomatic TTR-variant carriers [18]. Prior to enrolling patients in THAOS, participating sites obtained approval from their local Ethical Review Board/Institutional Review Board and satisfied all national ethical and regulatory requirements. All patients were more than 18 years of age and provided written informed consent. Confidentiality is maintained according to applicable regulations and guidelines. Research is in accordance with the Declaration of Helsinki. A Scientific Board comprising participating physicians, clinical experts, and a medical representative of the sponsor gave advice on scientific and policy decisions and oversaw data review and analysis.

### Data collection

Data obtained during routine clinical practice are entered into THAOS at each visit using a secure internet-based application. Information entered into THAOS is de-identified.

Of the cardiac biomarkers, measurement of NT-proBNP or BNP is encouraged in all patients. However, the individual physicians decide the examinations performed on their patients according to their standard of care. Tests are recommended to be performed using standardized methods as endorsed by national or international societies. To minimize data entry errors, units for each parameter are clearly specified and there is a user alert if a value entered falls outside a pre-specified range.

### Data extraction and analysis

Data extraction was performed on January 6, 2015 when 2535 patients were entered into the THAOS registry. Five patients had polymorphisms and were excluded from the analysis. This study reports on a subgroup of 1617 patients (63.8% of the overall THAOS population) with data on cardiac biomarkers available at baseline. Data on BNP were available in 1079 patients (42.6% of the overall THAOS population) and on NT-proBNP in 550 patients (21.7% of the overall THAOS population); both BNP and NT-proBNP data were available for eight patients. Data on cardiac biomarkers were reported from 21 of 36 THAOS sites.

Of the 1617 patients with data on natriuretic peptides, troponin T was available in 274 patients (10.8% of the overall THAOS population) and troponin I in 108 patients (4.3% of the overall THAOS population); both troponin T and troponin I data were available for two patients. Demographics and levels of cardiac biomarkers were compared among different types of ATTR: WT vs. mutant (MT) ATTR, Val30Met vs. non-Val30Met mutations, and symptomatic Val30Met patients with early vs. late onset of disease, respectively. Since each biomarker was obtained for clinical indications at respective sites (*n* = 21) in different laboratories with different reference values, each biomarker was divided into four quartiles (Q1≤25%; 25%<Q2≤50%; 50%<Q3≤75%; 75%<Q4≤100%), respectively. These individual quartiles were subsequently pooled in a combined quartile of natriuretic peptides or troponin. BNP/NT-proBNP quartile assignment is based on the quartile values of the available measure. In the case of patients with both BNP and NT-proBNP available, BNP was used. Troponin T/I quartile assignment is based on the quartile values of the available measure. In the case of patients with both troponin I and T available, troponin I was used. Cardiac biomarkers were defined as normal if NT-proBNP <1000 pg/mL, BNP <400 pg/mL, troponin T <0.01 μg/L, and troponin I <0.01 μg/L. Moreover, optimal cut point for cardiac biomarkers were identified by the approach of Contal and O’Quigley [19].

Clinical data were compared after dividing patients into mutant and non-mutant type. Mutant patients were separated according to TTR mutation (Val30Met vs. non-Val30Met). Finally, patients with Val30Met mutation were divided according to early (<50 years of age) vs. late (≥50 years of age) onset of disease.

### Statistical analysis

Continuous data were expressed as either mean and standard deviation (SD) or median and interquartile range depending on the distribution. Categorical variables were expressed as absolute numbers and percentages. Comparisons between cohorts and subgroups were carried out using the one-way analysis of variance (ANOVA) and Kruskal–Wallis test as appropriate; Chi-square test was used for categorical variables. Bonferroni correction was applied for comparisons of demographic and baseline characteristics. Pearson’s correlation coefficients were calculated to examine the association between BNP/NT-proBNP, Troponin I/T, and clinical echocardiographic parameters. Overall survival was defined as the time between measurement of biomarkers and death from any cause irrespective of heart/liver transplantation. Differences in overall survival by levels of BNP/NT-proBNP and troponin I/T (i.e., Q1–Q3 pooled vs. Q4 as well as optimal cut-off points by the approach of Contal and Quigley [19]) were assessed respectively, using log-rank analysis with right-censoring. The last available follow-up was used as a cut-off date for censoring patients who did not experience event. Curves were displayed by the Kaplan–Meier product limit method. Cox proportional hazards analysis was carried out to identify potential risk factors of survival. Statistically significant predictors of survival from the univariate analysis were then included in a multivariate Cox proportional hazards model. A stepwise selection method was utilized for the entry and retention of variables in the model. *p*<0.05 was considered statistically significant. SAS 9.1.3 (Cary, NC, USA) was used for all analyses.

## Results

The present study cohort of 1617 patients with TTR-related amyloidosis consisted of 1452 patients with MT [728 females, 50.1%; median age 41.5 (range 18.3–86.2) years] and 165 patients with WT [8 females, 4.8%; median age 75.3 (range 48.0–89.6) years]. These patients did not differ from the cohort of patients in THAOS without data on cardiac biomarkers available except for age, age at onset of ATTR symptoms, prevalence of cardiac symptoms, mBMI, Karnofsky index, and renal function (Table 1). In total, 952 of 1452 (65.6%) MT patients and 86 of 165 (52.1%) WT patients presented with neurologic symptoms of ATTR. 355 of 1452 (24.4%) MT patients and 152 of 165 (92.1%) WT patients presented with cardiac symptoms of ATTR. The median age for onset of symptoms was 38.0 (range 10–82) years in MT and 68.8 (range 33–89) years in WT patients. The most common TTR mutation was Val30Met in 1210 patients (83.3% of MT patients). The median BNP plasma level was 68.0 pg/mL [interquartile range (IQR) 30.5, 194.9] [*n* = 1079, 169(16%) with abnormal values], median NT-proBNP plasma level was 337.9 pg/mL (IQR 73.0, 2584.0) [*n* = 550, 220(40%) with abnormal values]. Median troponin T plasma level was 0.03 μg/L (IQR 0.01, 0.05) [*n* = 274, 163 (59%) with abnormal values], median troponin I plasma level was 0.08 μg/L (IQR 0.0, 1.0) [*n* = 108, 101 (94%) with abnormal values]. In asymptomatic patients, BNP or NT-proBNP values were above the upper reference limit in 43 (13.1%) and 18 (16.5%) patients, respectively, whereas troponin T or I values were above the upper reference limit in 7 (15.6%) and 13 (81.3%) patients, respectively.

**Table 1:** Demographics of symptomatic patients.

	Without Biomarkers (n = 913)	With Biomarkers (n = 1617)	*P*-Value^a^	Wild Type (n = 165)	Mutant Type (n = 1452)	Val30Met (n = 1210)	Non-Val30Met (n = 242)	*P*-Value^b^	Early Onset (n = 615)	Late Onset (n = 213)	*P*-Value^c^
**Female**	379 (41.5%)	736 (45.5%)	0.0513	8 (4.8%)	728 (50.1%)	646 (53.4%)	82 (33.9%)	<0.0001	312 (50.7%)	94 (44.1%)	0.0968
**Age (years)**	51.0 [36.8, 67.5]	43.9 [33.0, 64.4]	<0.0001	75.3 [71.3, 80.4]	41.5 [31.9, 57.7]	37.9 [30.4, 50.9]	61.4 [52.9, 69.0]	<0.0001	37.0 [31.6, 43.8]	66.9 [60.6, 71.9]	<0.0001
**Age at onset of symptoms, (years)**	46.5 [33.1, 61.5]	41.7 [31.0, 59.4]	0.0005	68.8 [61.8, 75.5]	38.0 [29.8, 54.5]	34.8 [28.8, 48.8]	55.5 [46.1, 64.3]	<0.0001	32.2 [27.4, 37.4]	59.8 [55.0, 65.1]	<0.0001
**Cardiac symptoms**	354 (38.8%)	507 (31.4%)	0.0002	152 (92.1%)	355 (24.4%)	215 (17.8%)	140 (57.9%)	<0.0001	126 (20.5%)	89 (41.8%)	<0.0001
**Neurological symptoms**	601 (65.8%)	1038 (64.2%)	0.4086	86 (52.1%)	952 (65.6%)	795 (65.7%)	157 (64.9%)	0.8049	595 (96.7%)	200 (93.9%)	0.0668
**mBMI (kg/m**^**2**^**·g/L)**	996 ± 262	1078 ± 236	<0.0001	1080 ± 191	1077 ± 240	1077 ± 235	1079 ± 270	0.9505	1036 ± 232	1091 ± 269	0.0073
	(n = 202)	(n = 1419)		(n = 116)	(n = 1303)	(n = 1140)	(n = 163)		(n = 584)	(n = 185)	
**Karnofsky index (%)**	90 [70, 100]	90 [80, 100]	<0.0001	80 [70, 90]	90 [80, 100]	90 [80, 100]	80 [70, 90]	<0.0001	90 [80, 90]	80 [70, 90]	<0.0001
	(n = 677)	(n = 1451)		(n = 106)	(n = 1345)	(n = 1160)	(n = 185)		(n = 592)	(n = 190)	
**eGFR (mL/min/1.73 m**^**2**^**)**	87.4 ± 42.3	104.5 ± 120.4	0.0165	55.7 ± 21.1	110.0 ± 125.5	112.9 ± 72.0	94.4 ± 268.9	0.0444	109.8 ± 38.5	81.7 ± 30.1	<0.0001
	(n = 292)	(n = 1565)		(n = 157)	(n = 1408)	(n = 1186)	(n = 222)		(n = 606)	(n = 198)	
**History of liver transplant**	157 (17.2%)	249 (15.4%)	0.2369	0 (0%)	249 (17.1%)	228 (18.8%)	21 (8.7%)	0.0001	201 (32.7%)	22 (10.3%)	<0.0001

Data are absolute numbers (percentage), mean ± standard deviation, or median [interquartile range]. Cardiac symptoms are defined as coronary artery disease, dyspnoea, heart failure, myocardial infarction, rhythm disturbances, and syncope. Neurologic symptoms include balance abnormalities, muscle weakness, neuropathic pain/paresthesia, numbness, temperature or pain insensitivity, tingling, walking disability.

ATTR, transthyretin- amyloidosis; eGFR, estimated glomerular filtration rate; mBMI, modified body mass index.

^a^*P*-values are With vs. Without Biomarker data

^b^*P*-values are Val30Met vs. non-Val30Met.

^c^*P*-values are early onset vs. late onset.

The bonferroni corrected p-value adjusting for nine comparisons is 0.005556.

### Analysis according to type of ATTR

**Fig 1:**
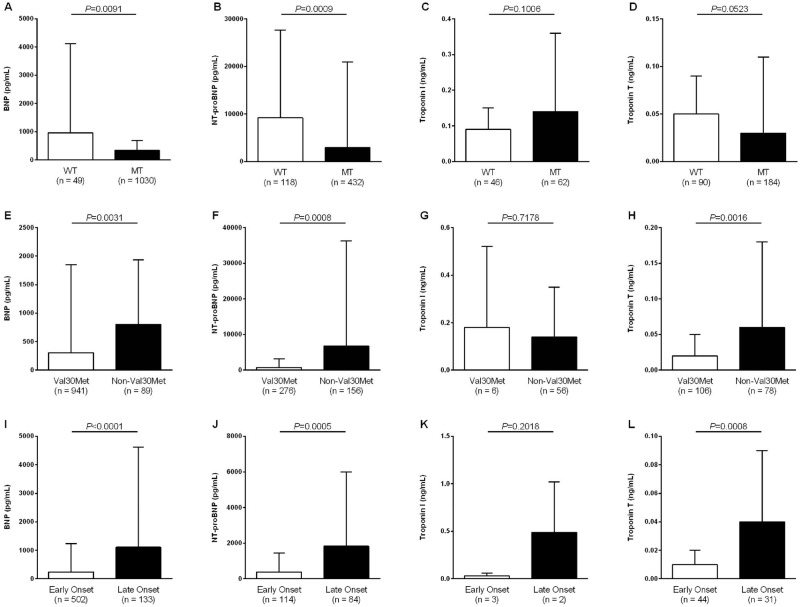
Unadjusted comparison of cardiac biomarkers in patients with ATTR by genotype, mutation, and age at disease onset. (A) BNP in patients with wild type (WT) vs. mutant type (MT); (B) NT-proBNP in patients with WT vs. MT; (C) Troponin I in patients with WT vs. MT; (D) Troponin T in patients with WT vs. MT; (E) BNP in patients with Val30Met vs. non-Val30Met mutations; (F) NT-proBNP in patients with Val30Met vs. non-Val30Met mutations; (G) Troponin I in patients with Val30Met vs. non-Val30Met mutations; (H) Troponin T in patients with Val30Met vs. non-Val30Met mutations; (I) BNP in patients with early vs. late onset of ATTR due to Val30Met mutation; (J) NT-proBNP in patients with early vs. late onset of ATTR due to Val30Met mutation; (K) Troponin I in patients with early vs. late onset of ATTR due to Val30Met mutation; (L) Troponin T in patients with early vs. late onset of ATTR due to Val30Met mutation.

Demographics according to ATTR type and mutation are shown in Table 1. NT-proBNP and BNP plasma levels of unadjusted analysis in WT vs. MT patients, Val30Met vs. non-Val30Met mutations, as well as late onset of Val30Met ATTR vs. early onset of disease were presented in Fig 1, respectively. Comparison of troponin T and troponin I plasma levels is demonstrated in Fig 1. However, number of patients with troponin I was limited in the group of Val30Met patients (n = 6). Thus, comparison of troponin I between early and late onset was excluded.

### Analysis according to level of BNP and NT-proBNP

Detailed demographic data according to the combined quartiles of natriuretic peptides are shown in Table 2. Age and age at onset of symptoms increased significantly from Q1 to Q4. A higher prevalence of non-Val30Met patients was observed in Q4 as compared to Q1, Q2, and Q3, respectively. In contrast, percentage of patients with Val30Met mutation was similar in Q1 and Q2 and decreased in Q3 to Q4. Moreover, patients with higher levels of BNP/NT-proBNP presented with poorer condition as indicated by decrease of Karnofsky index, modified body mass index (mBMI), and renal function from Q1 to Q4, respectively. Prevalence of neurological symptoms and liver transplant was higher in Q3 and Q4 as compared with Q1 and Q2. Optimal cut-off point was 145 pg/mL for BNP (p < .001) and 325 pg/mL for NT-proBNP (p < .001).

**Table 2:** Demographics of patients according to the combined quartiles of cardiac biomarkers.

	Combined Quartiles of Natriuretic Peptides^†^				Combined Quartiles of Troponin^‡^			
	Quartile 1	Quartile 2	Quartile 3	Quartile 4	Quartile 1	Quartile 2	Quartile 3	Quartile 4
**Cut-off value of biomarkers**								
	BNP ≤30.5	BNP ≤68.0	BNP ≤194.9	BNP >194.9	Troponin I ≤0.04	Troponin I ≤0.08	Troponin I ≤0.12	Troponin I >0.12
	NT-proBNP ≤73.0	NT-proBNP ≤337.9	NT-proBNP ≤2584.0	NT-proBNP >2584.0	Troponin T ≤0.01	Troponin T ≤0.03	Troponin T ≤0.05	Troponin T >0.05
	(Unit: pg/mL)	(Unit: pg/mL)	(Unit: pg/mL)	(Unit: pg/mL)	(Unit: μg/L)	(Unit: μg/L)	(Unit: μg/L)	(Unit: μg/L)
**N**	407	404	402	404	138	48	94	87
**Female, n (%)**	151 (37.1%)	247 (61.1%)	199(49.5%)	139 (34.4%)	64 (46.4%)	9 (18.8%)	17 (18.1%)	8 (9.2%)
**Age (years)**	34.4 [27.3, 44.8]	37.0 [30.8, 48.1]	47.9 [34.9, 67.8]	66.9 [54.4, 74.7]	48.6 [35.7, 64.0]	68.3 [60.0, 77.2]	71.1 [64.2, 76.4]	72.6 [67.1, 77.5]
**Symptoms of ATTR**	255 (62.7%)	316 (78.2%)	352 (87.6%)	386 (95.5%)	101 (69.0%)	45 (83.8%)	89 (85.7%)	83 (87.5%)
**Age at onset of symptoms (years)**	33.5 [27.4, 42.7]	34.5 [29.8, 46.1]	44.9 [30.5, 61.5]	57.5 [43.1, 68.2]	48.1 [35.5, 60.5]	63.2 [49.9, 69.7]	64.3 [54.5, 70.5]	66.0 [57.3, 71.3]
**Genotype (% according to genotype)**								
WT	2 (0.5%)	0 (0.0%)	50 (12.4%)	113 (28.0%)	16 (11.6%)	19 (39.6%)	47 (50.0%)	43 (49.4%)
Val30Met	366 (89.9%)	368 (91.1%)	290 (72.1%)	186 (46.0%)	79 (57.2%)	12 (25.0%)	11 (11.7%)	9 (10.3%)
Early onset^‡^	159(87.4%)	191 (79.9%)	162 (70.4%)	103 (58.2%)	35 (71.4%)	7 (63.6%)	5 (50.0%)	0
Late onset^‡^	23 (12.6%)	48 (20.1%)	68 (29.6%)	74 (41.8%)	14 (28.6%)	4 (36.4%)	5 (50.0%)	9 (100.0%)
Non-Val30Met	39 (9.6%)	36 (8.9%)	62 (15.4%)	105 (26.0%)	43 (31.2%)	17 (35.4%)	36 (38.3%)	35 (40.2%)
**Cardiac symptoms**	24 (5.9%)	44 (10.9%)	145 (36.1%)	294 (72.8%)	39 (28.3%)	29 (60.4%)	79 (84.0%)	73 (83.9%)
**Cardiac biomarkers**								
BNP (pg/mL)	17.6 ± 7.3	47.1 ± 10.7	115.8 ± 36.5	1329.7 ± 3073.2	148.1 ± 165.9	501.7 ± 443.9	994.0 ± 1158.6	1773.7 ± 4311.6
	(n = 270)	(n = 271)	(n = 269)	(n = 269)	(n = 65)	(n = 32)	(n = 13)	(n = 26)
NT-proBNP (pg/mL)	38.9 ± 16.8	166.4 ± 82.7	1342.3 ± 692.6	15,332.4 ± 34,151.9	1309.7 ± 5048.6	4718.3 ± 11,698.2	6253.5 ± 11,685.4	17,401.6 ± 47,378.8
	(n = 139)	(n = 136)	(n = 136)	(n = 139)	(n = 75)	(n = 17)	(n = 82)	(n = 62)
Troponin I (μg/L)	0.18 ± 0.36	0.02 ± 0.01	0.06 ± 0.05	0.14 ± 0.17	0.02 ± 0.01	0.05 ± 0.01	0.12 ± 0.09	0.28 ± 0.26
	(n = 7)	(n = 6)	(n = 21)	(n = 74)	(n = 29)	(n = 23)	(n = 27)	(n = 29)
Troponin T (μg/L)	0.03 ± 0.14	0.02 ± 0.03	0.03 ± 0.03	0.06 ± 0.05	0.01 ± 0.00	0.02 ± 0.00	0.04 ± 0.01	0.11 ± 0.12
	(n = 47)	(n = 39)	(n = 80)	(n = 108)	(n = 111)	(n = 26)	(n = 73)	(n = 64)
**Neurological symptoms**	192 (47.2%)	246 (60.9%)	293 (72.9%)	307 (76.0%)	78 (56.5%)	34 (70.8%)	61 (64.9%)	48 (55.2%)
**Motor neuropathy**	45 (11.1%)	84 (20.8%)	117 (29.1%)	155 (38.4%)	–	–	–	–
**Sensory motorneuropathy**	164 (40.3%)	223 (55.2%)	252 (62.7%)	265 (65.6%)	–	–	–	–
**Autonomic neuropathy**	135 (33.2%)	195 (48.3%)	241 (60.0%)	246 (60.9%)	–	–	–	–
**mBMI (kg/m**^**2**^**·g/L)**	1180.4 ± 234.7	1086.4 ± 207.0	1035.5 ± 228.8	995.1 ± 234.5	1097.9 ± 241.7	1036.7 ± 195.1	1044.2 ± 201.7	1040.4 ± 204.0
	(n = 371)	(n = 377)	(n = 347)	(n = 324)	(n = 108)	(n = 37)	(n = 73)	(n = 63)
**Karnofsky index (%)**	100 [90, 100]	90 [80, 100]	90 [80, 90]	80 [70, 80]	90 [80, 100]	80 [80, 90]	80 [70, 90]	80 [70, 80]
	(n = 384)	(n = 387)	(n = 349)	(n = 331)	(n = 116)	(n = 30)	(n = 80)	(n = 59)
**eGFR (mL/min/1.73 m**^**2**^**)**	126.3 ± 73.0	114.6 ± 35.9	110.8 ± 221.3	66.5 ± 35.3	130.5 ± 339.3	77.4 ± 27.1	63.9 ± 24.9	50.0 ± 20.5
	(n = 395)	(n = 393)	(n = 384)	(n = 393)	(n = 136)	(n = 46)	(n = 88)	(n = 86)
**History of liver transplant**	35 (8.6%)	65 (16.1%)	79 (19.7%)	70 (17.3%)	26 (18.8%)	3 (6.3%)	6 (6.4%)	1 (1.1%)

Data are absolute numbers (percentage) or mean ± standard deviation, or median [interquartile range]. Definition of cardiac and neurologic symptoms see footnote of Table 1.

ATTR, transthyretin-associated amyloidosis; eGFR, estimated glomerular filtration rate; mBMI, modified body mass index; (NT-pro)BNP, (n-terminal pro) brain-type natriuretic peptide; WT, wild-type.

^†^BNP/NT-BNP quartile assignment is based on the quartile values of the available measure. In the case of subjects with both BNP and NT-BNP available, BNP quartile was used.

^‡^Troponin I/T quartile assignment is based on the quartile values of the available measure. In the case of subjects with both Troponin I and T available, Troponin T quartile was used.

^‡^ among symptomatic subjects

### Analysis according to level of troponin I and troponin T

Demographics according to the combined quartiles of troponin are shown in Table 2. Age and age at onset of symptoms increased significantly from Q1 to Q4. Prevalence of non-Val30Met patients was relatively unchanged from Q1 to Q4. The percentage of patients with Val30Met mutation decreased from Q1 to Q4. Patients with higher levels of troponin T/I presented with poorer condition as indicated by decrease of Karnofsky index, mBMI, and renal function from Q1 to Q4, respectively. Prevalence of liver transplant decreased from Q1 to Q4. Results of correlation of cardiac biomarkers with clinical data are shown in detail in Table 3. Optimal cut-off point was 0.063 μg/L for troponin I (p < .01) and 0.021 μg/L for troponin T (p < .001).

**Table 3:** Correlations of biomarkers with clinical and echocardiographic parameters.

	n	r	*P*-Value
**Correlation of log-transformed troponin I with**			
Age	107	0.222	0.021
Septal thickness	52	0.348	0.011
Left ventricular posterior wall thickness	52	0.434	0.001
Duration of disease	92	−0.258	0.013
**Correlation of log-transformed troponin T with**			
Age	274	0.656	<0.001
Left atrial diameter	190	0.452	<0.001
Septal thickness	202	0.556	<0.001
Left ventricular posterior wall thickness	201	0.606	<0.001
**Correlation of log-transformed BNP with**			
Age	1079	0.585	<0.001
mBMI	987	−0.319	<0.001
Left atrial diameter	148	0.571	<0.001
Septal thickness	175	0.605	<0.001
Left ventricular posterior wall	168	0.563	<0.001
Duration of disease	766	0.308	<0.001
**Correlation of log-transformed NT-proBNP with**			
Age	550	0.698	<0.001
mBMI	438	–0.236	<0.001
Left atrial diameter	267	0.337	<0.001
Septal thickness	307	0.654	<0.001
Left ventricular posterior wall thickness	302	0.649	<0.001
Duration of disease	457	0.186	<0.001

mBMI, modified body mass index; (NT-pro)BNP, (n-terminal pro) brain-type natriuretic peptide.

### Survival analysis

During a median follow-up of 1.2 years, 115 patients (7.1%) have died in THAOS due to any cause. Two patients (1.7%) died in the BNP/NT-proBNP quartile Q1 and 6 (5.2%) in Q2, respectively. There were 25 (21.7%) and 82 (71.3%) fatal events in the BNP/NT-proBNP quartiles Q3 and Q4, respectively. In the cohort of patients with troponin measurements, there were 4 (6.5%), 9 (14.5%), 21 (33.9%), and 28 (45.2%) deaths in Q1, Q2, Q3, and Q4, respectively. Three-year overall survival estimate for the patients below the optimal cut-point for BNP/NT-proBNP was 98.1% ± 0.6% and was 70.1% ± 3.1% for patients above the optimal cut-point, with significant difference between the two survival curves (p<0.001). Three-year overall survival estimate for the three BNP/NT-proBNP quartiles Q1 to Q3 combined was 95.8% ± 0.8% and of Q4 was 65.2% ± 3.9%, with significant difference between the two survival curves (p<0.001). Three-year overall survival estimate for the patients below the optimal cut-point for troponin T/I was 88.6% ± 5.0% and was 43.6% ± 6.0% for patients above the optimal cut-point, with significant difference between the two survival curves (p<0.001). Three-year overall survival estimates for troponin T/I quartiles Q1 to Q3 combined was 73.7% ± 4.8% and of Q4 was 32.5% ± 8.0%. There was a significant difference between the two survival curves (p<0.001). By combination of both biomarkers, stepwise risk stratification was achieved (Stage A: both natriuretic peptides AND troponins above; Stage B: both natriuretic peptides AND troponins below; Stage C: either of natriuretic peptides OR troponins is above.

**Fig 2:**
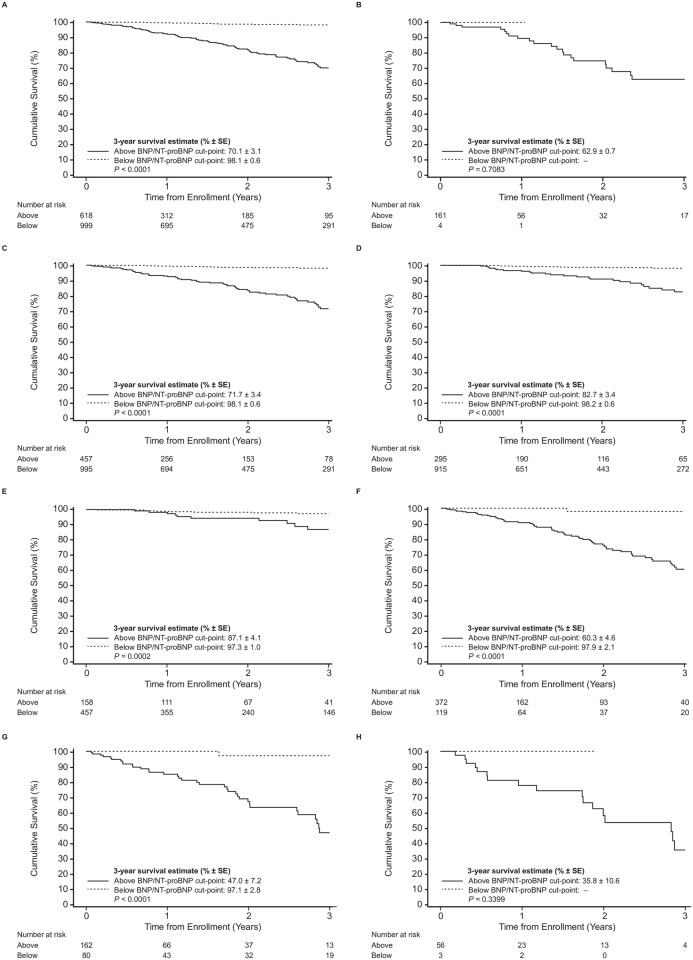
Association of survival and plasma levels of natriuretic peptides in patients with ATTR using unadjusted log-rank test. Survival stratified by natriuretic peptides (BNP/NT-proBNP) optimal cut-off values (A) in the whole cohort; (B) in patients with wildtype ATTR; (C) in patients with mutant type ATTR; (D) in patients with Val30Met; (E) in patients with early onset of Val30Met; (F) in patients with late onset of Val30Met; (G) in patients with non-Val30Met; (H) in patients with Val122Ile. *P*-values are for the comparison between the combination of above vs. below the cut-off value.

**Fig 3:**
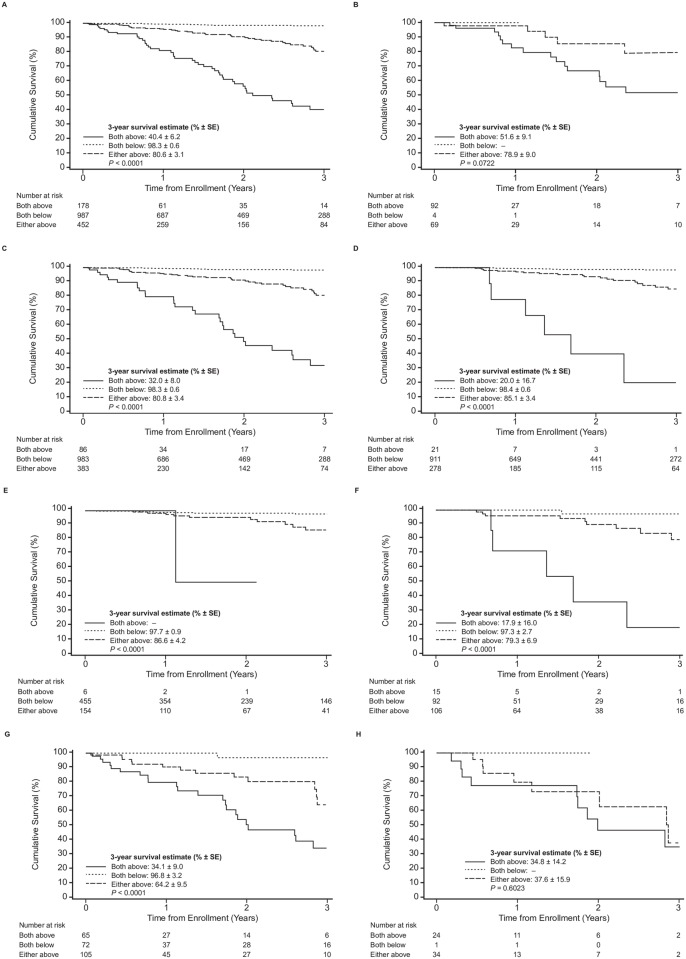
Association of survival and plasma levels of cardiac biomarkers in patients with ATTR using unadjusted log-rank test. Survival stratified by combination of troponin T/I cut-off values as well as NT-proBNP/BNP quartiles cut-off values (A) in the whole cohort; (B) in patients with wildtype ATTR; (C) in patients with mutant type ATTR; (D) in patients with Val30Met; (E) in patients with early onset of Val30Met; (F) in patients with late onset of Val30Met; (G) in patients with non-Val30Met; (H) in patients with Val122Ile. *P*-values are for the comparison between the combination of patients with values above vs. below the cut-off value. Stage A: both natriuretic peptides AND troponins above; Stage B both natriuretic peptides AND troponins below; Stage C: either of the natriuretic peptides OR troponins is above.

Kaplan-Meier curves were produced for patients grouped by optimal cut-off values for natriuretic peptides (Fig 2) and the combination of both biomarkers (Fig 3).

Results from the univariate analyses are presented in Table 4. Based on these results, age, gender, mBMI, duration of disease, estimated glomerular filtration rate (eGFR), and Val30Met/non-Val30Met were included in the multivariate Cox proportional hazards model. By multivariate analysis age [hazard ratio (HR) 1.062, 95% confidence interval (CI) 1.062–1.086], mutation (Val30Met vs. non-Val30Met, HR 0.367, 95% CI 0.199–0.676), mBMI (HR 0.998, 95% CI 0.996–0.999), and BNP/NT-proBNP (Q1–Q3 pooled vs. Q4, HR 0.508, 95% CI 0.278–0.928) were independent predictors of survival. Q4 of troponin did not make it to the final model due to too few non-missing observations.

**Table 4:** Potential risk factors of survival by univariate analysis.

Parameters	Model 1 (All)		Model 2 (TTR Mutation)	
	Hazard ratio (95% CI)	*P*-Value	Hazard ratio (95% CI)	*P*-Value
Age (per year)	1.08 (1.07,1.10)	<0.001	1.10 (1.08,1.12)	<0.001
Male (vs. female)	3.48 (2.24,5.39)	<0.001	2.75 (1.72,4.40)	<0.001
Liver transplant at any time (vs. no liver transplant)	0.99 (0.61,1.61)	0.97	1.36 (0.82,2.25)	0.24
Liver transplant prior to consent (vs. no liver transplant)	0.67 (0.32,1.37)	0.27	0.86 (0.42,1.79)	0.69
mBMI [per (kg/m^2^·g/L)]	1.00 (1.00–1.00)	<0.001	1.00 (1.00–1.00)	<0.001
Duration of disease (per year)	1.04 (1.02,1.06)	<0.001	1.04 (1.01,1.07)	0.002
eGFR [per (mL/min/1.73 m^2^)]	0.98 (0.97–0.98)	<0.001	0.98 (0.97–0.98)	<0.001
Val30Met (vs. non-Val30Met)			0.13 (0.09,0.21)	<0.001
Early Onset (vs. Late onset) ^‡^			0.185 (0.10,0.34)	<0.001
Val122i (vs Non-Val122i) ^‡‡^			2.99 (1.60,5.61)	<0.001

CI, confidence interval; mBMI modified body mass index; eGFR estimated glomerular filtration rate.

^‡^ among symptomatic subjects with Val30met mutation

^‡‡^ among Non-Val30Met subjects

## Discussion

This study comprised a large cohort of patients with different forms of ATTR with cardiac biomarkers available in the THAOS registry. Cardiac biomarkers were compared between different genotypes and phenotypes. Moreover, the predictive value for survival of ATTR was analyzed.

Among patients with the same subtype, e.g., ATTR, a wide heterogeneity of phenotypes was reported. For instance, in some patients with Val30Met, mutation symptoms of ATTR started at the age of 30 years (early onset disease) whereas others became symptomatic not until the fifth decade of life (late onset disease). Moreover, phenotype of early onset Val30Met is characterized by peripheral sensor-motoric polyneuropathy and conduction disturbances due to amyloid infiltration of the conductive system, whereas in other mutations, e.g., Val20Ile, Leu111Met, and Val122Ile, symptoms of heart failure predominate [20–22]. This heterogeneity may be explained by environmental factors and differences in amyloid fibril composition [23,24], and other factors previously noted by analysis of the large population of ATTR patients entered into THAOS, including age of onset, mutation, and inheritance pattern.

The present data indicated that higher plasma levels of cardiac biomarkers were observed in genotypes obviously associated with cardiac involvement, e.g., WT amyloidosis, non-Val30Met, and late-onset of Val30Met. Interestingly, increased biomarkers were observed in 45–90% of the patients with obviously neurologic phenotype, potentially indicating unidentified cardiac amyloidosis. Cardiac biomarkers were associated with increase of age and decrease of clinical condition (i.e., decrease of mBMI, Karnofsky index, and renal function) and, finally, higher plasma levels of troponin T/I or BNP/NT-proBNP were associated with poorer patient survival, with BNP/NT-proBNP as independent predictors of survival from the multivariate analysis.

### Cardiac biomarkers in ATTR amyloidosis

Several serological markers of heart muscle damage and heart failure have been established for diagnosis and risk stratification of different cardiac diseases. For instance, troponin T and I are sensitive markers for damage of cardiomyocytes. Both have been established as predictors of outcome in acute coronary syndrome [16,17]. Natriuretic peptides indicate cardiac overload and are widely used in clinical routine for management of heart failure [25].

Cardiac involvement is common in non-Val30Met TTR gene mutations, late-onset of Val30Met TTR gene mutation, and invariably present in WT patients. It may develop/progress in MT patients even after liver transplantation [12]. However, the use of cardiac biomarkers in patients with ATTR has not been elucidated in detail. In the present analysis, a marked variation of cardiac biomarkers was observed in diverse types of ATTR, underscoring the heterogeneity of the disease.

As expected, cardiac biomarkers were above the upper reference limit in a large number of patients with cardiac symptoms, with higher levels in variants associated with cardiac phenotype, e.g., non-Val30Met, late-onset of Val30Met, and WT. Interestingly, even among patients with supposed neurologic phenotype, elevation of cardiac biomarkers was observed. This might indicate cardiac damage due to (incidental) amyloid deposition in accordance with a previous report showing that natriuretic peptide is increased in ATTR with normal estimated left ventricle filling pressure [26]. Moreover, in a high percentage of patients without cardiac symptoms, levels of biomarkers above the upper limit of normal reference populations were observed (troponin I 81.3%, troponin T 15.6%, BNP 13.1%, NT-proBNP 16.5%). However, it needs to be taken into account that normal value thresholds of the individual biomarker have been reported in a healthy reference population. Defining abnormality of biomarker values is a critical step in its clinical use. Even if the value is within the defined reference limits, this may not necessarily indicate health in a given individual carrying a *TTR* gene variant. As inclusion of echocardiography data into THAOS was not mandatory and data were available for only a small number of patients, we are unable to draw any robust conclusion regarding severity of cardiac involvement, especially in these patients. In a recent report on the use of cardiac biomarkers (troponin T and I and BNP) in late onset Val30Met patients, BNP was demonstrated to be an early indicator of heart failure [27]. In these patients, abnormal values of BNP were already present in spite of normal basal strain values by echocardiography. Moreover, a correlation of BNP with several parameters of cardiac dysfunction and hypertrophy was demonstrated. NT-proBNP was not evaluated in this cohort. A significant correlation between increase of cardiac biomarkers and surrogate parameters was observed, which appears to be indicative of the severity of cardiac involvement. Thus, the measurement of cardiac biomarkers appears to be justified in all patients with ATTR. In cases of abnormal values, echocardiography or cardiac magnetic resonance imaging is recommended.

Troponin levels observed in the present study were considerably lower than those observed in light-chain amyloidosis. This may potentially indicate minor cardiac damage by ATTR amyloid, probably due to the absence of toxic effects of light-chains, as suggested in experimental models of light-chain amyloidosis [28]. In a Swedish cohort consisting of 29 patients who were evaluated for familial amyloid polyneuropathy, neither troponin T nor troponin I was a reliable marker for cardiac amyloidosis as detected by echocardiography [27]. In the present study, correlation of log-transformed values of natriuretic peptides was superior to correlation of raw troponin levels.

In general, in patients with chronic kidney disease, serum creatinine level affects troponin T, but has less impact on troponin I [29]. Moreover, impairment of renal function may be caused by interaction of cardiac and renal function (cardio-renal syndrome). Finally, renal involvement of ATTR, although rare [30], might explain the worsening of renal function. In total, 15 mutations of the TTR gene have so far been associated with renal involvement; among these Val30Met is by far the best characterized variant [31].

### Survival analyses

In the present analysis, the role of cardiac biomarkers in predicting outcome in patients with ATTR is presented for the first time. It was demonstrated that higher values of either natriuretic peptides (BNP >194.9 pg/mL, NT-proBNP >2584.0 pg/mL) or troponin T (>0.05 μg/L) and I (>0.58 μg/L) were predictive of survival in unadjusted analysis of patients with ATTR; however, only BNP/NT-proBNP (Q1–Q3 pooled vs. Q4) was identified as an independent predictor of survival in multivariate analysis. These results are well in line with previous results of light-chain amyloidosis. In light-chain amyloidosis, increased levels of troponin and NT-proBNP have been well established as independent predictors of poor outcome [9,15]. Troponin T elevation has been shown to correlate with a more aggressive disease and decreased survival [9,14] whereas NT-proBNP is helpful in assessing response to treatment [15]. Both NT-proBNP and troponins have been incorporated in a staging system widely used in clinical routine [13].

### Limitations

There are several limitations of investigations based on data collected in an observational survey. As data are collected during routine clinical practice from many centers/countries and at the discretion of the patient’s physician, there are inevitably variations in the type of clinical investigations conducted and in the completeness of data. Furthermore, such databases only capture data in pre-determined fields, which means that they are often slow to capture previously unsuspected manifestations/events. Databases are also subject to other biases, including variations in the expertise of participating physicians; a tendency for physicians to include the most affected individuals in the database or enter cardiac biomarker for patients with cardiac phenotype but not for those with neurologic phenotype; as well as the potential for certain genotypes to be over-represented. (% ± SE)

Biomarker measures are not available for all patients in the THAOS registry at baseline as this is not obligatory in this registry. Thus, this poses a strong risk of selection bias by the attending physicians, especially as both cohorts (i.e., patients with no information on cardiac biomarkers and those with biomarker measures) differ in age, prevalence of cardiac symptoms, mBMI, Karnofsky index, and renal function. At the end, the numbers of patients with troponin T and I are rather low, and availability of confounding factors, e.g., cardiovascular risk factors or pharmacotherapy of the patients, is limited. BNP/NT-proBNP was associated with survival in this cohort of patients with ATTR. Besides prediction of survival, troponins and natriuretic peptides might be helpful to identify cardiac involvement in patients with obviously sole neurological phenotype.

## Conclusions

In this large and heterogeneous cohort of patients with ATTR, cardiac biomarkers (natriuretic peptides and troponin) were abnormal in a substantial percentage of patients, irrespective of genotype and significantly associated with age and gender; they correlated with other measures of disease severity (mBMI, left ventricular posterior wall thickness, left atrial diameter) with BNP/NT-proBNP as an independent predictor of survival. Further studies are needed to evaluate whether increased levels of BNP/NT-proBNP reflect unidentified cardiac involvement as it is independent of the experience required from any investigator for detecting cardiac amyloidosis by echocardiography.

## Supporting information

S1 Supporting Information Source data. (ZIP)
