# Supplementary material for: Impact of genotype and phenotype on cardiac biomarkers in patients with transthyretin amyloidosis – Report from the Transthyretin Amyloidosis Outcome Survey (THAOS)
Source: PLoS One. 2017 Apr 6;12(4):e0173086. doi: 10.1371/journal.pone.0173086 (PMC5383030; doi:10.1371/journal.pone.0173086)
Supplement: S1 Supporting Information — (ZIP) [file pone.0173086.s001.zip › S1_Table_Q003_Table_2_v2.sas.rtf]

 Table 2. Comparison of Baseline Characteristics (Clinical, Biological, Echocardiography), Val30Met vs. Non-Val30Met*	

 	Overall
(N = 1452)	Val30Met
(N = 1210)	Non-Val30Met
(N = 242)	P-value
Val30Met vs. Non-Val30Met	
Gender, N (%)					
     Male	724 ( 49.9%)	564 ( 46.6%)	160 ( 66.1%)	<0.0001	
     Female	728 ( 50.1%)	646 ( 53.4%)	82 ( 33.9%)		
Age (yrs)					
     N	1452	1210	242	<0.0001	
     Mean ± SD	44.90 ±   16.23	41.91 ±   15.07	59.86 ±   13.29		
     Median	41.46	37.91	61.35		
     Min, Max	18.34,   86.23	18.34,   86.23	19.27,   85.29		
    25, 75 Percentile	31.90,   57.73	30.43,   50.93	52.93,   69.01		
Race/Ethnicity, N (%)					
     Caucasian	339 ( 23.3%)	182 ( 15.0%)	157 ( 64.9%)	<0.0001	
     African Descent	47 (  3.2%)	1 (  0.1%)	46 ( 19.0%)		
     Latino American	6 (  0.4%)	1 (  0.1%)	5 (  2.1%)		
     Asian	38 (  2.6%)	28 (  2.3%)	10 (  4.1%)		
     Other	10 (  0.7%)	4 (  0.3%)	6 (  2.5%)		
     Missing	1,012 ( 69.7%)	994 ( 82.1%)	18 (  7.4%)		
TTR genotype, N (%)					
     Val30Met	1,210 ( 83.3%)	1,210 (100.0%)	0 (  0.0%)	<0.0001	
     Non-Val30Met	242 ( 16.7%)	0 (  0.0%)	242 (100.0%)		
Age at onset of ATTR symptoms (yrs)					
     N	1151	937	214	<0.0001	
     Mean ± SD	42.06 ±   14.89	39.23 ±   13.80	54.47 ±   13.07		
     Median	37.99	34.77	55.48		
     Min, Max	9.72,   81.95	9.72,   81.95	17.79,   81.63		
    25, 75 Percentile	29.82,   54.50	28.77,   48.81	46.13,   64.26		
Age at measurement of BNP/NT-BNP (yrs)					
     N	1452	1210	242	<0.0001	
     Mean ± SD	44.91 ±   16.22	41.92 ±   15.07	59.86 ±   13.29		
     Median	41.46	38.01	61.23		
     Min, Max	18.34,   86.22	18.34,   86.22	19.27,   85.27		
    25, 75 Percentile	31.90,   57.72	30.42,   50.97	52.93,   69.00		
Age at measurement of Troponin I/T (yrs)					
     N	242	111	131	<0.0001	
     Mean ± SD	55.08 ±   16.27	47.42 ±   17.45	61.56 ±   11.87		
     Median	57.04	45.37	62.71		
     Min, Max	19.90,   85.27	19.98,   80.25	19.90,   85.27		
    25, 75 Percentile	42.42,   68.16	32.93,   63.68	55.25,   69.08		
Karnofsky index					
     N	1345	1160	185	<0.0001	
     Mean ± SD	87.23 ±   14.43	88.59 ±   13.07	78.65 ±   18.93		
     Median	90.00	90.00	80.00		
     Min, Max	0.00,  100.00	40.00,  100.00	0.00,  100.00		
    25, 75 Percentile	80.00,  100.00	80.00,  100.00	70.00,   90.00		
History of liver transplant, N (%)					
     No liver transplant	1,203 ( 82.9%)	982 ( 81.2%)	221 ( 91.3%)	0.0001	
     Liver transplant	249 ( 17.1%)	228 ( 18.8%)	21 (  8.7%)		
BNP (pg/mL)					
     N	1030	941	89	0.0031	
     Mean ± SD	348.41 ± 1516.55	305.53 ± 1541.34	801.84 ± 1135.20		
     Median	63.05	57.50	371.00		
     Min, Max	4.00,32434.00	4.00,32434.00	5.00, 5916.00		
    25, 75 Percentile	28.90,  168.20	27.00,  135.90	125.00,  947.80		
NT-BNP (pg/mL)					
     N	432	276	156	0.0008	
     Mean ± SD	2,912.03 ±18057.65	742.99 ± 2497.66	6,749.57 ±29537.13		
     Median	154.00	102.50	765.00		
     Min, Max	1.00,296450.0	13.00,25118.00	1.00,296450.0		
    25, 75 Percentile	56.00, 1149.00	49.00,  325.00	97.00, 3699.50		
Troponin I (ng/mL)					
     N	62	6	56	0.7178	
     Mean ± SD	0.14 ±    0.22	0.18 ±    0.34	0.14 ±    0.21		
     Median	0.07	0.04	0.07		
     Min, Max	0.00,    1.00	0.00,    0.86	0.01,    1.00		
    25, 75 Percentile	0.03,    0.13	0.00,    0.11	0.03,    0.14		
Troponin T (ng/mL)					
     N	184	106	78	0.0016	
     Mean ± SD	0.03 ±    0.08	0.02 ±    0.03	0.06 ±    0.12		
     Median	0.01	0.01	0.03		
     Min, Max	0.00,    1.00	0.00,    0.19	0.00,    1.00		
    25, 75 Percentile	0.01,    0.04	0.00,    0.01	0.01,    0.06		
Creatinine (mg/dL)					
     N	1414	1189	225	<0.0001	
     Mean ± SD	83.07 ±  165.14	74.42 ±   45.98	128.75 ±  397.89		
     Median	70.72	68.07	88.40		
     Min, Max	1.63, 6011.20	32.71,  981.24	1.63, 6011.20		
    25, 75 Percentile	59.23,   84.86	58.34,   80.44	70.72,  118.46		
Estimated GFR					
     N	1408	1186	222	0.0444	
     Mean ± SD	109.97 ±  125.54	112.87 ±   71.95	94.43 ±  268.86		
     Median	105.05	109.75	73.30		
     Min, Max	0.00, 4040.30	0.00, 1495.00	0.00, 4040.30		
    25, 75 Percentile	79.60,  128.05	86.30,  131.10	52.60,  103.00		
Modified BMI					
     N	1303	1140	163	0.9505	
     Mean ± SD	1,077.43 ±  239.87	1,077.27 ±  235.36	1,078.52 ±  270.08		
     Median	1054.06	1054.47	1049.27		
     Min, Max	413.82, 2094.79	413.82, 2022.07	530.02, 2094.79		
    25, 75 Percentile	918.27, 1208.33	923.41, 1205.79	883.79, 1233.65		
Left atrium (mm)					
     N	302	174	128	<0.0001	
     Mean ± SD	40.14 ±   11.15	37.80 ±   12.44	43.31 ±    8.13		
     Median	39.00	36.00	42.00		
     Min, Max	13.00,  170.00	13.00,  170.00	25.00,   64.00		
    25, 75 Percentile	34.00,   45.00	32.00,   42.00	39.00,   48.00		
LV septum (mm)					
     N	353	191	162	<0.0001	
     Mean ± SD	14.37 ±    5.05	13.01 ±    4.67	15.97 ±    5.03		
     Median	13.00	12.00	16.00		
     Min, Max	2.30,   29.00	6.00,   29.00	2.30,   27.00		
    25, 75 Percentile	10.00,   18.00	10.00,   16.00	12.00,   20.00		
LV posterior wall (mm)					
     N	339	180	159	<0.0001	
     Mean ± SD	12.45 ±    4.25	10.71 ±    3.42	14.43 ±    4.24		
     Median	12.00	10.00	14.00		
     Min, Max	2.20,   26.00	4.00,   26.00	2.20,   24.00		
    25, 75 Percentile	9.00,   16.00	8.00,   12.00	11.00,   18.00		
LV diastolic diameter (mm)					
     N	327	172	155	0.0895	
     Mean ± SD	45.26 ±    6.55	45.84 ±    5.58	44.61 ±    7.45		
     Median	45.00	46.00	45.00		
     Min, Max	3.60,   67.00	28.00,   67.00	3.60,   65.00		
    25, 75 Percentile	41.00,   50.00	42.00,   50.00	40.00,   50.00		
LV systolic diameter (mm)					
     N	293	155	138	<0.0001	
     Mean ± SD	29.89 ±    7.07	28.30 ±    5.27	31.68 ±    8.32		
     Median	29.00	28.00	31.15		
     Min, Max	2.50,   55.00	2.50,   41.00	3.00,   55.00		
    25, 75 Percentile	25.00,   34.00	25.00,   31.00	27.00,   35.00		
End diastolic volume (mL)					
     N	2	0	2		
     Mean ± SD	88.50 ±   60.10		88.50 ±   60.10		
     Median	88.50		88.50		
     Min, Max	46.00,  131.00		46.00,  131.00		
    25, 75 Percentile	46.00,  131.00		46.00,  131.00		
End systolic volume (mL)					
     N	1	0	1		
     Mean ± SD	24.00 ±     .		24.00 ±     .		
     Median	24.00		24.00		
     Min, Max	24.00,   24.00		24.00,   24.00		
    25, 75 Percentile	24.00,   24.00		24.00,   24.00		
Stroke volume index					
     N	149	105	44	<0.0001	
     Mean ± SD	71.00 ±   22.00	75.92 ±   19.40	59.25 ±   23.57		
     Median	70.00	75.00	56.50		
     Min, Max	23.00,  127.00	30.00,  127.00	23.00,  116.00		
    25, 75 Percentile	56.00,   87.00	63.00,   89.00	45.00,   68.50		
LV ejection fraction (%)					
     N	262	113	149	<0.0001	
     Mean ± SD	53.62 ±   14.03	60.14 ±    9.63	48.68 ±   14.83		
     Median	56.00	60.00	55.00		
     Min, Max	15.00,   83.00	30.00,   83.00	15.00,   80.00		
    25, 75 Percentile	45.00,   63.00	55.00,   66.00	37.00,   60.00		
E/A ratio					
     N	122	74	48	<0.0001	
     Mean ± SD	1.47 ±    0.86	1.18 ±    0.45	1.91 ±    1.11		
     Median	1.21	1.08	1.56		
     Min, Max	0.53,    4.58	0.53,    2.55	0.58,    4.58		
    25, 75 Percentile	0.91,    1.73	0.86,    1.33	1.12,    2.57		
E wave deceleration time (msec)					
     N	155	94	61	0.1190	
     Mean ± SD	186.88 ±   58.05	192.74 ±   63.82	177.85 ±   46.88		
     Median	182.00	188.50	172.00		
     Min, Max	71.00,  434.00	71.00,  434.00	103.00,  310.00		
    25, 75 Percentile	151.00,  218.00	155.00,  225.00	144.00,  199.00		
NYHA FC, N (%)					
     I	26 (  1.8%)	16 (  1.3%)	10 (  4.1%)	<0.0001	
     II	73 (  5.0%)	29 (  2.4%)	44 ( 18.2%)		
     III	65 (  4.5%)	10 (  0.8%)	55 ( 22.7%)		
     IV	12 (  0.8%)	1 (  0.1%)	11 (  4.5%)		
     Missing	1,276 ( 87.9%)	1,154 ( 95.4%)	122 ( 50.4%)		
Cardiomyopathy/Cardiac Disorder, N (%)					
     Without symptom	1,097 ( 75.6%)	995 ( 82.2%)	102 ( 42.1%)	<0.0001	
     With symptom	355 ( 24.4%)	215 ( 17.8%)	140 ( 57.9%)		
Neuropathy, N (%)					
     Without symptom	500 ( 34.4%)	415 ( 34.3%)	85 ( 35.1%)	0.8049	
     With symptom	952 ( 65.6%)	795 ( 65.7%)	157 ( 64.9%)		
BNP >400 pg/mL, N (%)	138 ( 13.4%)	94 ( 10.0%)	44 ( 49.4%)	<0.0001	
NT-proBNP >1000 pg/mL, N (%)	112 ( 25.9%)	39 ( 14.1%)	73 ( 46.8%)	<0.0001	
Troponin I >0.1 ng/mL, N (%)	21 ( 33.9%)	2 ( 33.3%)	19 ( 33.9%)	0.9766	
Troponin T >0.1 ng/mL, N (%)	12 (  6.5%)	3 (  2.8%)	9 ( 11.5%)	0.0181	

 * Non-Val30Met excludes Wild Type.	
  History of liver transplant includes any liver transplant recorded in the THAOS database, both pre- and post-baseline.	
  NYHA FC is entered in place of severity when subjects report heart failure as a symptom.  Subjects who do not report heart failure are missing this information.	
 Notes: Baseline lab and echo values were selected using the values closest to consent within the baseline period (consent +/- six months).  The analytic cohort includes subjects who have baseline BNP and/or NT-BNP.	
