# Supplementary material for: Impact of genotype and phenotype on cardiac biomarkers in patients with transthyretin amyloidosis – Report from the Transthyretin Amyloidosis Outcome Survey (THAOS)
Source: PLoS One. 2017 Apr 6;12(4):e0173086. doi: 10.1371/journal.pone.0173086 (PMC5383030; doi:10.1371/journal.pone.0173086)
Supplement: S1 Supporting Information — (ZIP) [file pone.0173086.s001.zip › S2_Table_Q006_Table_5_v2.sas.rtf]

 Table 5. Comparison of Baseline Characteristics (Clinical, Biological, Echocardiography) Among Subjects with either Troponin I or Troponin T, by Troponin I/T Quartile*	

 	Quartile 1
(N = 138)	Quartile 2
(N = 48)	Quartile 3
(N = 94)	Quartile 4
(N = 87)	P-value	
Gender, N (%)						
     Male	74 ( 53.6%)	39 ( 81.3%)	77 ( 81.9%)	79 ( 90.8%)	<0.0001	
     Female	64 ( 46.4%)	9 ( 18.8%)	17 ( 18.1%)	8 (  9.2%)		
Age (yrs)						
     N	138	48	94	87	<0.0001	
     Mean ± SD	49.62 ±   17.86	65.92 ±   13.51	68.93 ±   10.09	72.05 ±    8.31		
     Median	48.58	68.29	71.11	72.57		
     Min, Max	19.90,   89.57	22.11,   85.85	34.81,   84.21	38.83,   87.27		
    25, 75 Percentile	35.71,   63.97	59.58,   77.20	64.22,   76.43	67.07,   77.54		
Race/Ethnicity, N (%)						
     Caucasian	83 ( 60.1%)	35 ( 72.9%)	77 ( 81.9%)	68 ( 78.2%)	<0.0001	
     African Descent	4 (  2.9%)	3 (  6.3%)	8 (  8.5%)	13 ( 14.9%)		
     Latino American	1 (  0.7%)	1 (  2.1%)	2 (  2.1%)	2 (  2.3%)		
     Asian	2 (  1.4%)	0 (  0.0%)	1 (  1.1%)	1 (  1.1%)		
     Other	1 (  0.7%)	0 (  0.0%)	4 (  4.3%)	2 (  2.3%)		
     Missing	47 ( 34.1%)	9 ( 18.8%)	2 (  2.1%)	1 (  1.1%)		
TTR genotype, N (%)						
     Wild Type	16 ( 11.6%)	19 ( 39.6%)	47 ( 50.0%)	43 ( 49.4%)	<0.0001	
     Val30Met	79 ( 57.2%)	12 ( 25.0%)	11 ( 11.7%)	9 ( 10.3%)		
     Non-Val30Met	43 ( 31.2%)	17 ( 35.4%)	36 ( 38.3%)	35 ( 40.2%)		
Age at onset of ATTR symptoms (yrs)						
     N	101	45	89	83	<0.0001	
     Mean ± SD	48.56 ±   16.19	59.72 ±   14.49	62.31 ±   12.23	64.62 ±   10.58		
     Median	48.12	63.24	64.34	65.97		
     Min, Max	19.33,   89.45	28.33,   81.50	28.05,   83.83	26.29,   84.85		
    25, 75 Percentile	35.46,   60.53	49.85,   69.72	54.50,   70.51	57.29,   71.28		
Age at measurement of BNP/NT-BNP (yrs)						
     N	138	48	94	87	<0.0001	
     Mean ± SD	49.62 ±   17.85	65.90 ±   13.53	68.92 ±   10.09	72.04 ±    8.29		
     Median	48.45	68.29	71.15	72.55		
     Min, Max	19.90,   89.57	22.10,   85.82	34.78,   84.21	38.83,   87.18		
    25, 75 Percentile	35.56,   63.85	59.59,   77.20	64.22,   76.43	66.92,   77.54		
Age at measurement of Troponin I/T (yrs)						
     N	138	48	94	87	<0.0001	
     Mean ± SD	49.63 ±   17.85	65.89 ±   13.53	68.90 ±   10.09	72.05 ±    8.29		
     Median	48.45	68.29	71.15	72.55		
     Min, Max	19.90,   89.57	22.10,   85.83	34.78,   84.21	38.83,   87.18		
    25, 75 Percentile	35.56,   63.85	59.59,   77.15	64.08,   76.43	67.27,   77.54		
Karnofsky index						
     N	116	30	80	59	<0.0001	
     Mean ± SD	90.52 ±   10.86	81.00 ±    9.23	76.25 ±   14.18	74.41 ±   14.89		
     Median	90.00	80.00	80.00	80.00		
     Min, Max	50.00,  100.00	60.00,  100.00	40.00,  100.00	20.00,  100.00		
    25, 75 Percentile	80.00,  100.00	80.00,   90.00	70.00,   90.00	70.00,   80.00		
History of liver transplant, N (%)						
     No liver transplant	112 ( 81.2%)	45 ( 93.8%)	88 ( 93.6%)	86 ( 98.9%)	<0.0001	
     Liver transplant	26 ( 18.8%)	3 (  6.3%)	6 (  6.4%)	1 (  1.1%)		
BNP (pg/mL)						
     N	65	32	13	26	0.0042	
     Mean ± SD	148.14 ±  165.90	501.67 ±  443.90	993.96 ± 1158.61	1,773.74 ± 4311.55		
     Median	85.10	361.85	642.00	809.25		
     Min, Max	5.80,  803.50	10.00, 1934.80	54.30, 4377.00	169.80,22400.00		
    25, 75 Percentile	31.70,  203.40	141.05,  821.95	470.00,  796.40	423.50, 1313.90		
NT-BNP (pg/mL)						
     N	75	17	82	62	0.0034	
     Mean ± SD	1,309.70 ± 5048.55	4,718.31 ±11698.23	6,253.47 ±11685.35	17401.57 ±47378.77		
     Median	99.00	1822.00	2307.00	4402.00		
     Min, Max	14.00,35277.55	46.00,49778.19	30.00,78542.31	1.00,296450.0		
    25, 75 Percentile	38.00,  387.00	1,290.00, 2457.00	1,424.00, 4729.00	2,583.00, 9408.00		
Troponin I (ng/mL)						
     N	29	23	27	29	<0.0001	
     Mean ± SD	0.02 ±    0.01	0.05 ±    0.01	0.12 ±    0.09	0.28 ±    0.26		
     Median	0.02	0.05	0.10	0.17		
     Min, Max	0.00,    0.06	0.04,    0.07	0.06,    0.58	0.06,    1.00		
    25, 75 Percentile	0.02,    0.03	0.05,    0.06	0.10,    0.12	0.13,    0.23		
Troponin T (ng/mL)						
     N	111	26	73	64	<0.0001	
     Mean ± SD	0.01 ±    0.00	0.02 ±    0.00	0.04 ±    0.01	0.11 ±    0.12		
     Median	0.01	0.02	0.04	0.08		
     Min, Max	0.00,    0.01	0.01,    0.03	0.03,    0.05	0.05,    1.00		
    25, 75 Percentile	0.00,    0.01	0.02,    0.02	0.03,    0.04	0.06,    0.12		
Creatinine (mg/dL)						
     N	136	47	93	86	0.0002	
     Mean ± SD	73.39 ±   18.43	90.05 ±   23.08	112.14 ±   49.29	169.89 ±  316.57		
     Median	71.00	87.00	107.85	125.97		
     Min, Max	1.63,  137.90	53.92,  147.63	41.55,  450.84	67.18, 3005.60		
    25, 75 Percentile	61.00,   83.05	71.60,  106.08	86.63,  123.76	100.78,  159.12		
Estimated GFR						
     N	136	46	88	86	0.0237	
     Mean ± SD	130.51 ±  339.30	77.36 ±   27.14	63.87 ±   24.87	50.00 ±   20.48		
     Median	100.95	71.35	61.20	50.65		
     Min, Max	0.00, 4040.30	36.40,  150.00	9.50,  154.50	0.00,  115.90		
    25, 75 Percentile	80.40,  119.30	58.60,   95.50	49.40,   75.70	37.50,   63.30		
Modified BMI						
     N	108	37	73	63	0.2116	
     Mean ± SD	1,097.87 ±  241.68	1,036.65 ±  195.09	1,044.23 ±  201.74	1,040.35 ±  204.03		
     Median	1107.69	1034.86	1027.48	1015.67		
     Min, Max	497.52, 1672.69	559.76, 1498.33	634.50, 1583.24	630.92, 1791.76		
    25, 75 Percentile	944.28, 1251.64	918.57, 1200.64	902.78, 1188.45	904.24, 1190.31		
Left atrium (mm)						
     N	69	25	68	59	<0.0001	
     Mean ± SD	38.13 ±    9.78	45.86 ±    8.89	45.03 ±    7.90	48.64 ±    7.56		
     Median	38.00	45.00	44.00	47.00		
     Min, Max	13.00,   64.00	30.00,   65.00	21.00,   63.00	35.00,   69.00		
    25, 75 Percentile	32.00,   43.00	42.00,   48.50	40.00,   50.50	43.00,   52.00		
LV septum (mm)						
     N	76	29	74	65	<0.0001	
     Mean ± SD	12.33 ±    3.99	16.36 ±    3.68	18.23 ±    4.26	19.37 ±    3.84		
     Median	12.00	16.00	18.00	20.00		
     Min, Max	6.00,   26.00	10.00,   25.00	6.00,   27.00	9.00,   29.00		
    25, 75 Percentile	9.00,   14.00	14.00,   19.00	16.00,   21.00	17.00,   21.00		
LV posterior wall (mm)						
     N	75	28	76	64	<0.0001	
     Mean ± SD	11.12 ±    3.46	14.60 ±    3.46	15.64 ±    3.37	17.66 ±    3.60		
     Median	10.00	14.00	16.00	18.00		
     Min, Max	4.00,   19.00	8.00,   21.00	6.00,   23.00	9.00,   26.00		
    25, 75 Percentile	9.00,   13.00	12.50,   17.00	14.00,   17.00	15.00,   20.00		
LV diastolic diameter (mm)						
     N	75	29	77	65	0.1185	
     Mean ± SD	45.49 ±    6.51	45.97 ±    5.57	43.51 ±    5.36	44.08 ±    6.75		
     Median	46.00	46.00	44.00	43.00		
     Min, Max	29.00,   65.00	37.00,   55.00	30.00,   57.00	32.00,   67.00		
    25, 75 Percentile	41.00,   50.00	42.00,   50.00	40.00,   48.00	40.00,   47.00		
LV systolic diameter (mm)						
     N	69	24	70	61	<0.0001	
     Mean ± SD	28.20 ±    6.08	33.75 ±    6.30	32.39 ±    6.94	33.54 ±    6.85		
     Median	29.00	33.50	32.00	32.00		
     Min, Max	11.00,   47.00	21.00,   42.10	18.00,   50.00	23.00,   55.00		
    25, 75 Percentile	25.00,   31.00	29.50,   39.00	27.00,   37.00	28.00,   38.00		
End diastolic volume (mL)						
     N	0	2	2	1	0.1598	
     Mean ± SD		139.50 ±    2.12	107.50 ±   33.23	46.00 ±     .		
     Median		139.50	107.50	46.00		
     Min, Max		138.00,  141.00	84.00,  131.00	46.00,   46.00		
    25, 75 Percentile		138.00,  141.00	84.00,  131.00	46.00,   46.00		
End systolic volume (mL)						
     N	0	2	1	1	0.1181	
     Mean ± SD		74.50 ±    4.95	51.00 ±     .	24.00 ±     .		
     Median		74.50	51.00	24.00		
     Min, Max		71.00,   78.00	51.00,   51.00	24.00,   24.00		
    25, 75 Percentile		71.00,   78.00	51.00,   51.00	24.00,   24.00		
Stroke volume index						
     N	24	12	18	18	0.0849	
     Mean ± SD	77.42 ±   24.36	66.33 ±   17.35	60.89 ±   24.55	61.11 ±   25.63		
     Median	77.50	67.00	57.50	59.00		
     Min, Max	28.00,  121.00	33.00,  104.00	17.00,  103.00	23.00,  106.00		
    25, 75 Percentile	60.50,   96.50	59.50,   70.50	43.00,   77.00	39.00,   73.00		
LV ejection fraction (%)						
     N	69	24	71	63	<0.0001	
     Mean ± SD	56.59 ±   10.63	45.25 ±   15.83	44.90 ±   13.33	40.08 ±   12.41		
     Median	60.00	46.50	45.00	40.00		
     Min, Max	22.00,   76.00	20.00,   71.00	15.00,   74.00	10.00,   65.00		
    25, 75 Percentile	55.00,   61.00	30.00,   60.00	35.00,   55.00	30.00,   49.00		
E/A ratio						
     N	37	8	7	12	0.0232	
     Mean ± SD	1.48 ±    0.83	2.36 ±    1.17	1.40 ±    0.85	2.28 ±    1.34		
     Median	1.33	2.55	1.01	2.27		
     Min, Max	0.62,    4.30	0.70,    3.68	0.77,    3.15	0.58,    4.50		
    25, 75 Percentile	0.98,    1.59	1.24,    3.45	0.86,    1.80	1.07,    3.30		
E wave deceleration time (msec)						
     N	39	9	28	28	0.0523	
     Mean ± SD	204.67 ±   64.77	217.78 ±   60.05	189.07 ±   42.51	171.39 ±   50.13		
     Median	189.00	190.00	191.00	160.00		
     Min, Max	103.00,  434.00	164.00,  321.00	120.00,  261.00	102.00,  300.00		
    25, 75 Percentile	163.00,  241.00	175.00,  250.00	156.50,  220.00	136.00,  202.50		
NYHA FC, N (%)						
     I	5 (  3.6%)	1 (  2.1%)	5 (  5.3%)	5 (  5.7%)	<0.0001	
     II	16 ( 11.6%)	15 ( 31.3%)	40 ( 42.6%)	29 ( 33.3%)		
     III	7 (  5.1%)	6 ( 12.5%)	24 ( 25.5%)	33 ( 37.9%)		
     IV	0 (  0.0%)	1 (  2.1%)	0 (  0.0%)	0 (  0.0%)		
     Missing	110 ( 79.7%)	25 ( 52.1%)	25 ( 26.6%)	20 ( 23.0%)		
Cardiomyopathy/Cardiac Disorder, N (%)						
     Without symptom	99 ( 71.7%)	19 ( 39.6%)	15 ( 16.0%)	14 ( 16.1%)	<0.0001	
     With symptom	39 ( 28.3%)	29 ( 60.4%)	79 ( 84.0%)	73 ( 83.9%)		
Neuropathy, N (%)						
     Without symptom	60 ( 43.5%)	14 ( 29.2%)	33 ( 35.1%)	39 ( 44.8%)	0.1849	
     With symptom	78 ( 56.5%)	34 ( 70.8%)	61 ( 64.9%)	48 ( 55.2%)		

 * Troponin I/T quartile assignment is based on the quartile values of the available measure.  In the case of subjects with both Troponin I and T available, Troponin T quartile was used.	
  History of liver transplant includes any liver transplant recorded in the THAOS database, both pre- and post-baseline.	
  NYHA FC is entered in place of severity when subjects report heart failure as a symptom.  Subjects who do not report heart failure are missing this information.	
 Notes: Baseline lab and echo values were selected using the values closest to consent within the baseline period (consent +/- six months).  The analytic cohort includes subjects who have baseline BNP and/or NT-BNP.	
