# Supplementary material for: Impact of genotype and phenotype on cardiac biomarkers in patients with transthyretin amyloidosis – Report from the Transthyretin Amyloidosis Outcome Survey (THAOS)
Source: PLoS One. 2017 Apr 6;12(4):e0173086. doi: 10.1371/journal.pone.0173086 (PMC5383030; doi:10.1371/journal.pone.0173086)
Supplement: S1 Supporting Information — (ZIP) [file pone.0173086.s001.zip › S3_Table_Q011_Table_10_v2.sas.rtf]

 Table 10. Comparison of Baseline Characteristics (Clinical, Biological, Echocardiography) Among Subjects with either BNP or NT-BNP, by BNP/NT-BNP Quartile*	

 	Quartile 1
(N = 407)	Quartile 2
(N = 404)	Quartile 3
(N = 402)	Quartile 4
(N = 404)	P-value	
Gender, N (%)						
     Male	256 ( 62.9%)	157 ( 38.9%)	203 ( 50.5%)	265 ( 65.6%)	<0.0001	
     Female	151 ( 37.1%)	247 ( 61.1%)	199 ( 49.5%)	139 ( 34.4%)		
Age (yrs)						
     N	407	404	402	404	<0.0001	
     Mean ± SD	37.00 ±   12.50	40.65 ±   13.46	50.76 ±   18.16	63.75 ±   14.49		
     Median	34.44	36.95	47.91	66.91		
     Min, Max	18.34,   83.36	18.46,   79.90	19.21,   89.53	24.68,   89.57		
    25, 75 Percentile	27.32,   44.79	30.77,   48.12	34.87,   67.79	54.43,   74.68		
Race/Ethnicity, N (%)						
     Caucasian	78 ( 19.2%)	68 ( 16.8%)	137 ( 34.1%)	211 ( 52.2%)	<0.0001	
     African Descent	3 (  0.7%)	0 (  0.0%)	10 (  2.5%)	37 (  9.2%)		
     Latino American	0 (  0.0%)	0 (  0.0%)	1 (  0.2%)	6 (  1.5%)		
     Asian	13 (  3.2%)	5 (  1.2%)	14 (  3.5%)	8 (  2.0%)		
     Other	2 (  0.5%)	0 (  0.0%)	5 (  1.2%)	4 (  1.0%)		
     Missing	311 ( 76.4%)	331 ( 81.9%)	235 ( 58.5%)	138 ( 34.2%)		
TTR genotype, N (%)						
     Wild Type	2 (  0.5%)	0 (  0.0%)	50 ( 12.4%)	113 ( 28.0%)	<0.0001	
     Val30Met	366 ( 89.9%)	368 ( 91.1%)	290 ( 72.1%)	186 ( 46.0%)		
     Non-Val30Met	39 (  9.6%)	36 (  8.9%)	62 ( 15.4%)	105 ( 26.0%)		
Age at onset of ATTR symptoms (yrs)						
     N	255	316	352	386	<0.0001	
     Mean ± SD	36.00 ±   11.74	38.67 ±   12.74	46.02 ±   16.93	55.49 ±   16.11		
     Median	33.47	34.49	44.93	57.47		
     Min, Max	17.73,   69.68	9.72,   76.54	16.77,   87.76	17.79,   89.45		
    25, 75 Percentile	27.37,   42.65	29.78,   46.06	30.47,   61.50	43.09,   68.18		
Age at measurement of BNP/NT-BNP (yrs)						
     N	407	404	402	404	<0.0001	
     Mean ± SD	37.01 ±   12.51	40.65 ±   13.46	50.77 ±   18.14	63.76 ±   14.48		
     Median	34.44	36.95	47.91	66.91		
     Min, Max	18.34,   83.34	18.46,   80.04	19.21,   89.53	24.68,   89.57		
    25, 75 Percentile	27.34,   44.92	30.77,   48.12	34.96,   67.78	54.42,   74.76		
Age at measurement of Troponin I/T (yrs)						
     N	54	45	98	170	<0.0001	
     Mean ± SD	43.50 ±   13.27	50.26 ±   17.50	64.58 ±   14.89	69.51 ±   11.78		
     Median	43.93	52.28	68.90	71.69		
     Min, Max	19.90,   68.41	19.98,   80.04	23.39,   89.53	30.13,   89.57		
    25, 75 Percentile	33.27,   54.25	36.42,   64.37	57.17,   73.70	64.53,   77.01		
Karnofsky index						
     N	384	387	349	331	<0.0001	
     Mean ± SD	94.77 ±    9.69	90.65 ±   11.42	84.79 ±   13.05	74.08 ±   14.71		
     Median	100.00	90.00	90.00	80.00		
     Min, Max	0.00,  100.00	30.00,  100.00	40.00,  100.00	10.00,  100.00		
    25, 75 Percentile	90.00,  100.00	80.00,  100.00	80.00,   90.00	70.00,   80.00		
History of liver transplant, N (%)						
     No liver transplant	372 ( 91.4%)	339 ( 83.9%)	323 ( 80.3%)	334 ( 82.7%)	<0.0001	
     Liver transplant	35 (  8.6%)	65 ( 16.1%)	79 ( 19.7%)	70 ( 17.3%)		
BNP (pg/mL)						
     N	270	271	269	269	<0.0001	
     Mean ± SD	17.59 ±    7.34	47.13 ±   10.72	115.79 ±   36.50	1,329.69 ± 3073.18		
     Median	18.35	45.40	108.90	504.00		
     Min, Max	4.00,   30.50	30.60,   68.00	68.10,  194.90	195.00,32434.00		
    25, 75 Percentile	11.80,   23.60	38.00,   56.00	84.00,  146.00	317.00,  948.00		
NT-BNP (pg/mL)						
     N	139	136	136	139	<0.0001	
     Mean ± SD	38.91 ±   16.78	166.43 ±   82.69	1,342.26 ±  692.59	15332.37 ±34151.89		
     Median	36.00	139.00	1239.00	5291.00		
     Min, Max	1.00,   73.00	74.00,  358.00	338.80, 2584.00	81.00,296450.0		
    25, 75 Percentile	24.00,   53.00	98.00,  219.11	736.50, 1915.50	3,711.00,13732.00		
Troponin I (ng/mL)						
     N	7	6	21	74	0.0873	
     Mean ± SD	0.18 ±    0.36	0.02 ±    0.01	0.06 ±    0.05	0.14 ±    0.17		
     Median	0.06	0.02	0.04	0.10		
     Min, Max	0.01,    1.00	0.00,    0.03	0.01,    0.20	0.00,    0.86		
    25, 75 Percentile	0.01,    0.10	0.01,    0.02	0.02,    0.10	0.05,    0.14		
Troponin T (ng/mL)						
     N	47	39	80	108	0.0026	
     Mean ± SD	0.03 ±    0.14	0.02 ±    0.03	0.03 ±    0.03	0.06 ±    0.05		
     Median	0.01	0.01	0.03	0.05		
     Min, Max	0.00,    1.00	0.00,    0.20	0.00,    0.14	0.00,    0.26		
    25, 75 Percentile	0.01,    0.01	0.00,    0.01	0.01,    0.04	0.03,    0.07		
Creatinine (mg/dL)						
     N	396	394	389	396	<0.0001	
     Mean ± SD	74.49 ±   48.54	67.04 ±   15.32	78.13 ±   30.38	139.89 ±  357.21		
     Median	71.60	65.42	70.72	99.89		
     Min, Max	32.71,  981.24	26.52,  134.37	1.63,  396.03	34.48, 6011.20		
    25, 75 Percentile	61.94,   81.33	55.69,   76.91	60.11,   88.40	71.30,  128.09		
Estimated GFR						
     N	395	393	384	393	<0.0001	
     Mean ± SD	126.33 ±   72.98	114.56 ±   35.93	110.76 ±  221.27	66.48 ±   35.34		
     Median	119.40	111.50	94.25	60.40		
     Min, Max	0.00, 1380.00	0.00,  307.70	0.00, 4040.30	0.00,  331.80		
    25, 75 Percentile	103.30,  143.50	92.00,  131.00	71.85,  117.25	45.20,   82.60		
Modified BMI						
     N	371	377	347	324	<0.0001	
     Mean ± SD	1,180.42 ±  234.67	1,086.36 ±  206.95	1,035.47 ±  228.77	995.11 ±  234.49		
     Median	1150.57	1067.07	1017.09	982.94		
     Min, Max	448.10, 2094.79	530.02, 1871.65	497.52, 2001.17	413.82, 1905.29		
    25, 75 Percentile	1,024.27, 1294.17	948.24, 1218.04	881.14, 1164.65	831.36, 1145.08		
Left atrium (mm)						
     N	71	60	106	171	<0.0001	
     Mean ± SD	36.36 ±   17.39	37.09 ±    7.61	43.55 ±    8.36	45.89 ±    8.37		
     Median	35.00	35.85	43.00	45.00		
     Min, Max	17.00,  170.00	24.70,   64.00	28.00,   63.00	13.00,   69.00		
    25, 75 Percentile	31.00,   38.00	32.00,   41.85	37.00,   48.00	41.00,   50.00		
LV septum (mm)						
     N	86	69	127	192	<0.0001	
     Mean ± SD	10.41 ±    2.52	11.92 ±    3.76	16.54 ±    4.36	18.15 ±    4.27		
     Median	10.00	11.00	17.00	18.00		
     Min, Max	6.00,   19.00	6.00,   25.00	7.00,   27.00	2.30,   29.00		
    25, 75 Percentile	9.00,   12.00	9.90,   14.00	13.00,   20.00	16.00,   21.00		
LV posterior wall (mm)						
     N	83	67	128	184	<0.0001	
     Mean ± SD	9.41 ±    2.09	10.23 ±    2.53	14.42 ±    3.83	15.84 ±    3.84		
     Median	9.00	10.00	15.00	16.00		
     Min, Max	4.90,   15.00	4.00,   19.00	6.00,   23.00	2.20,   26.00		
    25, 75 Percentile	8.00,   10.80	8.10,   11.90	12.00,   17.00	13.00,   19.00		
LV diastolic diameter (mm)						
     N	77	64	120	190	0.4524	
     Mean ± SD	45.74 ±    5.92	45.95 ±    5.20	44.68 ±    5.64	44.91 ±    7.22		
     Median	46.00	45.00	45.00	45.00		
     Min, Max	28.00,   57.00	35.00,   56.00	32.00,   59.00	3.60,   67.00		
    25, 75 Percentile	42.00,   50.00	42.00,   50.50	41.00,   49.00	41.00,   49.00		
LV systolic diameter (mm)						
     N	73	54	111	169	<0.0001	
     Mean ± SD	28.77 ±    5.50	28.13 ±    6.32	31.20 ±    6.80	32.92 ±    7.91		
     Median	29.00	27.35	31.00	32.00		
     Min, Max	11.00,   42.00	6.00,   42.00	2.50,   52.00	3.00,   61.00		
    25, 75 Percentile	25.00,   31.00	24.00,   31.00	27.00,   36.00	28.00,   38.00		
End diastolic volume (mL)						
     N	0	0	2	5	0.5246	
     Mean ± SD			122.50 ±   12.02	102.00 ±   39.62		
     Median			122.50	101.00		
     Min, Max			114.00,  131.00	46.00,  141.00		
    25, 75 Percentile			114.00,  131.00	84.00,  138.00		
End systolic volume (mL)						
     N	0	0	1	5	0.7728	
     Mean ± SD			52.00 ±     .	59.60 ±   22.46		
     Median			52.00	71.00		
     Min, Max			52.00,   52.00	24.00,   78.00		
    25, 75 Percentile			52.00,   52.00	51.00,   74.00		
Stroke volume index						
     N	30	32	56	73	0.2339	
     Mean ± SD	72.07 ±   14.52	75.28 ±   23.83	67.14 ±   19.89	66.92 ±   24.56		
     Median	71.50	71.00	66.50	66.00		
     Min, Max	45.00,  105.00	30.00,  121.00	27.00,  111.00	17.00,  127.00		
    25, 75 Percentile	62.00,   83.00	57.00,   95.50	53.50,   81.50	52.00,   84.00		
LV ejection fraction (%)						
     N	65	51	106	160	<0.0001	
     Mean ± SD	60.51 ±    9.33	59.96 ±    7.76	50.80 ±   13.42	42.72 ±   14.22		
     Median	60.00	60.00	54.00	40.00		
     Min, Max	30.00,   78.00	40.00,   76.00	20.00,   83.00	10.00,   80.00		
    25, 75 Percentile	58.00,   65.00	55.00,   65.00	43.00,   60.00	35.00,   55.00		
E/A ratio						
     N	36	32	42	33	<0.0001	
     Mean ± SD	1.18 ±    0.43	1.12 ±    0.31	1.65 ±    0.86	2.45 ±    1.24		
     Median	1.13	1.10	1.52	2.38		
     Min, Max	0.58,    2.55	0.62,    2.00	0.25,    3.50	0.63,    4.58		
    25, 75 Percentile	0.91,    1.38	0.90,    1.33	1.00,    2.00	1.61,    3.39		
E wave deceleration time (msec)						
     N	42	37	64	68	0.4835	
     Mean ± SD	181.98 ±   44.12	193.89 ±   71.08	191.25 ±   57.19	179.44 ±   51.40		
     Median	185.00	188.00	181.00	172.00		
     Min, Max	92.00,  281.00	71.00,  434.00	74.00,  353.00	102.00,  321.00		
    25, 75 Percentile	155.00,  211.00	155.00,  234.00	155.50,  231.50	140.00,  213.50		
NYHA FC, N (%)						
     I	1 (  0.2%)	5 (  1.2%)	15 (  3.7%)	16 (  4.0%)	<0.0001	
     II	1 (  0.2%)	1 (  0.2%)	47 ( 11.7%)	97 ( 24.0%)		
     III	2 (  0.5%)	1 (  0.2%)	24 (  6.0%)	93 ( 23.0%)		
     IV	0 (  0.0%)	2 (  0.5%)	5 (  1.2%)	7 (  1.7%)		
     Missing	403 ( 99.0%)	395 ( 97.8%)	311 ( 77.4%)	191 ( 47.3%)		
Cardiomyopathy/Cardiac Disorder, N (%)						
     Without symptom	383 ( 94.1%)	360 ( 89.1%)	257 ( 63.9%)	110 ( 27.2%)	<0.0001	
     With symptom	24 (  5.9%)	44 ( 10.9%)	145 ( 36.1%)	294 ( 72.8%)		
Neuropathy, N (%)						
     Without symptom	215 ( 52.8%)	158 ( 39.1%)	109 ( 27.1%)	97 ( 24.0%)	<0.0001	
     With symptom	192 ( 47.2%)	246 ( 60.9%)	293 ( 72.9%)	307 ( 76.0%)		

 * BNP/NT-BNP quartile assignment is based on the quartile values of the available measure.  In the case of subjects with both BNP and NT-BNP available, BNP quartile was used.	
  History of liver transplant includes any liver transplant recorded in the THAOS database, both pre- and post-baseline.	
  NYHA FC is entered in place of severity when subjects report heart failure as a symptom.  Subjects who do not report heart failure are missing this information.	
 Notes: Baseline lab and echo values were selected using the values closest to consent within the baseline period (consent +/- six months).  The analytic cohort includes subjects who have baseline BNP and/or NT-BNP.	
