# Supplementary material for: Impact of genotype and phenotype on cardiac biomarkers in patients with transthyretin amyloidosis – Report from the Transthyretin Amyloidosis Outcome Survey (THAOS)
Source: PLoS One. 2017 Apr 6;12(4):e0173086. doi: 10.1371/journal.pone.0173086 (PMC5383030; doi:10.1371/journal.pone.0173086)
Supplement: S1 Supporting Information — (ZIP) [file pone.0173086.s001.zip › S4_Table_Q014_Table_11_v2.sas.rtf]

 Table 11. Comparison of Baseline Characteristics (Clinical, Biological, Echocardiography), Wild Type vs. TTR Mutation	

 	Overall
(N = 1617)	Wild Type
(N = 165)	TTR Mutation
(N = 1452)	P-value
Wild type vs. TTR Mutation	
Gender, N (%)					
     Male	881 ( 54.5%)	157 ( 95.2%)	724 ( 49.9%)	<0.0001	
     Female	736 ( 45.5%)	8 (  4.8%)	728 ( 50.1%)		
Age (yrs)					
     N	1617	165	1452	<0.0001	
     Mean ± SD	48.02 ±   18.07	75.43 ±    6.79	44.90 ±   16.23		
     Median	43.91	75.27	41.46		
     Min, Max	18.34,   89.57	48.03,   89.57	18.34,   86.23		
    25, 75 Percentile	32.97,   64.38	71.27,   80.37	31.90,   57.73		
Race/Ethnicity, N (%)					
     Caucasian	494 ( 30.6%)	155 ( 93.9%)	339 ( 23.3%)	<0.0001	
     African Descent	50 (  3.1%)	3 (  1.8%)	47 (  3.2%)		
     Latino American	7 (  0.4%)	1 (  0.6%)	6 (  0.4%)		
     Asian	40 (  2.5%)	2 (  1.2%)	38 (  2.6%)		
     Other	11 (  0.7%)	1 (  0.6%)	10 (  0.7%)		
     Missing	1,015 ( 62.8%)	3 (  1.8%)	1,012 ( 69.7%)		
TTR genotype, N (%)					
     Wild Type	165 ( 10.2%)	165 (100.0%)	0 (  0.0%)	<0.0001	
     Val30Met	1,210 ( 74.8%)	0 (  0.0%)	1,210 ( 83.3%)		
     Non-Val30Met	242 ( 15.0%)	0 (  0.0%)	242 ( 16.7%)		
Age at onset of ATTR symptoms (yrs)					
     N	1309	158	1151	<0.0001	
     Mean ± SD	45.09 ±   16.66	67.13 ±   11.40	42.06 ±   14.89		
     Median	41.70	68.81	37.99		
     Min, Max	9.72,   89.45	32.88,   89.45	9.72,   81.95		
    25, 75 Percentile	30.97,   59.40	61.80,   75.54	29.82,   54.50		
Age at measurement of BNP/NT-BNP (yrs)					
     N	1617	165	1452	<0.0001	
     Mean ± SD	48.02 ±   18.07	75.42 ±    6.78	44.91 ±   16.22		
     Median	43.95	75.29	41.46		
     Min, Max	18.34,   89.57	48.03,   89.57	18.34,   86.22		
    25, 75 Percentile	32.95,   64.40	71.24,   80.37	31.90,   57.72		
Age at measurement of Troponin I/T (yrs)					
     N	367	125	242	<0.0001	
     Mean ± SD	62.01 ±   16.75	75.42 ±    6.20	55.08 ±   16.27		
     Median	67.01	75.10	57.04		
     Min, Max	19.90,   89.57	60.43,   89.57	19.90,   85.27		
    25, 75 Percentile	51.55,   74.58	71.27,   80.16	42.42,   68.16		
Karnofsky index					
     N	1451	106	1345	<0.0001	
     Mean ± SD	86.55 ±   14.43	77.92 ±   11.44	87.23 ±   14.43		
     Median	90.00	80.00	90.00		
     Min, Max	0.00,  100.00	40.00,  100.00	0.00,  100.00		
    25, 75 Percentile	80.00,  100.00	70.00,   90.00	80.00,  100.00		
History of liver transplant*, N (%)					
     No liver transplant	1,368 ( 84.6%)	165 (100.0%)	1,203 ( 82.9%)	<0.0001	
     Liver transplant	249 ( 15.4%)	0 (  0.0%)	249 ( 17.1%)		
BNP (pg/mL)					
     N	1079	49	1030	0.0091	
     Mean ± SD	376.61 ± 1628.36	969.19 ± 3141.62	348.41 ± 1516.55		
     Median	68.00	455.00	63.05		
     Min, Max	4.00,32434.00	92.70,22400.00	4.00,32434.00		
    25, 75 Percentile	30.50,  194.90	322.50,  645.00	28.90,  168.20		
NT-BNP (pg/mL)					
     N	550	118	432	0.0009	
     Mean ± SD	4,257.80 ±18306.08	9,184.66 ±18441.43	2,912.03 ±18057.65		
     Median	337.90	3348.50	154.00		
     Min, Max	1.00,296450.0	25.00,118139.6	1.00,296450.0		
    25, 75 Percentile	73.00, 2584.00	1,910.00, 6222.00	56.00, 1149.00		
Troponin I (ng/mL)					
     N	108	46	62	0.1006	
     Mean ± SD	0.12 ±    0.17	0.09 ±    0.06	0.14 ±    0.22		
     Median	0.08	0.08	0.07		
     Min, Max	0.00,    1.00	0.02,    0.26	0.00,    1.00		
    25, 75 Percentile	0.04,    0.13	0.05,    0.12	0.03,    0.13		
Troponin T (ng/mL)					
     N	274	90	184	0.0523	
     Mean ± SD	0.04 ±    0.07	0.05 ±    0.04	0.03 ±    0.08		
     Median	0.03	0.05	0.01		
     Min, Max	0.00,    1.00	0.01,    0.26	0.00,    1.00		
    25, 75 Percentile	0.01,    0.05	0.03,    0.06	0.01,    0.04		
Creatinine (mg/dL)					
     N	1575	161	1414	<0.0001	
     Mean ± SD	89.97 ±  183.72	150.58 ±  295.11	83.07 ±  165.14		
     Median	72.49	109.62	70.72		
     Min, Max	1.63, 6011.20	61.88, 3005.60	1.63, 6011.20		
    25, 75 Percentile	61.00,   90.00	96.36,  132.60	59.23,   84.86		
Estimated GFR					
     N	1565	157	1408	<0.0001	
     Mean ± SD	104.52 ±  120.36	55.72 ±   21.10	109.97 ±  125.54		
     Median	100.80	55.20	105.05		
     Min, Max	0.00, 4040.30	0.00,  147.60	0.00, 4040.30		
    25, 75 Percentile	71.80,  126.00	43.90,   65.40	79.60,  128.05		
Modified BMI					
     N	1419	116	1303	0.8951	
     Mean ± SD	1,077.67 ±  236.22	1,080.44 ±  191.33	1,077.43 ±  239.87		
     Median	1054.62	1080.43	1054.06		
     Min, Max	413.82, 2094.79	641.74, 1583.24	413.82, 2094.79		
    25, 75 Percentile	923.30, 1207.53	968.74, 1206.11	918.27, 1208.33		
Left atrium (mm)					
     N	408	106	302	<0.0001	
     Mean ± SD	42.33 ±   11.12	48.57 ±    8.36	40.14 ±   11.15		
     Median	41.00	47.00	39.00		
     Min, Max	13.00,  170.00	25.80,   69.00	13.00,  170.00		
    25, 75 Percentile	35.35,   47.00	43.00,   55.00	34.00,   45.00		
LV septum (mm)					
     N	474	121	353	<0.0001	
     Mean ± SD	15.41 ±    5.03	18.44 ±    3.54	14.37 ±    5.05		
     Median	16.00	18.00	13.00		
     Min, Max	2.30,   29.00	9.70,   29.00	2.30,   29.00		
    25, 75 Percentile	11.00,   19.00	16.00,   21.00	10.00,   18.00		
LV posterior wall (mm)					
     N	462	123	339	<0.0001	
     Mean ± SD	13.48 ±    4.31	16.30 ±    3.01	12.45 ±    4.25		
     Median	13.00	16.00	12.00		
     Min, Max	2.20,   26.00	9.60,   24.00	2.20,   26.00		
    25, 75 Percentile	10.00,   17.00	14.00,   18.00	9.00,   16.00		
LV diastolic diameter (mm)					
     N	451	124	327	0.5032	
     Mean ± SD	45.14 ±    6.34	44.81 ±    5.77	45.26 ±    6.55		
     Median	45.00	45.00	45.00		
     Min, Max	3.60,   67.00	30.00,   67.00	3.60,   67.00		
    25, 75 Percentile	41.00,   49.00	41.00,   48.00	41.00,   50.00		
LV systolic diameter (mm)					
     N	407	114	293	<0.0001	
     Mean ± SD	31.07 ±    7.25	34.09 ±    6.84	29.89 ±    7.07		
     Median	30.00	34.00	29.00		
     Min, Max	2.50,   61.00	18.00,   61.00	2.50,   55.00		
    25, 75 Percentile	26.70,   35.00	29.00,   38.00	25.00,   34.00		
End diastolic volume (mL)					
     N	7	5	2	0.3917	
     Mean ± SD	107.86 ±   34.21	115.60 ±   24.30	88.50 ±   60.10		
     Median	114.00	114.00	88.50		
     Min, Max	46.00,  141.00	84.00,  141.00	46.00,  131.00		
    25, 75 Percentile	84.00,  138.00	101.00,  138.00	46.00,  131.00		
End systolic volume (mL)					
     N	6	5	1	0.0420	
     Mean ± SD	58.33 ±   20.32	65.20 ±   12.76	24.00 ±     .		
     Median	61.50	71.00	24.00		
     Min, Max	24.00,   78.00	51.00,   78.00	24.00,   24.00		
    25, 75 Percentile	51.00,   74.00	52.00,   74.00	24.00,   24.00		
Stroke volume index					
     N	191	42	149	0.0313	
     Mean ± SD	69.19 ±   21.88	62.79 ±   20.42	71.00 ±   22.00		
     Median	69.00	62.00	70.00		
     Min, Max	17.00,  127.00	17.00,  103.00	23.00,  127.00		
    25, 75 Percentile	54.00,   84.00	49.00,   74.00	56.00,   87.00		
LV ejection fraction (%)					
     N	382	120	262	<0.0001	
     Mean ± SD	50.29 ±   14.53	43.02 ±   12.89	53.62 ±   14.03		
     Median	55.00	43.00	56.00		
     Min, Max	10.00,   83.00	10.00,   74.00	15.00,   83.00		
    25, 75 Percentile	40.00,   60.00	35.00,   55.00	45.00,   63.00		
E/A ratio					
     N	143	21	122	<0.0001	
     Mean ± SD	1.60 ±    0.94	2.35 ±    1.08	1.47 ±    0.86		
     Median	1.33	2.38	1.21		
     Min, Max	0.25,    4.58	0.25,    4.30	0.53,    4.58		
    25, 75 Percentile	0.94,    2.00	1.50,    3.00	0.91,    1.73		
E wave deceleration time (msec)					
     N	211	56	155	0.7224	
     Mean ± SD	186.06 ±   55.74	183.79 ±   49.19	186.88 ±   58.05		
     Median	181.00	175.00	182.00		
     Min, Max	71.00,  434.00	102.00,  321.00	71.00,  434.00		
    25, 75 Percentile	151.00,  215.00	154.00,  214.00	151.00,  218.00		
NYHA FC, N (%)					
     I	37 (  2.3%)	11 (  6.7%)	26 (  1.8%)	<0.0001	
     II	146 (  9.0%)	73 ( 44.2%)	73 (  5.0%)		
     III	120 (  7.4%)	55 ( 33.3%)	65 (  4.5%)		
     IV	14 (  0.9%)	2 (  1.2%)	12 (  0.8%)		
     Missing	1,300 ( 80.4%)	24 ( 14.5%)	1,276 ( 87.9%)		
Cardiomyopathy/Cardiac Disorder, N (%)					
     Without symptom	1,110 ( 68.6%)	13 (  7.9%)	1,097 ( 75.6%)	<0.0001	
     With symptom	507 ( 31.4%)	152 ( 92.1%)	355 ( 24.4%)		
Neuropathy, N (%)					
     Without symptom	579 ( 35.8%)	79 ( 47.9%)	500 ( 34.4%)	0.0006	
     With symptom	1,038 ( 64.2%)	86 ( 52.1%)	952 ( 65.6%)		

 * History of liver transplant includes any liver transplant recorded in the THAOS database, both pre- and post-baseline.	
  NYHA FC is entered in place of severity when subjects report heart failure as a symptom.  Subjects who do not report heart failure are missing this information.	
 Notes: Baseline lab and echo values were selected using the values closest to consent within the baseline period (consent +/- six months).  The analytic cohort includes subjects who have baseline BNP and/or NT-BNP.	
