# Supplementary material for: Impact of genotype and phenotype on cardiac biomarkers in patients with transthyretin amyloidosis – Report from the Transthyretin Amyloidosis Outcome Survey (THAOS)
Source: PLoS One. 2017 Apr 6;12(4):e0173086. doi: 10.1371/journal.pone.0173086 (PMC5383030; doi:10.1371/journal.pone.0173086)
Supplement: S1 Supporting Information — (ZIP) [file pone.0173086.s001.zip › S5_Table_Q015_Table_12_v2.sas.rtf]

 Table 12. Comparison of Baseline Characteristics (Clinical, Biological, Echocardiography), Symptomatic Subjects with Val30Met Mutation, Early vs. Late Onset	

 	Overall
(N = 828)	Early Onset
(N = 615)	Late Onset
(N = 213)	P-value
Early vs. Late Onset	
Gender, N (%)					
     Male	422 ( 51.0%)	303 ( 49.3%)	119 ( 55.9%)	0.0968	
     Female	406 ( 49.0%)	312 ( 50.7%)	94 ( 44.1%)		
Age (yrs)					
     N	828	615	213	<0.0001	
     Mean ± SD	45.53 ±   15.13	38.39 ±    9.54	66.13 ±    7.41		
     Median	41.75	36.98	66.94		
     Min, Max	19.21,   86.23	19.21,   78.30	51.52,   86.23		
    25, 75 Percentile	33.74,   56.84	31.56,   43.75	60.62,   71.85		
Race/Ethnicity, N (%)					
     Caucasian	162 ( 19.6%)	64 ( 10.4%)	98 ( 46.0%)	<0.0001	
     African Descent	1 (  0.1%)	0 (  0.0%)	1 (  0.5%)		
     Latino American	1 (  0.1%)	0 (  0.0%)	1 (  0.5%)		
     Asian	28 (  3.4%)	17 (  2.8%)	11 (  5.2%)		
     Other	4 (  0.5%)	3 (  0.5%)	1 (  0.5%)		
     Missing	632 ( 76.3%)	531 ( 86.3%)	101 ( 47.4%)		
TTR genotype, N (%)					
     Val30Met	828 (100.0%)	615 (100.0%)	213 (100.0%)		
Age at onset of ATTR symptoms (yrs)					
     N	828	615	213	<0.0001	
     Mean ± SD	39.85 ±   14.16	32.73 ±    7.54	60.43 ±    6.70		
     Median	35.13	32.24	59.79		
     Min, Max	9.72,   81.95	9.72,   49.92	50.09,   81.95		
    25, 75 Percentile	28.91,   50.50	27.40,   37.42	55.03,   65.13		
Age at measurement of BNP/NT-BNP (yrs)					
     N	828	615	213	<0.0001	
     Mean ± SD	45.54 ±   15.12	38.41 ±    9.54	66.13 ±    7.41		
     Median	41.81	36.98	66.97		
     Min, Max	19.21,   86.22	19.21,   78.30	51.52,   86.22		
    25, 75 Percentile	33.76,   56.93	31.56,   43.75	60.62,   71.85		
Age at measurement of Troponin I/T (yrs)					
     N	79	47	32	<0.0001	
     Mean ± SD	53.66 ±   15.68	43.30 ±   10.16	68.87 ±    8.01		
     Median	51.46	42.10	68.95		
     Min, Max	23.20,   80.25	23.20,   78.30	51.52,   80.25		
    25, 75 Percentile	40.16,   66.97	36.48,   49.17	64.30,   75.05		
Karnofsky index					
     N	782	592	190	<0.0001	
     Mean ± SD	83.48 ±   12.93	85.17 ±   11.88	78.21 ±   14.58		
     Median	90.00	90.00	80.00		
     Min, Max	40.00,  100.00	40.00,  100.00	40.00,  100.00		
    25, 75 Percentile	80.00,   90.00	80.00,   90.00	70.00,   90.00		
History of liver transplant*, N (%)					
     No liver transplant	605 ( 73.1%)	414 ( 67.3%)	191 ( 89.7%)	<0.0001	
     Liver transplant	223 ( 26.9%)	201 ( 32.7%)	22 ( 10.3%)		
BNP (pg/mL)					
     N	635	502	133	<0.0001	
     Mean ± SD	426.10 ± 1863.50	245.93 ±  996.17	1,106.15 ± 3510.46		
     Median	80.00	67.20	181.90		
     Min, Max	4.00,32434.00	4.60,15614.00	4.00,32434.00		
    25, 75 Percentile	37.00,  195.00	34.00,  154.50	70.70,  625.00		
NT-BNP (pg/mL)					
     N	198	114	84	0.0005	
     Mean ± SD	998.06 ± 2909.49	386.31 ± 1053.92	1,828.29 ± 4167.51		
     Median	162.50	97.00	418.60		
     Min, Max	13.00,25118.00	13.00, 8538.00	20.00,25118.00		
    25, 75 Percentile	64.00,  576.00	51.00,  259.00	148.50, 1671.00		
Troponin I (ng/mL)					
     N	5	3	2	0.2018	
     Mean ± SD	0.21 ±    0.37	0.03 ±    0.03	0.49 ±    0.53		
     Median	0.06	0.02	0.49		
     Min, Max	0.00,    0.86	0.00,    0.06	0.11,    0.86		
    25, 75 Percentile	0.02,    0.11	0.00,    0.06	0.11,    0.86		
Troponin T (ng/mL)					
     N	75	44	31	0.0008	
     Mean ± SD	0.02 ±    0.03	0.01 ±    0.01	0.04 ±    0.05		
     Median	0.01	0.01	0.02		
     Min, Max	0.00,    0.19	0.00,    0.04	0.00,    0.19		
    25, 75 Percentile	0.01,    0.03	0.00,    0.01	0.01,    0.05		
Creatinine (mg/dL)					
     N	807	606	201	0.0098	
     Mean ± SD	78.10 ±   54.65	75.24 ±   48.22	86.73 ±   70.03		
     Median	69.84	69.42	72.00		
     Min, Max	34.48,  981.24	34.48,  981.24	35.36,  763.78		
    25, 75 Percentile	59.23,   83.10	59.23,   81.33	62.76,   88.40		
Estimated GFR					
     N	804	606	198	<0.0001	
     Mean ± SD	102.91 ±   38.55	109.84 ±   38.49	81.73 ±   30.14		
     Median	102.95	109.80	83.25		
     Min, Max	0.00,  331.80	0.00,  331.80	0.00,  154.20		
    25, 75 Percentile	80.00,  126.10	87.90,  129.80	60.90,  101.20		
Modified BMI					
     N	769	584	185	0.0073	
     Mean ± SD	1,049.55 ±  242.60	1,036.36 ±  232.18	1,091.21 ±  269.37		
     Median	1030.72	1027.06	1041.84		
     Min, Max	413.82, 2001.17	413.82, 1994.55	571.09, 2001.17		
    25, 75 Percentile	884.49, 1191.16	883.18, 1169.75	912.81, 1255.01		
Left atrium (mm)					
     N	159	81	78	<0.0001	
     Mean ± SD	37.50 ±    7.25	33.97 ±    5.49	41.16 ±    7.06		
     Median	37.00	34.00	41.00		
     Min, Max	13.00,   68.00	13.00,   47.00	28.00,   68.00		
    25, 75 Percentile	33.00,   42.00	31.40,   37.00	37.00,   45.00		
LV septum (mm)					
     N	177	93	84	<0.0001	
     Mean ± SD	13.21 ±    4.57	10.79 ±    2.77	15.90 ±    4.69		
     Median	12.00	10.00	16.00		
     Min, Max	7.00,   29.00	7.00,   24.00	7.00,   29.00		
    25, 75 Percentile	10.00,   16.00	9.00,   12.00	12.00,   19.50		
LV posterior wall (mm)					
     N	166	90	76	<0.0001	
     Mean ± SD	10.82 ±    3.40	9.49 ±    1.85	12.39 ±    4.10		
     Median	10.00	9.00	12.00		
     Min, Max	4.90,   26.00	6.00,   17.00	4.90,   26.00		
    25, 75 Percentile	8.50,   12.00	8.00,   10.00	9.00,   15.00		
LV diastolic diameter (mm)					
     N	158	80	78	0.0077	
     Mean ± SD	45.90 ±    5.56	44.74 ±    5.42	47.09 ±    5.49		
     Median	46.00	45.00	47.00		
     Min, Max	28.00,   67.00	28.00,   59.00	36.00,   67.00		
    25, 75 Percentile	42.00,   50.00	41.50,   48.00	44.00,   50.80		
LV systolic diameter (mm)					
     N	141	74	67	0.2236	
     Mean ± SD	28.26 ±    5.32	27.74 ±    4.02	28.83 ±    6.45		
     Median	28.00	27.75	29.00		
     Min, Max	2.50,   41.00	18.80,   41.00	2.50,   41.00		
    25, 75 Percentile	25.00,   31.00	25.00,   30.00	24.00,   34.00		
End diastolic volume (mL)					
     N	0	0	0		
     Mean ± SD					
     Median					
     Min, Max					
    25, 75 Percentile					
End systolic volume (mL)					
     N	0	0	0		
     Mean ± SD					
     Median					
     Min, Max					
    25, 75 Percentile					
Stroke volume index					
     N	105	58	47	0.0256	
     Mean ± SD	75.92 ±   19.40	72.14 ±   18.37	80.60 ±   19.82		
     Median	75.00	72.00	80.00		
     Min, Max	30.00,  127.00	30.00,  127.00	33.00,  121.00		
    25, 75 Percentile	63.00,   89.00	58.00,   83.00	67.00,   92.00		
LV ejection fraction (%)					
     N	100	62	38	0.2616	
     Mean ± SD	60.22 ±    9.57	61.06 ±    9.53	58.84 ±    9.58		
     Median	60.00	60.00	59.50		
     Min, Max	30.00,   83.00	30.00,   83.00	40.00,   79.00		
    25, 75 Percentile	55.00,   66.50	56.00,   67.00	55.00,   66.00		
E/A ratio					
     N	68	47	21	0.1848	
     Mean ± SD	1.15 ±    0.44	1.20 ±    0.38	1.04 ±    0.55		
     Median	1.06	1.13	0.80		
     Min, Max	0.53,    2.51	0.53,    2.16	0.62,    2.51		
    25, 75 Percentile	0.83,    1.33	0.96,    1.33	0.66,    1.32		
E wave deceleration time (msec)					
     N	85	55	30	<0.0001	
     Mean ± SD	192.39 ±   66.38	170.09 ±   50.32	233.27 ±   73.33		
     Median	189.00	176.00	230.00		
     Min, Max	71.00,  434.00	71.00,  296.00	100.00,  434.00		
    25, 75 Percentile	153.00,  228.00	129.00,  201.00	176.00,  281.00		
NYHA FC, N (%)					
     I	16 (  1.9%)	5 (  0.8%)	11 (  5.2%)	<0.0001	
     II	29 (  3.5%)	7 (  1.1%)	22 ( 10.3%)		
     III	10 (  1.2%)	2 (  0.3%)	8 (  3.8%)		
     IV	1 (  0.1%)	0 (  0.0%)	1 (  0.5%)		
     Missing	772 ( 93.2%)	601 ( 97.7%)	171 ( 80.3%)		
Cardiomyopathy/Cardiac Disorder, N (%)					
     Without symptom	613 ( 74.0%)	489 ( 79.5%)	124 ( 58.2%)	<0.0001	
     With symptom	215 ( 26.0%)	126 ( 20.5%)	89 ( 41.8%)		
Neuropathy, N (%)					
     Without symptom	33 (  4.0%)	20 (  3.3%)	13 (  6.1%)	0.0668	
     With symptom	795 ( 96.0%)	595 ( 96.7%)	200 ( 93.9%)		

 * History of liver transplant includes any liver transplant recorded in the THAOS database, both pre- and post-baseline.	
  NYHA FC is entered in place of severity when subjects report heart failure as a symptom.  Subjects who do not report heart failure are missing this information.	
 Notes: Baseline lab and echo values were selected using the values closest to consent within the baseline period (consent +/- six months).  The analytic cohort includes subjects who have baseline BNP and/or NT-BNP.	
