# Supplementary material for: Impact of genotype and phenotype on cardiac biomarkers in patients with transthyretin amyloidosis – Report from the Transthyretin Amyloidosis Outcome Survey (THAOS)
Source: PLoS One. 2017 Apr 6;12(4):e0173086. doi: 10.1371/journal.pone.0173086 (PMC5383030; doi:10.1371/journal.pone.0173086)
Supplement: S1 Supporting Information — (ZIP) [file pone.0173086.s001.zip › S6_Table_Q026_Table_23_v2.sas.rtf]

 Table 23. Pearson Correlation of Log-transformed Troponin I and Troponin T with Baseline Characteristics	

Characteristic	N	Pearson
Correlation	P-value	
Log-transformed Troponin I	        .	    .   	    .   	
    Age (yrs)	      107	   0.222	   0.021	
    Modified BMI	       64	   0.079	   0.533	
    Left atrium (mm)	       40	   0.039	   0.813	
    LV septum (mm)	       52	   0.348	   0.011	
    LV posterior wall (mm)	       52	   0.434	   0.001	
    Duration of disease (yrs)	       92	  -0.258	   0.013	
Log-transformed Troponin T	        .	    .   	    .   	
    Age (yrs)	      274	   0.656	   0.000	
    Modified BMI	      223	  -0.114	   0.089	
    Left atrium (mm)	      190	   0.452	   0.000	
    LV septum (mm)	      202	   0.556	   0.000	
    LV posterior wall (mm)	      201	   0.606	   0.000	
    Duration of disease (yrs)	      232	   0.088	   0.183	

 Notes: Baseline lab and echo values were selected using the values closest to consent within the baseline period (consent +/- six months).  The analytic cohort includes subjects who have baseline BNP and/or NT-BNP.	
