# Supplementary material for: Impact of genotype and phenotype on cardiac biomarkers in patients with transthyretin amyloidosis – Report from the Transthyretin Amyloidosis Outcome Survey (THAOS)
Source: PLoS One. 2017 Apr 6;12(4):e0173086. doi: 10.1371/journal.pone.0173086 (PMC5383030; doi:10.1371/journal.pone.0173086)
Supplement: S1 Supporting Information — (ZIP) [file pone.0173086.s001.zip › S7_Table_Q027_Table_24_v2.sas.rtf]

 Table 24. Pearson Correlation of Log-transformed BNP and NT-BNP with Baseline Characteristics	

Characteristic	N	Pearson
Correlation	P-value	
Log-transformed BNP	        .	    .   	    .   	
    Age (yrs)	     1079	   0.585	   0.000	
    Modified BMI	      987	  -0.319	   0.000	
    Left atrium (mm)	      148	   0.571	   0.000	
    LV septum (mm)	      175	   0.605	   0.000	
    LV posterior wall (mm)	      168	   0.563	   0.000	
    Duration of disease (yrs)	      766	   0.308	   0.000	
Log-transformed NT-BNP	        .	    .   	    .   	
    Age (yrs)	      550	   0.698	   0.000	
    Modified BMI	      438	  -0.236	   0.000	
    Left atrium (mm)	      267	   0.337	   0.000	
    LV septum (mm)	      307	   0.654	   0.000	
    LV posterior wall (mm)	      302	   0.649	   0.000	
    Duration of disease (yrs)	      457	   0.186	   0.000	

 Notes: Baseline lab and echo values were selected using the values closest to consent within the baseline period (consent +/- six months).  The analytic cohort includes subjects who have baseline BNP and/or NT-BNP.	
