# Supplementary material for: Impact of genotype and phenotype on cardiac biomarkers in patients with transthyretin amyloidosis – Report from the Transthyretin Amyloidosis Outcome Survey (THAOS)
Source: PLoS One. 2017 Apr 6;12(4):e0173086. doi: 10.1371/journal.pone.0173086 (PMC5383030; doi:10.1371/journal.pone.0173086)
Supplement: S1 Supporting Information — (ZIP) [file pone.0173086.s001.zip › S8_Table_Q028_Table_25_univariate_results_v2.sas.rtf]

 Table 25. Univariate Mortality Risk Factors for All Subjects and for Subjects with TTR Mutation	

variable	Model 1
(All)	Model 2
(TTR Mutation)	
Age	1.08 (1.07,1.10), <.001	1.10 (1.08,1.12), <.001	
Male (vs female)	3.48 (2.24,5.39), <.001	2.75 (1.72,4.40), <.001	
Liver transplant at any time (vs. no liver tx)	0.99 (0.61,1.61), .97	1.36 (0.82,2.25), .24	
Liver transplant prior to consent (vs. no liver tx	0.67 (0.32,1.37), .27	0.86 (0.42,1.79), .69	
mBMI	1.00 (1.00,1.00), <.001	1.00 (1.00,1.00), <.001	
Duration of disease	1.04 (1.02,1.06), <.001	1.04 (1.01,1.07), .002	
eGFR	0.98 (0.97,0.98), <.001	0.98 (0.97,0.98), <.001	
Val30Met (vs non-Val30Met)		0.13 (0.09,0.21), <.001	
