# Supplementary material for: Impact of genotype and phenotype on cardiac biomarkers in patients with transthyretin amyloidosis – Report from the Transthyretin Amyloidosis Outcome Survey (THAOS)
Source: PLoS One. 2017 Apr 6;12(4):e0173086. doi: 10.1371/journal.pone.0173086 (PMC5383030; doi:10.1371/journal.pone.0173086)
Supplement: S1 Supporting Information — (ZIP) [file pone.0173086.s001.zip › S9_Table_Q038_Table_27_v2.sas.rtf]

 Table 27. Comparison of Baseline Characteristics (Clinical, Biological, Echocardiography), Without Biomarkers vs. With Biomarkers	

 	Without Biomarkers
(N = 913)	With Biomarkers
(N = 1617)	P-value
Without Biomarkers vs. With Biomarkers	
Gender, N (%)				
     Male	534 ( 58.5%)	881 ( 54.5%)	0.0513	
     Female	379 ( 41.5%)	736 ( 45.5%)		
Age1 (yrs)				
     N	913	1617	<0.0001	
     Mean ± SD	52.29 ±   17.52	48.02 ±   18.07		
     Median	50.96	43.91		
     Min, Max	18.50,   89.25	18.34,   89.57		
    25, 75 Percentile	36.82,   67.54	32.97,   64.38		
Race/Ethnicity, N (%)				
     Caucasian	534 ( 58.5%)	494 ( 30.6%)	<0.0001	
     African Descent	59 (  6.5%)	50 (  3.1%)		
     American Hispanic	17 (  1.9%)	0 (  0.0%)		
     Latino American	81 (  8.9%)	7 (  0.4%)		
     Asian	60 (  6.6%)	40 (  2.5%)		
     Other	25 (  2.7%)	11 (  0.7%)		
     Missing	137 ( 15.0%)	1,015 ( 62.8%)		
TTR genotype, N (%)				
     Wild Type	130 ( 14.2%)	165 ( 10.2%)	<0.0001	
     Val30Met	442 ( 48.4%)	1,210 ( 74.8%)		
     Non-Val30Met	341 ( 37.3%)	242 ( 15.0%)		
Age at onset of ATTR symptoms1 (yrs)				
     N	741	1309	0.0005	
     Mean ± SD	47.78 ±   17.20	45.09 ±   16.66		
     Median	46.48	41.70		
     Min, Max	9.56,   84.71	9.72,   89.45		
    25, 75 Percentile	33.08,   61.54	30.97,   59.40		
Age at measurement of BNP/NT-BNP (yrs)				
     N	0	1617		
     Mean ± SD		48.02 ±   18.07		
     Median		43.95		
     Min, Max		18.34,   89.57		
    25, 75 Percentile		32.95,   64.40		
Age at measurement of Troponin I/T (yrs)				
     N	9	367	0.5675	
     Mean ± SD	65.22 ±   11.81	62.01 ±   16.75		
     Median	71.02	67.01		
     Min, Max	44.49,   76.27	19.90,   89.57		
    25, 75 Percentile	57.48,   75.64	51.55,   74.58		
Karnofsky index				
     N	677	1451	<0.0001	
     Mean ± SD	82.79 ±   17.79	86.55 ±   14.43		
     Median	90.00	90.00		
     Min, Max	0.00,  100.00	0.00,  100.00		
    25, 75 Percentile	70.00,  100.00	80.00,  100.00		
History of liver transplant2, N (%)				
     No liver transplant	756 ( 82.8%)	1,368 ( 84.6%)	0.2369	
     Liver transplant	157 ( 17.2%)	249 ( 15.4%)		
BNP (pg/mL)				
     N	0	1079		
     Mean ± SD		376.61 ± 1628.36		
     Median		68.00		
     Min, Max		4.00,32434.00		
    25, 75 Percentile		30.50,  194.90		
NT-BNP (pg/mL)				
     N	0	550		
     Mean ± SD		4,257.80 ±18306.08		
     Median		337.90		
     Min, Max		1.00,296450.0		
    25, 75 Percentile		73.00, 2584.00		
Troponin I (ng/mL)				
     N	4	108	0.5747	
     Mean ± SD	0.07 ±    0.03	0.12 ±    0.17		
     Median	0.08	0.08		
     Min, Max	0.03,    0.10	0.00,    1.00		
    25, 75 Percentile	0.05,    0.10	0.04,    0.13		
Troponin T (ng/mL)				
     N	5	274	0.8049	
     Mean ± SD	0.05 ±    0.07	0.04 ±    0.07		
     Median	0.02	0.03		
     Min, Max	0.01,    0.17	0.00,    1.00		
    25, 75 Percentile	0.01,    0.03	0.01,    0.05		
Creatinine (mg/dL)				
     N	293	1575	0.8820	
     Mean ± SD	91.58 ±   53.49	89.97 ±  183.72		
     Median	78.68	72.49		
     Min, Max	34.48,  555.00	1.63, 6011.20		
    25, 75 Percentile	61.88,   99.89	61.00,   90.00		
Estimated GFR				
     N	292	1565	0.0165	
     Mean ± SD	87.42 ±   42.31	104.52 ±  120.36		
     Median	85.80	100.80		
     Min, Max	0.00,  257.00	0.00, 4040.30		
    25, 75 Percentile	61.10,  109.55	71.80,  126.00		
Modified BMI				
     N	202	1419	<0.0001	
     Mean ± SD	996.40 ±  262.00	1,077.67 ±  236.22		
     Median	981.06	1054.62		
     Min, Max	477.92, 1828.99	413.82, 2094.79		
    25, 75 Percentile	819.30, 1167.34	923.30, 1207.53		
Left atrium (mm)				
     N	253	408	0.0002	
     Mean ± SD	39.20 ±    9.53	42.33 ±   11.12		
     Median	38.00	41.00		
     Min, Max	3.50,   76.00	13.00,  170.00		
    25, 75 Percentile	33.00,   45.00	35.35,   47.00		
LV septum (mm)				
     N	285	474	0.0083	
     Mean ± SD	14.01 ±    9.49	15.41 ±    5.03		
     Median	13.00	16.00		
     Min, Max	1.00,  151.00	2.30,   29.00		
    25, 75 Percentile	9.90,   17.00	11.00,   19.00		
LV posterior wall (mm)				
     N	282	462	0.5732	
     Mean ± SD	13.87 ±   13.88	13.48 ±    4.31		
     Median	12.00	13.00		
     Min, Max	6.00,  177.00	2.20,   26.00		
    25, 75 Percentile	9.00,   16.00	10.00,   17.00		
LV diastolic diameter (mm)				
     N	280	451	0.7815	
     Mean ± SD	45.48 ±   25.25	45.14 ±    6.34		
     Median	45.00	45.00		
     Min, Max	5.00,  450.00	3.60,   67.00		
    25, 75 Percentile	40.00,   48.00	41.00,   49.00		
LV systolic diameter (mm)				
     N	252	407	0.8284	
     Mean ± SD	30.83 ±   19.86	31.07 ±    7.25		
     Median	29.00	30.00		
     Min, Max	16.00,  330.00	2.50,   61.00		
    25, 75 Percentile	26.00,   33.35	26.70,   35.00		
End diastolic volume (mL)				
     N	7	7	0.0558	
     Mean ± SD	72.86 ±   27.24	107.86 ±   34.21		
     Median	70.00	114.00		
     Min, Max	30.00,  101.00	46.00,  141.00		
    25, 75 Percentile	54.00,   99.00	84.00,  138.00		
End systolic volume (mL)				
     N	6	6	0.0213	
     Mean ± SD	29.67 ±   15.81	58.33 ±   20.32		
     Median	23.50	61.50		
     Min, Max	17.00,   58.00	24.00,   78.00		
    25, 75 Percentile	18.00,   38.00	51.00,   74.00		
Stroke volume index				
     N	86	191	0.0001	
     Mean ± SD	58.79 ±   18.25	69.19 ±   21.88		
     Median	56.00	69.00		
     Min, Max	23.00,   96.00	17.00,  127.00		
    25, 75 Percentile	46.00,   74.00	54.00,   84.00		
LV ejection fraction (%)				
     N	293	382	<0.0001	
     Mean ± SD	58.54 ±   13.81	50.29 ±   14.53		
     Median	61.00	55.00		
     Min, Max	5.00,   90.00	10.00,   83.00		
    25, 75 Percentile	53.00,   67.00	40.00,   60.00		
E/A ratio				
     N	108	143	0.9229	
     Mean ± SD	1.58 ±    1.38	1.60 ±    0.94		
     Median	1.23	1.33		
     Min, Max	0.10,   12.30	0.25,    4.58		
    25, 75 Percentile	0.93,    1.76	0.94,    2.00		
E wave deceleration time (msec)				
     N	112	211	0.5011	
     Mean ± SD	181.79 ±   51.12	186.06 ±   55.74		
     Median	183.00	181.00		
     Min, Max	38.00,  342.00	71.00,  434.00		
    25, 75 Percentile	141.00,  209.00	151.00,  215.00		
NYHA FC3, N (%)				
     I	32 (  3.5%)	37 (  2.3%)	0.0562	
     II	93 ( 10.2%)	146 (  9.0%)		
     III	73 (  8.0%)	120 (  7.4%)		
     IV	16 (  1.8%)	14 (  0.9%)		
     Missing	699 ( 76.6%)	1,300 ( 80.4%)		
Cardiomyopathy/Cardiac Disorder, N (%)				
     Without symptom	559 ( 61.2%)	1,110 ( 68.6%)	0.0002	
     With symptom	354 ( 38.8%)	507 ( 31.4%)		
Neuropathy, N (%)				
     Without symptom	312 ( 34.2%)	579 ( 35.8%)	0.4086	
     With symptom	601 ( 65.8%)	1,038 ( 64.2%)		

 1 The Wilcoxon Rank Sum test was used to calculate the P-values for Age and Age at onset of symptoms.	
 2 History of liver transplant includes any liver transplant recorded in the THAOS database, both pre- and post-baseline.	
 3 NYHA FC is entered in place of severity when subjects report heart failure as a symptom.  Subjects who do not report heart failure are missing this information.	
 Notes: Baseline lab and echo values were selected using the values closest to consent within the baseline period (consent +/- six months).  The analytic cohort includes subjects who have baseline BNP and/or NT-BNP.	
