# Supplementary material for: Impact of genotype and phenotype on cardiac biomarkers in patients with transthyretin amyloidosis – Report from the Transthyretin Amyloidosis Outcome Survey (THAOS)
Source: PLoS One. 2017 Apr 6;12(4):e0173086. doi: 10.1371/journal.pone.0173086 (PMC5383030; doi:10.1371/journal.pone.0173086)
Supplement: S1 Supporting Information — (ZIP) [file pone.0173086.s001.zip › S10_Table_Addl_PHREG.pdf]

Cardiac Biomarkers Analysis
Additionala Analysis to address Journal reviewers' comments
Hazard Ratio for Early vs. Late Onset

The PHREG Procedure

Model Information		
**Data Set**	CARDIAC.CARDIAC_CO	
**Dependent Variable**	yrs_followup	Time from Enrollment (Years)
**Censoring Variable**	death_fl	Patient has died
**Censoring Value(s)**	0	
**Ties Handling**	BRESLOW	

**Number of Observations Read**	1210
**Number of Observations Used**	828

Class Level Information		
**Class**	**Value**	**Design Variables**
**onset**	**Early**	1
	**Late**	0

Summary of the Number of Event and Censored Values			
**Total**	**Event**	**Censored**	**Percent Censored**
828	42	786	94.93

Convergence Status
Convergence criterion (GCONV=1E-8) satisfied.

Model Fit Statistics		
**Criterion**	**Without Covariates**	**With Covariates**
**-2 LOG L**	481.567	454.859
**AIC**	481.567	456.859
**SBC**	481.567	458.597

Cardiac Biomarkers Analysis
Additionala Analysis to address Journal reviewers' comments
Hazard Ratio for Early vs. Late Onset

The PHREG Procedure

Testing Global Null Hypothesis: BETA=0			
**Test**	**Chi-Square**	**DF**	**Pr > ChiSq**
**Likelihood Ratio**	26.7082	1	<.0001
**Score**	35.6452	1	<.0001
**Wald**	28.4656	1	<.0001

Type 3 Tests			
**Effect**	**DF**	**Wald Chi-Square**	**Pr > ChiSq**
**onset**	1	28.4656	<.0001

Analysis of Maximum Likelihood Estimates								
**Parameter**		**DF**	**Parameter Estimate**	**Standard Error**	**Chi-Square**	**Pr > ChiSq**	**Hazard Ratio**	**Label**
**onset**	**Early**	1	-1.68592	0.31599	28.4656	<.0001	0.185	Early = Age LT 50, Late = Age GE 50 Early

Hazard Ratios for onset			
**Description**	**Point Estimate**	**95% Wald Confidence Limits**	
**onset Early vs Late**	0.185	0.100	0.344

Cardiac Biomarkers Analysis
Additionala Analysis to address Journal reviewers' comments
Hazard Ratio for Val122i (vs Non-Val122i) among Non-Val30Met subjects

The PHREG Procedure

Model Information		
**Data Set**	WORK.VAL122I	
**Dependent Variable**	yrs_followup	Time from Enrollment (Years)
**Censoring Variable**	death_fl	Patient has died
**Censoring Value(s)**	0	
**Ties Handling**	BRESLOW	

**Number of Observations Read**	1452
**Number of Observations Used**	242

Summary of the Number of Event and Censored Values			
**Total**	**Event**	**Censored**	**Percent Censored**
242	40	202	83.47

Convergence Status
Convergence criterion (GCONV=1E-8) satisfied.

Model Fit Statistics		
**Criterion**	**Without Covariates**	**With Covariates**
**-2 LOG L**	351.381	340.549
**AIC**	351.381	342.549
**SBC**	351.381	344.238

Testing Global Null Hypothesis: BETA=0			
**Test**	**Chi-Square**	**DF**	**Pr > ChiSq**
**Likelihood Ratio**	10.8321	1	0.0010
**Score**	12.8818	1	0.0003
**Wald**	11.6998	1	0.0006

Cardiac Biomarkers Analysis
Additionala Analysis to address Journal reviewers' comments
Hazard Ratio for Val122i (vs Non-Val122i) among Non-Val30Met subjects

The PHREG Procedure

Analysis of Maximum Likelihood Estimates						
Parameter	DF	Parameter Estimate	Standard Error	Chi-Square	Pr > ChiSq	Hazard Ratio
**V122i**	1	1.09667	0.32062	11.6998	0.0006	2.994

Hazard Ratios for V122i			
Description	Point Estimate	95% Wald Confidence Limits	
**V122i Unit=1**	2.994	1.597	5.613
