# Supplementary material for: Impact of genotype and phenotype on cardiac biomarkers in patients with transthyretin amyloidosis – Report from the Transthyretin Amyloidosis Outcome Survey (THAOS)
Source: PLoS One. 2017 Apr 6;12(4):e0173086. doi: 10.1371/journal.pone.0173086 (PMC5383030; doi:10.1371/journal.pone.0173086)
Supplement: S1 Supporting Information — (ZIP) [file pone.0173086.s001.zip › S13_Table_C_O'Q_Optimal Cutpoint_BNP.pdf]

The SAS System

OPTIMAL DICHOTOMIZATION OF CONTINUOUS VARIABLES

EXPLORATION OF CUTPOINT FOR BNP_CD_BL IN EXPLANING DEATH_FL

RANGES FOR BNP_CD_BL

			Percentile							
**Patient Subset**	**N**	**Min**	**5**	**10**	**25**	**Med**	**75**	**90**	**95**	**Max**
All	1079	4	10.5	15.7	30.5	68	194.9	689	1170	32434

OPTIMAL DICHOTOMIZATION OF CONTINUOUS VARIABLES

Cut-Points		Contal and O'Quigley Method				
**Cut Level**	**BNP_CD_BL**	**SK**	**Absolute SK**	**Q Statistic**	**P-value**	**Selected Cut-Point**
1	4	0	0	0	0.3000	
2	4.6	0	0	0	0.3000	
3	5	0.0740323	0.0740323	0.0092409	0.3000	
4	5.1	1.1970814	1.1970814	0.1494235	0.3000	
5	5.3	1.2246571	1.2246571	0.1528656	0.3000	
6	5.5	1.2246571	1.2246571	0.1528656	0.3000	
7	5.6	1.2915334	1.2915334	0.1612133	0.3000	
8	5.7	1.3606027	1.3606027	0.1698348	0.3000	

Cut-Points		Contal and O'Quigley Method				
**Cut Level**	**BNP_CD_BL**	**SK**	**Absolute SK**	**Q Statistic**	**P-value**	**Selected Cut-Point**
9	5.8	1.4296719	1.4296719	0.1784562	0.3000	
10	6	1.4296719	1.4296719	0.1784562	0.3000	
11	6.1	1.571306	1.571306	0.1961354	0.3000	
12	6.5	1.5946153	1.5946153	0.199045	0.3000	
13	7	1.6192945	1.6192945	0.2021255	0.3000	
14	7.1	2.2023942	2.2023942	0.2749098	0.3000	
15	7.5	2.2692705	2.2692705	0.2832576	0.3000	
16	7.7	2.4191013	2.4191013	0.3019599	0.3000	
17	7.9	2.577704	2.577704	0.3217572	0.3000	
18	8	2.577704	2.577704	0.3217572	0.3000	
19	8.7	2.8242205	2.8242205	0.3525282	0.3000	
20	9	2.8517962	2.8517962	0.3559703	0.3000	

Cut-Points		Contal and O'Quigley Method				
**Cut Level**	**BNP_CD_BL**	**SK**	**Absolute SK**	**Q Statistic**	**P-value**	**Selected Cut-Point**
21	9.9	2.948482	2.948482	0.3680389	0.3000	
22	10	3.0225143	3.0225143	0.3772798	0.3000	
23	10.1	2.1520563	2.1520563	0.2686265	0.3000	
24	10.3	2.2049587	2.2049587	0.27523	0.3000	
25	10.4	2.2340337	2.2340337	0.2788592	0.3000	
26	10.5	2.3926364	2.3926364	0.2986565	0.3000	
27	10.7	2.5788148	2.5788148	0.3218959	0.3000	
28	10.8	2.603494	2.603494	0.3249764	0.3000	
29	11	2.6268034	2.6268034	0.327886	0.3000	
30	11.5	3.4520625	3.4520625	0.4308974	0.3000	
31	11.6	3.4767417	3.4767417	0.433978	0.3000	
32	11.8	3.500051	3.500051	0.4368875	0.3000	

Cut-Points		Contal and O'Quigley Method				
**Cut Level**	**BNP_CD_BL**	**SK**	**Absolute SK**	**Q Statistic**	**P-value**	**Selected Cut-Point**
33	12	3.6860608	3.6860608	0.4601058	0.3000	
34	12.2	3.6907684	3.6907684	0.4606934	0.3000	
35	12.4	3.9260756	3.9260756	0.4900652	0.3000	
36	12.5	3.9756449	3.9756449	0.4962526	0.3000	
37	12.7	4.0383875	4.0383875	0.5040844	0.3000	
38	12.8	4.0659633	4.0659633	0.5075265	0.3000	
39	13	4.0659633	4.0659633	0.5075265	0.3000	
40	13.1	4.1064728	4.1064728	0.512583	0.3000	
41	13.2	4.1486602	4.1486602	0.517849	0.3000	
42	13.7	4.2015626	4.2015626	0.5244524	0.3000	
43	13.9	4.4054406	4.4054406	0.5499011	0.3000	
44	14	4.4909297	4.4909297	0.5605721	0.3000	

**Cut-Points**		**Contal and O'Quigley Method**				
**Cut Level**	**BNP_CD_BL**	**SK**	**Absolute SK**	**Q Statistic**	**P-value**	**Selected Cut-Point**
45	14.1	4.6786782	4.6786782	0.5840075	0.3000	
46	14.3	4.7077532	4.7077532	0.5876367	0.3000	
47	14.4	4.8401697	4.8401697	0.6041654	0.3000	
48	14.7	4.8401697	4.8401697	0.6041654	0.3000	
49	14.8	4.8401697	4.8401697	0.6041654	0.3000	
50	15	4.8401697	4.8401697	0.6041654	0.3000	
51	15.1	4.9720416	4.9720416	0.620626	0.3000	
52	15.2	5.0411109	5.0411109	0.6292475	0.3000	
53	15.3	5.1997136	5.1997136	0.6490448	0.3000	
54	15.5	5.1997136	5.1997136	0.6490448	0.3000	
55	15.6	5.3844364	5.3844364	0.6721025	0.3000	
56	15.7	5.4135114	5.4135114	0.6757317	0.3000	

Cut-Points		Contal and O'Quigley Method				
**Cut Level**	**BNP_CD_BL**	**SK**	**Absolute SK**	**Q Statistic**	**P-value**	**Selected Cut-Point**
57	15.8	5.4490181	5.4490181	0.6801638	0.3000	
58	15.9	5.5204798	5.5204798	0.6890838	0.3000	
59	16	5.589549	5.589549	0.6977053	0.3000	
60	16.1	5.957321	5.957321	0.7436117	0.3000	
61	16.3	6.0858436	6.0858436	0.7596544	0.3000	
62	16.4	6.2444463	6.2444463	0.7794516	0.3000	
63	16.6	6.2444463	6.2444463	0.7794516	0.3000	
64	17	6.2444463	6.2444463	0.7794516	0.3000	
65	17.1	6.648156	6.648156	0.829844	0.3000	
66	17.3	6.8067588	6.8067588	0.8496413	0.3000	
67	17.5	6.8596611	6.8596611	0.8562447	0.3000	
68	17.8	6.879115	6.879115	0.858673	0.3000	

Cut-Points		Contal and O'Quigley Method				
**Cut Level**	**BNP_CD_BL**	**SK**	**Absolute SK**	**Q Statistic**	**P-value**	**Selected Cut-Point**
69	17.9	6.9481842	6.9481842	0.8672944	0.3000	
70	18	6.9903716	6.9903716	0.8725604	0.3000	
71	18.2	7.2807395	7.2807395	0.9088051	0.3000	
72	18.3	7.3860691	7.3860691	0.9219526	0.3000	
73	18.4	7.3860691	7.3860691	0.9219526	0.3000	
74	18.5	7.4151441	7.4151441	0.9255819	0.3000	
75	18.9	7.4151441	7.4151441	0.9255819	0.3000	
76	19	7.5088447	7.5088447	0.9372779	0.3000	
77	19.1	8.1355345	8.1355345	1.0155033	0.2543	
78	19.2	8.2941372	8.2941372	1.0353006	0.2344	
79	19.3	8.3398125	8.3398125	1.0410019	0.2290	
80	19.4	8.6201278	8.6201278	1.0759918	0.1974	

Cut-Points		Contal and O'Quigley Method				
**Cut Level**	**BNP_CD_BL**	**SK**	**Absolute SK**	**Q Statistic**	**P-value**	**Selected Cut-Point**
81	19.5	8.6421213	8.6421213	1.0787371	0.1951	
82	19.8	8.6641149	8.6641149	1.0814824	0.1928	
83	19.9	8.6641149	8.6641149	1.0814824	0.1928	
84	20	8.6641149	8.6641149	1.0814824	0.1928	
85	20.1	9.0951161	9.0951161	1.1352813	0.1519	
86	20.3	9.2273085	9.2273085	1.151782	0.1409	
87	20.5	9.2628152	9.2628152	1.156214	0.1380	
88	20.9	9.2628152	9.2628152	1.156214	0.1380	
89	21	9.3157176	9.3157176	1.1628175	0.1338	
90	21.1	10.043952	10.043952	1.253718	0.0863	
91	21.3	10.140638	10.140638	1.2657866	0.0812	
92	21.5	10.294226	10.294226	1.284958	0.0736	

Cut-Points		Contal and O'Quigley Method				
**Cut Level**	**BNP_CD_BL**	**SK**	**Absolute SK**	**Q Statistic**	**P-value**	**Selected Cut-Point**
93	21.7	10.294226	10.294226	1.284958	0.0736	
94	21.8	10.390911	10.390911	1.2970266	0.0692	
95	21.9	10.418487	10.418487	1.3004687	0.0679	
96	22	10.473313	10.473313	1.3073122	0.0655	
97	22.1	10.569998	10.569998	1.3193808	0.0615	
98	22.3	10.662377	10.662377	1.3309118	0.0579	
99	22.5	10.82098	10.82098	1.3507091	0.0520	
100	22.6	10.82098	10.82098	1.3507091	0.0520	
101	22.8	10.979582	10.979582	1.3705064	0.0467	
102	22.9	11.259402	11.259402	1.4054343	0.0385	
103	23	11.51469	11.51469	1.4373002	0.0321	
104	23.1	11.643213	11.643213	1.4533429	0.0293	

**Cut-Points**		**Contal and O'Quigley Method**				
**Cut Level**	**BNP_CD_BL**	**SK**	**Absolute SK**	**Q Statistic**	**P-value**	**Selected Cut-Point**
105	23.2	11.677083	11.677083	1.4575706	0.0286	
106	23.4	11.677083	11.677083	1.4575706	0.0286	
107	23.6	11.677083	11.677083	1.4575706	0.0286	
108	23.7	11.704659	11.704659	1.4610127	0.0280	
109	23.8	11.773728	11.773728	1.4696342	0.0266	
110	24	12.029016	12.029016	1.5015001	0.0220	
111	24.2	12.356561	12.356561	1.5423852	0.0172	
112	24.3	12.515163	12.515163	1.5621825	0.0152	
113	24.5	12.515163	12.515163	1.5621825	0.0152	
114	24.6	12.515163	12.515163	1.5621825	0.0152	
115	24.7	12.907257	12.907257	1.6111249	0.0111	
116	25	13.003943	13.003943	1.6231935	0.0103	

Cut-Points		Contal and O'Quigley Method				
**Cut Level**	**BNP_CD_BL**	**SK**	**Absolute SK**	**Q Statistic**	**P-value**	**Selected Cut-Point**
117	25.3	12.3862	12.3862	1.5460848	0.0168	
118	25.6	12.662274	12.662274	1.5805453	0.0135	
119	25.8	12.691349	12.691349	1.5841745	0.0132	
120	25.9	12.733536	12.733536	1.5894404	0.0128	
121	26	12.770704	12.770704	1.5940799	0.0124	
122	26.2	13.120434	13.120434	1.6377343	0.0094	
123	26.3	13.310287	13.310287	1.6614323	0.0080	
124	26.4	13.310287	13.310287	1.6614323	0.0080	
125	26.6	13.381748	13.381748	1.6703524	0.0075	
126	26.8	13.406427	13.406427	1.6734329	0.0074	
127	26.9	13.46917	13.46917	1.6812646	0.0070	
128	27	13.493849	13.493849	1.6843452	0.0069	

Cut-Points		Contal and O'Quigley Method				
**Cut Level**	**BNP_CD_BL**	**SK**	**Absolute SK**	**Q Statistic**	**P-value**	**Selected Cut-Point**
129	27.1	13.522924	13.522924	1.6879744	0.0067	
130	27.2	13.544918	13.544918	1.6907197	0.0066	
131	27.3	13.807801	13.807801	1.7235336	0.0053	
132	27.4	13.807801	13.807801	1.7235336	0.0053	
133	27.7	13.83822	13.83822	1.7273306	0.0051	
134	27.8	13.83822	13.83822	1.7273306	0.0051	
135	28	13.947327	13.947327	1.7409497	0.0047	
136	28.5	14.172536	14.172536	1.769061	0.0038	
137	28.6	14.172536	14.172536	1.769061	0.0038	
138	28.8	14.172536	14.172536	1.769061	0.0038	
139	28.9	14.249172	14.249172	1.778627	0.0036	
140	29.1	14.472546	14.472546	1.8065092	0.0029	

Cut-Points		Contal and O'Quigley Method				
**Cut Level**	**BNP_CD_BL**	**SK**	**Absolute SK**	**Q Statistic**	**P-value**	**Selected Cut-Point**
141	29.2	14.472546	14.472546	1.8065092	0.0029	
142	29.4	14.497225	14.497225	1.8095897	0.0029	
143	29.5	14.625748	14.625748	1.8256323	0.0025	
144	29.6	14.650427	14.650427	1.8287129	0.0025	
145	29.9	14.80903	14.80903	1.8485102	0.0022	
146	30	14.80903	14.80903	1.8485102	0.0022	
147	30.2	14.932233	14.932233	1.8638888	0.0019	
148	30.5	15.047801	15.047801	1.8783144	0.0017	
149	30.6	15.075377	15.075377	1.8817565	0.0017	
150	30.8	15.075377	15.075377	1.8817565	0.0017	
151	30.9	15.146839	15.146839	1.8906765	0.0016	
152	31	15.174414	15.174414	1.8941186	0.0015	

Cut-Points		Contal and O'Quigley Method				
**Cut Level**	**BNP_CD_BL**	**SK**	**Absolute SK**	**Q Statistic**	**P-value**	**Selected Cut-Point**
153	31.4	15.377222	15.377222	1.9194337	0.0013	
154	31.7	15.41439	15.41439	1.9240732	0.0012	
155	31.8	15.499275	15.499275	1.9346688	0.0011	
156	31.9	15.52835	15.52835	1.9382981	0.0011	
157	32	15.55303	15.55303	1.9413786	0.0011	
158	32.2	15.847087	15.847087	1.9780838	0.0008	
159	32.8	15.98596	15.98596	1.9954183	0.0007	
160	33	16.082646	16.082646	2.007487	0.0006	
161	33.2	16.65304	16.65304	2.0786854	0.0004	
162	33.6	16.676349	16.676349	2.0815949	0.0003	
163	33.7	16.676349	16.676349	2.0815949	0.0003	
164	34	16.690786	16.690786	2.0833969	0.0003	

Cut-Points		Contal and O'Quigley Method				
**Cut Level**	**BNP_CD_BL**	**SK**	**Absolute SK**	**Q Statistic**	**P-value**	**Selected Cut-Point**
165	34.1	17.420949	17.420949	2.1745383	0.0002	
166	34.2	17.442943	17.442943	2.1772836	0.0002	
167	34.3	17.472018	17.472018	2.1809128	0.0001	
168	34.4	17.472018	17.472018	2.1809128	0.0001	
169	34.8	17.472018	17.472018	2.1809128	0.0001	
170	34.9	17.501093	17.501093	2.1845421	0.0001	
171	35	17.567969	17.567969	2.1928898	0.0001	
172	35.1	18.47289	18.47289	2.3058449	<.0001	
173	35.5	18.525793	18.525793	2.3124484	<.0001	
174	35.9	18.594862	18.594862	2.3210698	<.0001	
175	36	18.700574	18.700574	2.334265	<.0001	
176	36.3	19.129291	19.129291	2.3877789	<.0001	

Cut-Points		Contal and O'Quigley Method				
**Cut Level**	**BNP_CD_BL**	**SK**	**Absolute SK**	**Q Statistic**	**P-value**	**Selected Cut-Point**
177	36.4	19.1732	19.1732	2.3932597	<.0001	
178	36.5	19.237971	19.237971	2.4013447	<.0001	
179	36.9	19.37241	19.37241	2.4181259	<.0001	
180	37	19.44148	19.44148	2.4267473	<.0001	
181	37.5	20.086267	20.086267	2.5072317	<.0001	
182	37.7	20.086267	20.086267	2.5072317	<.0001	
183	37.8	20.182952	20.182952	2.5193003	<.0001	
184	37.9	20.207631	20.207631	2.5223808	<.0001	
185	38	20.25154	20.25154	2.5278616	<.0001	
186	38.2	20.276219	20.276219	2.5309422	<.0001	
187	38.6	20.408636	20.408636	2.5474708	<.0001	
188	38.7	20.408636	20.408636	2.5474708	<.0001	

Cut-Points		Contal and O'Quigley Method				
**Cut Level**	**BNP_CD_BL**	**SK**	**Absolute SK**	**Q Statistic**	**P-value**	**Selected Cut-Point**
189	38.9	20.430629	20.430629	2.5502161	<.0001	
190	39	20.527315	20.527315	2.5622847	<.0001	
191	39.1	21.249068	21.249068	2.6523762	<.0001	
192	39.3	21.315934	21.315934	2.6607227	<.0001	
193	39.4	21.368836	21.368836	2.6673261	<.0001	
194	39.8	21.555015	21.555015	2.6905655	<.0001	
195	39.9	21.578324	21.578324	2.693475	<.0001	
196	40	21.578324	21.578324	2.693475	<.0001	
197	40.2	21.607399	21.607399	2.6971043	<.0001	
198	40.3	21.681432	21.681432	2.7063452	<.0001	
199	40.4	21.72534	21.72534	2.711826	<.0001	
200	40.7	21.822026	21.822026	2.7238946	<.0001	

Cut-Points		Contal and O'Quigley Method				
**Cut Level**	**BNP_CD_BL**	**SK**	**Absolute SK**	**Q Statistic**	**P-value**	**Selected Cut-Point**
201	41	20.842744	20.842744	2.6016576	<.0001	
202	41.1	20.911813	20.911813	2.610279	<.0001	
203	41.2	21.101666	21.101666	2.633977	<.0001	
204	41.3	21.183686	21.183686	2.6442151	<.0001	
205	41.5	21.219193	21.219193	2.6486471	<.0001	
206	41.9	21.26138	21.26138	2.6539131	<.0001	
207	42	21.26138	21.26138	2.6539131	<.0001	
208	42.2	21.721759	21.721759	2.711379	<.0001	
209	42.3	21.782501	21.782501	2.7189611	<.0001	
210	42.4	21.804495	21.804495	2.7217064	<.0001	
211	42.5	21.822688	21.822688	2.7239773	<.0001	
212	42.6	21.887459	21.887459	2.7320622	<.0001	

Cut-Points		Contal and O'Quigley Method				
**Cut Level**	**BNP_CD_BL**	**SK**	**Absolute SK**	**Q Statistic**	**P-value**	**Selected Cut-Point**
213	42.9	22.057289	22.057289	2.7532609	<.0001	
214	43	22.181653	22.181653	2.7687844	<.0001	
215	43.2	22.428169	22.428169	2.7995554	<.0001	
216	43.3	22.494264	22.494264	2.8078056	<.0001	
217	43.5	22.507457	22.507457	2.8094523	<.0001	
218	43.6	22.773946	22.773946	2.8427164	<.0001	
219	43.8	22.773946	22.773946	2.8427164	<.0001	
220	44	22.855966	22.855966	2.8529544	<.0001	
221	44.1	23.040689	23.040689	2.8760121	<.0001	
222	44.2	23.109759	23.109759	2.8846335	<.0001	
223	44.3	23.194644	23.194644	2.8952292	<.0001	
224	45	23.228514	23.228514	2.899457	<.0001	

Cut-Points		Contal and O'Quigley Method				
**Cut Level**	**BNP_CD_BL**	**SK**	**Absolute SK**	**Q Statistic**	**P-value**	**Selected Cut-Point**
225	45.2	23.503378	23.503378	2.9337664	<.0001	
226	45.4	23.738686	23.738686	2.9631382	<.0001	
227	46	24.085104	24.085104	3.0063793	<.0001	
228	46.2	24.599323	24.599323	3.0705658	<.0001	
229	46.4	24.736169	24.736169	3.0876473	<.0001	
230	46.5	24.736169	24.736169	3.0876473	<.0001	
231	46.7	24.736169	24.736169	3.0876473	<.0001	
232	46.9	24.760848	24.760848	3.0907278	<.0001	
233	47	24.857534	24.857534	3.1027965	<.0001	
234	47.3	24.926603	24.926603	3.1114179	<.0001	
235	47.5	24.951283	24.951283	3.1144984	<.0001	
236	47.8	24.974592	24.974592	3.117408	<.0001	

**Cut-Points**		**Contal and O'Quigley Method**				
**Cut Level**	**BNP_CD_BL**	**SK**	**Absolute SK**	**Q Statistic**	**P-value**	**Selected Cut-Point**
237	48	25.002168	25.002168	3.1208501	<.0001	
238	48.1	25.341367	25.341367	3.16319	<.0001	
239	48.6	25.36336	25.36336	3.1659353	<.0001	
240	49	25.521963	25.521963	3.1857326	<.0001	
241	49.2	25.755462	25.755462	3.2148786	<.0001	
242	49.4	25.816204	25.816204	3.2224607	<.0001	
243	50	25.974807	25.974807	3.242258	<.0001	
244	50.3	26.295592	26.295592	3.2822994	<.0001	
245	50.6	26.295592	26.295592	3.2822994	<.0001	
246	50.8	26.318901	26.318901	3.285209	<.0001	
247	50.9	26.318901	26.318901	3.285209	<.0001	
248	51	26.398212	26.398212	3.2951087	<.0001	

Cut-Points		Contal and O'Quigley Method				
**Cut Level**	**BNP_CD_BL**	**SK**	**Absolute SK**	**Q Statistic**	**P-value**	**Selected Cut-Point**
249	51.3	26.696166	26.696166	3.3323004	<.0001	
250	51.7	26.750992	26.750992	3.3391439	<.0001	
251	51.8	26.780067	26.780067	3.3427731	<.0001	
252	51.9	26.832969	26.832969	3.3493765	<.0001	
253	52	26.832969	26.832969	3.3493765	<.0001	
254	52.1	27.291809	27.291809	3.4066503	<.0001	
255	52.3	27.291809	27.291809	3.4066503	<.0001	
256	52.4	27.35658	27.35658	3.4147353	<.0001	
257	52.8	27.381259	27.381259	3.4178158	<.0001	
258	52.9	27.410334	27.410334	3.421445	<.0001	
259	53.7	27.665622	27.665622	3.4533109	<.0001	
260	54	27.762308	27.762308	3.4653796	<.0001	

Cut-Points		Contal and O'Quigley Method				
**Cut Level**	**BNP_CD_BL**	**SK**	**Absolute SK**	**Q Statistic**	**P-value**	**Selected Cut-Point**
261	54.3	27.920911	27.920911	3.4851769	<.0001	
262	54.4	28.044114	28.044114	3.5005555	<.0001	
263	54.5	28.113183	28.113183	3.509177	<.0001	
264	54.6	28.175926	28.175926	3.5170087	<.0001	
265	54.7	28.244995	28.244995	3.5256301	<.0001	
266	55	28.299821	28.299821	3.5324736	<.0001	
267	55.2	28.428344	28.428344	3.5485162	<.0001	
268	55.5	28.450337	28.450337	3.5512615	<.0001	
269	55.7	28.475016	28.475016	3.5543421	<.0001	
270	56	28.609456	28.609456	3.5711233	<.0001	
271	56.2	29.008682	29.008682	3.6209559	<.0001	
272	56.3	29.105368	29.105368	3.6330245	<.0001	

**Cut-Points**		**Contal and O'Quigley Method**				
**Cut Level**	**BNP_CD_BL**	**SK**	**Absolute SK**	**Q Statistic**	**P-value**	**Selected Cut-Point**
273	56.5	29.128677	29.128677	3.6359341	<.0001	
274	56.6	29.263117	29.263117	3.6527153	<.0001	
275	56.9	29.567493	29.567493	3.6907085	<.0001	
276	57	29.632264	29.632264	3.6987934	<.0001	
277	57.3	29.790867	29.790867	3.7185907	<.0001	
278	57.4	29.816987	29.816987	3.7218511	<.0001	
279	57.5	29.886056	29.886056	3.7304726	<.0001	
280	57.7	29.886056	29.886056	3.7304726	<.0001	
281	57.8	29.889575	29.889575	3.7309118	<.0001	
282	58	29.889575	29.889575	3.7309118	<.0001	
283	58.3	30.012778	30.012778	3.7462904	<.0001	
284	58.7	30.012778	30.012778	3.7462904	<.0001	

Cut-Points		Contal and O'Quigley Method				
**Cut Level**	**BNP_CD_BL**	**SK**	**Absolute SK**	**Q Statistic**	**P-value**	**Selected Cut-Point**
285	59	30.079655	30.079655	3.7546381	<.0001	
286	59.1	30.214094	30.214094	3.7714193	<.0001	
287	59.8	30.278865	30.278865	3.7795043	<.0001	
288	59.9	30.30794	30.30794	3.7831335	<.0001	
289	60	30.402711	30.402711	3.7949631	<.0001	
290	60.6	30.799634	30.799634	3.8445082	<.0001	
291	61	30.822943	30.822943	3.8474178	<.0001	
292	61.2	30.902254	30.902254	3.8573175	<.0001	
293	61.3	31.060856	31.060856	3.8771148	<.0001	
294	61.4	31.127733	31.127733	3.8854625	<.0001	
295	61.5	31.158392	31.158392	3.8892896	<.0001	
296	62	31.258121	31.258121	3.9017381	<.0001	

Cut-Points		Contal and O'Quigley Method				
**Cut Level**	**BNP_CD_BL**	**SK**	**Absolute SK**	**Q Statistic**	**P-value**	**Selected Cut-Point**
297	62.1	31.371774	31.371774	3.9159246	<.0001	
298	62.2	31.371774	31.371774	3.9159246	<.0001	
299	62.4	31.397894	31.397894	3.919185	<.0001	
300	62.7	31.556497	31.556497	3.9389823	<.0001	
301	62.9	31.585572	31.585572	3.9426115	<.0001	
302	63	30.601255	30.601255	3.819746	<.0001	
303	63.1	30.766355	30.766355	3.8403542	<.0001	
304	63.9	31.112773	31.112773	3.8835953	<.0001	
305	64	31.137452	31.137452	3.8866758	<.0001	
306	64.3	31.137452	31.137452	3.8866758	<.0001	
307	64.5	31.200195	31.200195	3.8945075	<.0001	
308	64.8	31.368342	31.368342	3.9154962	<.0001	

Cut-Points		Contal and O'Quigley Method				
**Cut Level**	**BNP_CD_BL**	**SK**	**Absolute SK**	**Q Statistic**	**P-value**	**Selected Cut-Point**
309	64.9	31.412251	31.412251	3.920977	<.0001	
310	65.3	31.533616	31.533616	3.9361262	<.0001	
311	66	31.756989	31.756989	3.9640084	<.0001	
312	66.2	31.915592	31.915592	3.9838057	<.0001	
313	66.4	31.987054	31.987054	3.9927258	<.0001	
314	66.5	32.16576	32.16576	4.0150324	<.0001	
315	66.6	32.16576	32.16576	4.0150324	<.0001	
316	66.7	32.190439	32.190439	4.0181129	<.0001	
317	67	32.190439	32.190439	4.0181129	<.0001	
318	67.7	32.356994	32.356994	4.0389029	<.0001	
319	67.9	31.381673	31.381673	3.9171602	<.0001	
320	68	31.690107	31.690107	3.9556599	<.0001	

Cut-Points		Contal and O'Quigley Method				
Cut Level	BNP_CD_BL	SK	Absolute SK	Q Statistic	P-value	Selected Cut-Point
321	68.1	30.822371	30.822371	3.8473464	<.0001	
322	68.3	30.851446	30.851446	3.8509756	<.0001	
323	68.9	30.851446	30.851446	3.8509756	<.0001	
324	69	30.879022	30.879022	3.8544177	<.0001	
325	69.1	31.076394	31.076394	3.8790543	<.0001	
326	69.7	31.145463	31.145463	3.8876757	<.0001	
327	69.8	31.242149	31.242149	3.8997443	<.0001	
328	70	31.264142	31.264142	3.9024896	<.0001	
329	70.7	31.360828	31.360828	3.9145582	<.0001	
330	71	31.563339	31.563339	3.9398363	<.0001	
331	71.6	31.94715	31.94715	3.9877449	<.0001	
332	71.9	31.94715	31.94715	3.9877449	<.0001	

Cut-Points		Contal and O'Quigley Method				
**Cut Level**	**BNP_CD_BL**	**SK**	**Absolute SK**	**Q Statistic**	**P-value**	**Selected Cut-Point**
333	72	31.976225	31.976225	3.9913741	<.0001	
334	72.4	32.287922	32.287922	4.0302811	<.0001	
335	72.5	32.4009	32.4009	4.0443833	<.0001	
336	73	32.4009	32.4009	4.0443833	<.0001	
337	73.1	32.559503	32.559503	4.0641806	<.0001	
338	73.6	32.60177	32.60177	4.0694566	<.0001	
339	73.9	32.683791	32.683791	4.0796947	<.0001	
340	74	32.683791	32.683791	4.0796947	<.0001	
341	74.8	33.000996	33.000996	4.1192892	<.0001	
342	75	33.097682	33.097682	4.1313579	<.0001	
343	75.6	33.097682	33.097682	4.1313579	<.0001	
344	75.8	33.169143	33.169143	4.1402779	<.0001	

Cut-Points		Contal and O'Quigley Method				
**Cut Level**	**BNP_CD_BL**	**SK**	**Absolute SK**	**Q Statistic**	**P-value**	**Selected Cut-Point**
345	75.9	33.184827	33.184827	4.1422356	<.0001	
346	76	33.210947	33.210947	4.145496	<.0001	
347	76.2	33.352581	33.352581	4.1631752	<.0001	
348	76.4	33.485679	33.485679	4.1797889	<.0001	
349	76.7	33.514754	33.514754	4.1834181	<.0001	
350	77	33.750061	33.750061	4.2127899	<.0001	
351	77.5	33.750061	33.750061	4.2127899	<.0001	
352	78.3	33.750061	33.750061	4.2127899	<.0001	
353	78.6	33.846747	33.846747	4.2248585	<.0001	
354	79	33.846747	33.846747	4.2248585	<.0001	
355	79.2	34.066091	34.066091	4.2522378	<.0001	
356	80	34.132967	34.132967	4.2605855	<.0001	

Cut-Points		Contal and O'Quigley Method				
**Cut Level**	**BNP_CD_BL**	**SK**	**Absolute SK**	**Q Statistic**	**P-value**	**Selected Cut-Point**
357	80.2	34.890707	34.890707	4.355169	<.0001	
358	80.6	34.962169	34.962169	4.3640891	<.0001	
359	80.7	34.965687	34.965687	4.3645283	<.0001	
360	81	35.050573	35.050573	4.375124	<.0001	
361	81.2	35.265584	35.265584	4.4019623	<.0001	
362	81.3	35.288893	35.288893	4.4048719	<.0001	
363	81.8	35.478746	35.478746	4.4285699	<.0001	
364	82	35.558056	35.558056	4.4384697	<.0001	
365	83	34.601965	34.601965	4.3191273	<.0001	
366	83.6	34.736405	34.736405	4.3359085	<.0001	
367	84	34.805474	34.805474	4.3445299	<.0001	
368	84.1	34.914581	34.914581	4.358149	<.0001	

Cut-Points		Contal and O'Quigley Method				
Cut Level	BNP_CD_BL	SK	Absolute SK	Q Statistic	P-value	Selected Cut-Point
369	85	34.937891	34.937891	4.3610586	<.0001	
370	85.1	35.096493	35.096493	4.3808559	<.0001	
371	85.9	35.178514	35.178514	4.3910939	<.0001	
372	86	35.199232	35.199232	4.39368	<.0001	
373	86.1	35.292932	35.292932	4.405376	<.0001	
374	87	35.374953	35.374953	4.4156141	<.0001	
375	87.3	35.471638	35.471638	4.4276827	<.0001	
376	87.5	35.630241	35.630241	4.44748	<.0001	
377	87.7	34.644677	34.644677	4.3244588	<.0001	
378	88	34.741363	34.741363	4.3365274	<.0001	
379	88.1	34.859689	34.859689	4.3512972	<.0001	
380	88.3	35.049541	35.049541	4.3749952	<.0001	

Cut-Points		Contal and O'Quigley Method				
**Cut Level**	**BNP_CD_BL**	**SK**	**Absolute SK**	**Q Statistic**	**P-value**	**Selected Cut-Point**
381	88.7	35.0989	35.0989	4.3811563	<.0001	
382	89	35.0989	35.0989	4.3811563	<.0001	
383	89.3	35.23334	35.23334	4.3979375	<.0001	
384	90	35.300216	35.300216	4.4062852	<.0001	
385	90.1	35.396902	35.396902	4.4183538	<.0001	
386	90.7	35.425977	35.425977	4.421983	<.0001	
387	90.8	35.468164	35.468164	4.427249	<.0001	
388	90.9	34.498824	34.498824	4.3062529	<.0001	
389	91	34.498824	34.498824	4.3062529	<.0001	
390	92	34.657426	34.657426	4.3260502	<.0001	
391	92.7	34.754112	34.754112	4.3381188	<.0001	
392	94	34.755276	34.755276	4.3382641	<.0001	

Cut-Points		Contal and O'Quigley Method				
**Cut Level**	**BNP_CD_BL**	**SK**	**Absolute SK**	**Q Statistic**	**P-value**	**Selected Cut-Point**
393	94.4	34.989038	34.989038	4.3674429	<.0001	
394	94.8	35.112241	35.112241	4.3828216	<.0001	
395	96	35.115759	35.115759	4.3832608	<.0001	
396	96.1	35.212445	35.212445	4.3953294	<.0001	
397	97	35.212445	35.212445	4.3953294	<.0001	
398	97.4	35.354079	35.354079	4.4130086	<.0001	
399	98	35.358787	35.358787	4.4135962	<.0001	
400	98.4	34.394294	34.394294	4.2932051	<.0001	
401	98.7	34.544124	34.544124	4.3119074	<.0001	
402	99	34.564842	34.564842	4.3144935	<.0001	
403	99.6	34.661528	34.661528	4.3265621	<.0001	
404	100	34.686207	34.686207	4.3296427	<.0001	

Cut-Points		Contal and O'Quigley Method				
**Cut Level**	**BNP_CD_BL**	**SK**	**Absolute SK**	**Q Statistic**	**P-value**	**Selected Cut-Point**
405	100.2	34.865807	34.865807	4.3520609	<.0001	
406	100.6	34.962493	34.962493	4.3641296	<.0001	
407	100.7	34.962493	34.962493	4.3641296	<.0001	
408	100.8	34.962493	34.962493	4.3641296	<.0001	
409	101.2	34.962493	34.962493	4.3641296	<.0001	
410	101.3	35.059179	35.059179	4.3761982	<.0001	
411	101.7	35.104854	35.104854	4.3818995	<.0001	
412	102	35.148763	35.148763	4.3873803	<.0001	
413	102.7	35.307366	35.307366	4.4071776	<.0001	
414	103.3	35.378827	35.378827	4.4160977	<.0001	
415	104	35.403506	35.403506	4.4191782	<.0001	
416	104.1	35.403506	35.403506	4.4191782	<.0001	

Cut-Points		Contal and O'Quigley Method				
**Cut Level**	**BNP_CD_BL**	**SK**	**Absolute SK**	**Q Statistic**	**P-value**	**Selected Cut-Point**
417	105	35.470383	35.470383	4.4275259	<.0001	
418	105.1	35.91537	35.91537	4.4830707	<.0001	
419	105.3	35.970196	35.970196	4.4899142	<.0001	
420	105.9	35.970196	35.970196	4.4899142	<.0001	
421	106	35.970196	35.970196	4.4899142	<.0001	
422	106.9	36.062574	36.062574	4.5014452	<.0001	
423	108	36.129451	36.129451	4.5097929	<.0001	
424	108.9	36.312732	36.312732	4.5326707	<.0001	
425	109	36.334726	36.334726	4.535416	<.0001	
426	109.7	36.362302	36.362302	4.5388581	<.0001	
427	110.5	36.458987	36.458987	4.5509267	<.0001	
428	110.8	36.458987	36.458987	4.5509267	<.0001	

Cut-Points		Contal and O'Quigley Method				
**Cut Level**	**BNP_CD_BL**	**SK**	**Absolute SK**	**Q Statistic**	**P-value**	**Selected Cut-Point**
429	111	36.488062	36.488062	4.554556	<.0001	
430	111.9	36.72337	36.72337	4.5839278	<.0001	
431	112	36.72337	36.72337	4.5839278	<.0001	
432	112.6	36.976199	36.976199	4.6154867	<.0001	
433	113	37.110639	37.110639	4.632268	<.0001	
434	113.7	37.233842	37.233842	4.6476466	<.0001	
435	114	37.302912	37.302912	4.6562681	<.0001	
436	115	37.529431	37.529431	4.684543	<.0001	
437	115.8	37.598501	37.598501	4.6931644	<.0001	
438	116	37.598501	37.598501	4.6931644	<.0001	
439	116.7	37.707608	37.707608	4.7067835	<.0001	
440	117	37.735184	37.735184	4.7102256	<.0001	

Cut-Points		Contal and O'Quigley Method				
**Cut Level**	**BNP_CD_BL**	**SK**	**Absolute SK**	**Q Statistic**	**P-value**	**Selected Cut-Point**
441	117.6	37.764259	37.764259	4.7138548	<.0001	
442	117.7	37.840895	37.840895	4.7234208	<.0001	
443	118.4	37.845603	37.845603	4.7240084	<.0001	
444	119	37.870282	37.870282	4.727089	<.0001	
445	119.7	38.163324	38.163324	4.7636675	<.0001	
446	119.8	38.230201	38.230201	4.7720152	<.0001	
447	120	38.230201	38.230201	4.7720152	<.0001	
448	120.6	38.297077	38.297077	4.7803629	<.0001	
449	121	38.379097	38.379097	4.7906009	<.0001	
450	121.4	38.5377	38.5377	4.8103982	<.0001	
451	121.6	38.585167	38.585167	4.8163233	<.0001	
452	122.8	38.609847	38.609847	4.8194038	<.0001	

Cut-Points		Contal and O'Quigley Method				
**Cut Level**	**BNP_CD_BL**	**SK**	**Absolute SK**	**Q Statistic**	**P-value**	**Selected Cut-Point**
453	123	38.678916	38.678916	4.8280253	<.0001	
454	123.6	38.775602	38.775602	4.8400939	<.0001	
455	123.9	38.823069	38.823069	4.8460189	<.0001	
456	124	38.823069	38.823069	4.8460189	<.0001	
457	124.7	39.171524	39.171524	4.8895142	<.0001	
458	125	39.171524	39.171524	4.8895142	<.0001	
459	127	39.177431	39.177431	4.8902515	<.0001	
460	128.1	39.177431	39.177431	4.8902515	<.0001	
461	128.4	39.177431	39.177431	4.8902515	<.0001	
462	129.1	39.193114	39.193114	4.8922092	<.0001	
463	130	39.235302	39.235302	4.8974751	<.0001	
464	130.6	39.235302	39.235302	4.8974751	<.0001	

Cut-Points		Contal and O'Quigley Method				
**Cut Level**	**BNP_CD_BL**	**SK**	**Absolute SK**	**Q Statistic**	**P-value**	**Selected Cut-Point**
465	130.7	39.270808	39.270808	4.9019072	<.0001	
466	132.3	39.270808	39.270808	4.9019072	<.0001	
467	133	39.429411	39.429411	4.9217045	<.0001	
468	133.3	39.682176	39.682176	4.9532554	<.0001	
469	133.8	39.749053	39.749053	4.9616031	<.0001	
470	135	39.811795	39.811795	4.9694349	<.0001	
471	135.9	39.866621	39.866621	4.9762784	<.0001	
472	136.1	39.988592	39.988592	4.9915033	<.0001	
473	137.4	40.057662	40.057662	5.0001247	<.0001	
474	137.6	40.129123	40.129123	5.0090448	<.0001	
475	138	40.129123	40.129123	5.0090448	<.0001	
476	141	40.263563	40.263563	5.025826	<.0001	

Cut-Points		Contal and O'Quigley Method				
**Cut Level**	**BNP_CD_BL**	**SK**	**Absolute SK**	**Q Statistic**	**P-value**	**Selected Cut-Point**
477	142.7	40.316466	40.316466	5.0324294	<.0001	
478	143.4	40.316466	40.316466	5.0324294	<.0001	
479	144.6	40.329658	40.329658	5.0340762	<.0001	<====
480	145	39.371846	39.371846	4.914519	<.0001	
481	146	39.395155	39.395155	4.9174285	<.0001	
482	147	39.553758	39.553758	4.9372258	<.0001	
483	147.1	38.809005	38.809005	4.8442635	<.0001	
484	147.4	38.875882	38.875882	4.8526112	<.0001	
485	147.7	38.899191	38.899191	4.8555207	<.0001	
486	148	39.112619	39.112619	4.8821615	<.0001	
487	150.5	39.112619	39.112619	4.8821615	<.0001	
488	150.7	39.112619	39.112619	4.8821615	<.0001	

Cut-Points		Contal and O'Quigley Method				
**Cut Level**	**BNP_CD_BL**	**SK**	**Absolute SK**	**Q Statistic**	**P-value**	**Selected Cut-Point**
489	152.1	39.112619	39.112619	4.8821615	<.0001	
490	152.2	39.154807	39.154807	4.8874275	<.0001	
491	153	39.1768	39.1768	4.8901728	<.0001	
492	153.6	39.241571	39.241571	4.8982577	<.0001	
493	154	39.318208	39.318208	4.9078237	<.0001	
494	154.5	38.459842	38.459842	4.8006797	<.0001	
495	155.3	38.493712	38.493712	4.8049075	<.0001	
496	156	38.535899	38.535899	4.8101735	<.0001	
497	157	38.632585	38.632585	4.8222421	<.0001	
498	157.6	38.632585	38.632585	4.8222421	<.0001	
499	158	38.632585	38.632585	4.8222421	<.0001	
500	159.7	37.681854	37.681854	4.7035688	<.0001	

Cut-Points		Contal and O'Quigley Method				
**Cut Level**	**BNP_CD_BL**	**SK**	**Absolute SK**	**Q Statistic**	**P-value**	**Selected Cut-Point**
501	159.9	37.681854	37.681854	4.7035688	<.0001	
502	162	37.681854	37.681854	4.7035688	<.0001	
503	163	37.80018	37.80018	4.7183386	<.0001	
504	163.8	36.823489	36.823489	4.596425	<.0001	
505	164	36.852564	36.852564	4.6000542	<.0001	
506	165	36.856082	36.856082	4.6004934	<.0001	
507	165.1	36.938103	36.938103	4.6107314	<.0001	
508	166.3	36.938103	36.938103	4.6107314	<.0001	
509	168.2	36.938103	36.938103	4.6107314	<.0001	
510	168.3	36.961412	36.961412	4.613641	<.0001	
511	169.8	36.990487	36.990487	4.6172702	<.0001	
512	170	37.091024	37.091024	4.6298195	<.0001	

Cut-Points		Contal and O'Quigley Method				
**Cut Level**	**BNP_CD_BL**	**SK**	**Absolute SK**	**Q Statistic**	**P-value**	**Selected Cut-Point**
513	170.1	36.214227	36.214227	4.520375	<.0001	
514	170.2	36.214227	36.214227	4.520375	<.0001	
515	171.2	36.310913	36.310913	4.5324436	<.0001	
516	171.6	36.377789	36.377789	4.5407913	<.0001	
517	172.3	36.408449	36.408449	4.5446184	<.0001	
518	173.6	36.531652	36.531652	4.559997	<.0001	
519	174	36.565522	36.565522	4.5642248	<.0001	
520	175.4	36.80083	36.80083	4.5935966	<.0001	
521	176	36.959432	36.959432	4.6133939	<.0001	
522	176.9	37.149285	37.149285	4.6370919	<.0001	
523	179	37.285518	37.285518	4.6540969	<.0001	
524	181.9	37.419958	37.419958	4.6708781	<.0001	

Cut-Points		Contal and O'Quigley Method				
**Cut Level**	**BNP_CD_BL**	**SK**	**Absolute SK**	**Q Statistic**	**P-value**	**Selected Cut-Point**
525	182.2	37.463866	37.463866	4.6763589	<.0001	
526	183.8	37.463866	37.463866	4.6763589	<.0001	
527	184	37.497736	37.497736	4.6805867	<.0001	
528	186	37.62094	37.62094	4.6959653	<.0001	
529	186.3	37.717625	37.717625	4.7080339	<.0001	
530	187	37.7467	37.7467	4.7116632	<.0001	
531	187.3	37.78057	37.78057	4.7158909	<.0001	
532	188	37.809645	37.809645	4.7195202	<.0001	
533	189	37.881107	37.881107	4.7284402	<.0001	
534	191	38.004311	38.004311	4.7438189	<.0001	
535	191.3	38.031886	38.031886	4.747261	<.0001	
536	193.2	38.128572	38.128572	4.7593296	<.0001	

Cut-Points		Contal and O'Quigley Method				
**Cut Level**	**BNP_CD_BL**	**SK**	**Absolute SK**	**Q Statistic**	**P-value**	**Selected Cut-Point**
537	193.3	38.128572	38.128572	4.7593296	<.0001	
538	194.3	38.162442	38.162442	4.7635574	<.0001	
539	194.8	38.162442	38.162442	4.7635574	<.0001	
540	194.9	38.263128	38.263128	4.7761253	<.0001	
541	195	38.285121	38.285121	4.7788706	<.0001	
542	196.6	38.369874	38.369874	4.7894497	<.0001	
543	198	38.422776	38.422776	4.7960531	<.0001	
544	201	38.519462	38.519462	4.8081217	<.0001	
545	202	37.62785	37.62785	4.6968279	<.0001	
546	202.4	37.65253	37.65253	4.6999085	<.0001	
547	203	37.677209	37.677209	4.702989	<.0001	
548	203.4	37.773894	37.773894	4.7150576	<.0001	

Cut-Points		Contal and O'Quigley Method				
**Cut Level**	**BNP_CD_BL**	**SK**	**Absolute SK**	**Q Statistic**	**P-value**	**Selected Cut-Point**
549	204	37.773894	37.773894	4.7150576	<.0001	
550	205.5	37.902417	37.902417	4.7311002	<.0001	
551	206.9	37.438688	37.438688	4.6732161	<.0001	
552	207	37.515325	37.515325	4.6827821	<.0001	
553	208	37.643847	37.643847	4.6988247	<.0001	
554	210	37.80245	37.80245	4.718622	<.0001	
555	214	36.896151	36.896151	4.6054948	<.0001	
556	219.7	36.967612	36.967612	4.6144149	<.0001	
557	222	37.039074	37.039074	4.623335	<.0001	
558	222.1	37.13576	37.13576	4.6354036	<.0001	
559	223	37.164835	37.164835	4.6390328	<.0001	
560	223.7	37.323437	37.323437	4.6588301	<.0001	

Cut-Points		Contal and O'Quigley Method				
**Cut Level**	**BNP_CD_BL**	**SK**	**Absolute SK**	**Q Statistic**	**P-value**	**Selected Cut-Point**
561	224.1	37.37634	37.37634	4.6654336	<.0001	
562	224.5	37.445409	37.445409	4.674055	<.0001	
563	226	37.452519	37.452519	4.6749425	<.0001	
564	227.5	37.459629	37.459629	4.67583	<.0001	
565	228	37.459629	37.459629	4.67583	<.0001	
566	228.2	37.60946	37.60946	4.6945323	<.0001	
567	229	37.610624	37.610624	4.6946777	<.0001	
568	229.7	37.610624	37.610624	4.6946777	<.0001	
569	230	37.610624	37.610624	4.6946777	<.0001	
570	231	37.639699	37.639699	4.6983069	<.0001	
571	233	37.683607	37.683607	4.7037877	<.0001	
572	234	38.030026	38.030026	4.7470287	<.0001	

Cut-Points		Contal and O'Quigley Method				
**Cut Level**	**BNP_CD_BL**	**SK**	**Absolute SK**	**Q Statistic**	**P-value**	**Selected Cut-Point**
573	235.3	38.130711	38.130711	4.7595966	<.0001	
574	237	38.199781	38.199781	4.7682181	<.0001	
575	240.2	38.252683	38.252683	4.7748215	<.0001	
576	243	38.252683	38.252683	4.7748215	<.0001	
577	243.3	38.394317	38.394317	4.7925007	<.0001	
578	247	38.439992	38.439992	4.7982021	<.0001	
579	250	38.544862	38.544862	4.8112923	<.0001	
580	251	38.703465	38.703465	4.8310896	<.0001	
581	254.5	38.837905	38.837905	4.8478708	<.0001	
582	258.7	38.851098	38.851098	4.8495175	<.0001	
583	259	38.851098	38.851098	4.8495175	<.0001	
584	260	38.947783	38.947783	4.8615861	<.0001	

Cut-Points		Contal and O'Quigley Method				
**Cut Level**	**BNP_CD_BL**	**SK**	**Absolute SK**	**Q Statistic**	**P-value**	**Selected Cut-Point**
585	260.4	39.106386	39.106386	4.8813834	<.0001	
586	270.1	39.129695	39.129695	4.884293	<.0001	
587	270.3	39.151689	39.151689	4.8870383	<.0001	
588	271	39.152853	39.152853	4.8871836	<.0001	
589	271.7	39.159963	39.159963	4.8880711	<.0001	
590	275.2	39.183272	39.183272	4.8909806	<.0001	
591	276	39.183272	39.183272	4.8909806	<.0001	
592	284	39.183272	39.183272	4.8909806	<.0001	
593	284.1	39.250149	39.250149	4.8993283	<.0001	
594	286.4	39.279224	39.279224	4.9029576	<.0001	
595	287.3	39.348293	39.348293	4.911579	<.0001	
596	290.9	39.424929	39.424929	4.921145	<.0001	

Cut-Points		Contal and O'Quigley Method				
**Cut Level**	**BNP_CD_BL**	**SK**	**Absolute SK**	**Q Statistic**	**P-value**	**Selected Cut-Point**
597	291.1	39.467117	39.467117	4.926411	<.0001	
598	295	39.467117	39.467117	4.926411	<.0001	
599	296.1	39.536186	39.536186	4.9350324	<.0001	
600	298.7	39.536186	39.536186	4.9350324	<.0001	
601	307.2	39.591011	39.591011	4.9418759	<.0001	
602	317	39.624881	39.624881	4.9461037	<.0001	
603	319	39.624881	39.624881	4.9461037	<.0001	
604	320	39.759321	39.759321	4.9628849	<.0001	
605	320.2	39.856007	39.856007	4.9749535	<.0001	
606	322.5	39.930039	39.930039	4.9841945	<.0001	
607	323.2	40.276458	40.276458	5.0274355	<.0001	
608	324	40.277622	40.277622	5.0275808	<.0001	

Cut-Points		Contal and O'Quigley Method				
**Cut Level**	**BNP_CD_BL**	**SK**	**Absolute SK**	**Q Statistic**	**P-value**	**Selected Cut-Point**
609	328	39.362507	39.362507	4.9133533	<.0001	
610	332	39.460586	39.460586	4.9255958	<.0001	
611	333	39.473779	39.473779	4.9272425	<.0001	
612	333.9	39.478486	39.478486	4.9278302	<.0001	
613	340	39.509146	39.509146	4.9316572	<.0001	
614	342.6	39.667749	39.667749	4.9514545	<.0001	
615	344.2	39.73252	39.73252	4.9595394	<.0001	
616	354	39.73252	39.73252	4.9595394	<.0001	
617	354.2	39.891122	39.891122	4.9793367	<.0001	
618	354.8	39.914432	39.914432	4.9822463	<.0001	
619	356.6	39.983501	39.983501	4.9908677	<.0001	
620	358	40.036403	40.036403	4.9974712	<.0001	

Cut-Points		Contal and O'Quigley Method				
**Cut Level**	**BNP_CD_BL**	**SK**	**Absolute SK**	**Q Statistic**	**P-value**	**Selected Cut-Point**
621	362.5	40.036403	40.036403	4.9974712	<.0001	
622	364.3	40.089306	40.089306	5.0040746	<.0001	
623	365.7	40.111299	40.111299	5.0068199	<.0001	
624	366	40.132017	40.132017	5.009406	<.0001	
625	368.4	39.20605	39.20605	4.8938238	<.0001	
626	371	39.315157	39.315157	4.9074429	<.0001	
627	386.3	39.322267	39.322267	4.9083303	<.0001	
628	393.3	39.322267	39.322267	4.9083303	<.0001	
629	394	39.371536	39.371536	4.9144803	<.0001	
630	395.5	39.371536	39.371536	4.9144803	<.0001	
631	397	38.378646	38.378646	4.7905446	<.0001	
632	397.2	38.537249	38.537249	4.8103419	<.0001	

Cut-Points		Contal and O'Quigley Method				
**Cut Level**	**BNP_CD_BL**	**SK**	**Absolute SK**	**Q Statistic**	**P-value**	**Selected Cut-Point**
633	401	37.571119	37.571119	4.6897465	<.0001	
634	404.9	36.729721	36.729721	4.5847206	<.0001	
635	405	35.757297	35.757297	4.4633395	<.0001	
636	405.5	35.853983	35.853983	4.4754081	<.0001	
637	410.4	35.867175	35.867175	4.4770549	<.0001	
638	412.2	35.943812	35.943812	4.4866209	<.0001	
639	413.6	35.943812	35.943812	4.4866209	<.0001	
640	414.3	36.040498	36.040498	4.4986895	<.0001	
641	416.1	36.084406	36.084406	4.5041703	<.0001	
642	418.6	36.243009	36.243009	4.5239676	<.0001	
643	422.7	36.243009	36.243009	4.5239676	<.0001	
644	423.5	36.243009	36.243009	4.5239676	<.0001	

Cut-Points		Contal and O'Quigley Method				
**Cut Level**	**BNP_CD_BL**	**SK**	**Absolute SK**	**Q Statistic**	**P-value**	**Selected Cut-Point**
645	439.2	35.259945	35.259945	4.4012585	<.0001	
646	440	35.259945	35.259945	4.4012585	<.0001	
647	443.4	34.378271	34.378271	4.2912051	<.0001	
648	444	34.412141	34.412141	4.2954328	<.0001	
649	444.1	34.412141	34.412141	4.2954328	<.0001	
650	450	34.43682	34.43682	4.2985134	<.0001	
651	451	34.43682	34.43682	4.2985134	<.0001	
652	453	34.450013	34.450013	4.3001601	<.0001	
653	454	34.450013	34.450013	4.3001601	<.0001	
654	455	33.495688	33.495688	4.1810383	<.0001	
655	457	33.495688	33.495688	4.1810383	<.0001	
656	460.6	33.54859	33.54859	4.1876417	<.0001	

Cut-Points		Contal and O'Quigley Method				
**Cut Level**	**BNP_CD_BL**	**SK**	**Absolute SK**	**Q Statistic**	**P-value**	**Selected Cut-Point**
657	466	33.615467	33.615467	4.1959895	<.0001	
658	470	32.716153	32.716153	4.0837342	<.0001	
659	477	32.716153	32.716153	4.0837342	<.0001	
660	483	32.776895	32.776895	4.0913163	<.0001	
661	484.6	32.776895	32.776895	4.0913163	<.0001	
662	493	32.795088	32.795088	4.0935872	<.0001	
663	496	32.895774	32.895774	4.1061551	<.0001	
664	497.7	33.242192	33.242192	4.1493961	<.0001	
665	502	33.355845	33.355845	4.1635826	<.0001	
666	504	32.440357	32.440357	4.0493085	<.0001	
667	514.7	32.463667	32.463667	4.0522181	<.0001	
668	530.3	32.494326	32.494326	4.0560451	<.0001	

Cut-Points		Contal and O'Quigley Method				
**Cut Level**	**BNP_CD_BL**	**SK**	**Absolute SK**	**Q Statistic**	**P-value**	**Selected Cut-Point**
669	534.7	32.494326	32.494326	4.0560451	<.0001	
670	542	32.576347	32.576347	4.0662832	<.0001	
671	545	32.618534	32.618534	4.0715491	<.0001	
672	551.9	31.689996	31.689996	3.955646	<.0001	
673	558	31.689996	31.689996	3.955646	<.0001	
674	559	30.803648	30.803648	3.8450093	<.0001	
675	571	30.805987	30.805987	3.8453013	<.0001	
676	583.4	30.85889	30.85889	3.8519047	<.0001	
677	591	30.85889	30.85889	3.8519047	<.0001	
678	593	29.919632	29.919632	3.7346637	<.0001	
679	604	30.078235	30.078235	3.7544609	<.0001	
680	605	30.147304	30.147304	3.7630824	<.0001	

Cut-Points		Contal and O'Quigley Method				
**Cut Level**	**BNP_CD_BL**	**SK**	**Absolute SK**	**Q Statistic**	**P-value**	**Selected Cut-Point**
681	615.6	30.147304	30.147304	3.7630824	<.0001	
682	619	30.200207	30.200207	3.7696858	<.0001	
683	624	30.358809	30.358809	3.7894831	<.0001	
684	625	30.362328	30.362328	3.7899223	<.0001	
685	627	30.463014	30.463014	3.8024902	<.0001	
686	634	29.550823	29.550823	3.6886277	<.0001	
687	642	29.647509	29.647509	3.7006963	<.0001	
688	645	29.647509	29.647509	3.7006963	<.0001	
689	652.8	28.710252	28.710252	3.5837049	<.0001	
690	658.1	27.732245	27.732245	3.461627	<.0001	
691	661	27.755555	27.755555	3.4645366	<.0001	
692	689	27.768747	27.768747	3.4661833	<.0001	

Cut-Points		Contal and O'Quigley Method				
**Cut Level**	**BNP_CD_BL**	**SK**	**Absolute SK**	**Q Statistic**	**P-value**	**Selected Cut-Point**
693	690	27.833518	27.833518	3.4742683	<.0001	
694	693.4	26.912829	26.912829	3.3593448	<.0001	
695	694	26.941904	26.941904	3.3629741	<.0001	
696	698.2	27.038589	27.038589	3.3750427	<.0001	
697	698.4	27.385008	27.385008	3.4182837	<.0001	
698	704	27.51353	27.51353	3.4343263	<.0001	
699	718	27.51353	27.51353	3.4343263	<.0001	
700	728	27.51353	27.51353	3.4343263	<.0001	
701	733.9	26.604281	26.604281	3.320831	<.0001	
702	748	26.605445	26.605445	3.3209763	<.0001	
703	751.2	26.795298	26.795298	3.3446743	<.0001	
704	754.3	26.141716	26.141716	3.2630921	<.0001	

Cut-Points		Contal and O'Quigley Method				
**Cut Level**	**BNP_CD_BL**	**SK**	**Absolute SK**	**Q Statistic**	**P-value**	**Selected Cut-Point**
705	758.3	26.146424	26.146424	3.2636798	<.0001	
706	760	26.24311	26.24311	3.2757484	<.0001	
707	773	26.312179	26.312179	3.2843698	<.0001	
708	785	25.365081	25.365081	3.1661501	<.0001	
709	790	25.38976	25.38976	3.1692306	<.0001	
710	796.4	24.418835	24.418835	3.0480367	<.0001	
711	803.5	24.418835	24.418835	3.0480367	<.0001	
712	809.2	24.418835	24.418835	3.0480367	<.0001	
713	810	24.418835	24.418835	3.0480367	<.0001	
714	814.1	24.471738	24.471738	3.0546401	<.0001	
715	820	24.568423	24.568423	3.0667087	<.0001	
716	834.2	23.619501	23.619501	2.9482612	<.0001	

**Cut-Points**		**Contal and O'Quigley Method**				
**Cut Level**	**BNP_CD_BL**	**SK**	**Absolute SK**	**Q Statistic**	**P-value**	**Selected Cut-Point**
717	845.5	23.647077	23.647077	2.9517033	<.0001	
718	845.9	23.709819	23.709819	2.959535	<.0001	
719	858.9	23.755495	23.755495	2.9652364	<.0001	
720	861	23.755495	23.755495	2.9652364	<.0001	
721	862	22.787757	22.787757	2.8444403	<.0001	
722	870	22.91628	22.91628	2.8604829	<.0001	
723	884	22.938273	22.938273	2.8632282	<.0001	
724	886	21.995067	21.995067	2.7454942	<.0001	
725	891	22.118271	22.118271	2.7608729	<.0001	
726	895	22.18734	22.18734	2.7694943	<.0001	
727	906	21.21346	21.21346	2.6479315	<.0001	
728	930	21.238139	21.238139	2.6510121	<.0001	

**Cut-Points**		**Contal and O'Quigley Method**				
**Cut Level**	**BNP_CD_BL**	**SK**	**Absolute SK**	**Q Statistic**	**P-value**	**Selected Cut-Point**
729	945.8	21.238139	21.238139	2.6510121	<.0001	
730	947.8	21.282048	21.282048	2.6564929	<.0001	
731	948	21.284387	21.284387	2.6567849	<.0001	
732	961	20.41291	20.41291	2.5480043	<.0001	
733	965	20.41291	20.41291	2.5480043	<.0001	
734	978.9	20.554544	20.554544	2.5656835	<.0001	
735	1013	20.596731	20.596731	2.5709495	<.0001	
736	1027	20.596731	20.596731	2.5709495	<.0001	
737	1030.1	19.633899	19.633899	2.4507657	<.0001	
738	1054.6	18.642223	18.642223	2.3269815	<.0001	
739	1064.8	17.652976	17.652976	2.2035006	0.0001	
740	1099.7	17.705879	17.705879	2.2101041	0.0001	

Cut-Points		Contal and O'Quigley Method				
**Cut Level**	**BNP_CD_BL**	**SK**	**Absolute SK**	**Q Statistic**	**P-value**	**Selected Cut-Point**
741	1101	17.719071	17.719071	2.2117508	0.0001	
742	1149	17.719071	17.719071	2.2117508	0.0001	
743	1155	17.790533	17.790533	2.2206709	0.0001	
744	1168	17.855304	17.855304	2.2287558	<.0001	
745	1170	17.855304	17.855304	2.2287558	<.0001	
746	1185	17.94019	17.94019	2.2393515	<.0001	
747	1188	16.959643	16.959643	2.1169566	0.0003	
748	1200	16.068751	16.068751	2.0057525	0.0006	
749	1313.4	16.121653	16.121653	2.012356	0.0006	
750	1313.9	16.121653	16.121653	2.012356	0.0006	
751	1317.4	16.128763	16.128763	2.0132435	0.0006	
752	1322	15.183588	15.183588	1.8952638	0.0015	

Cut-Points		Contal and O'Quigley Method				
**Cut Level**	**BNP_CD_BL**	**SK**	**Absolute SK**	**Q Statistic**	**P-value**	**Selected Cut-Point**
753	1387	15.260225	15.260225	1.9048298	0.0014	
754	1407	15.2893	15.2893	1.908459	0.0014	
755	1411	14.294007	14.294007	1.7842235	0.0034	
756	1464	14.394693	14.394693	1.7967914	0.0031	
757	1599	14.428563	14.428563	1.8010191	0.0030	
758	1601	14.43447	14.43447	1.8017564	0.0030	
759	1630	14.43447	14.43447	1.8017564	0.0030	
760	1655.3	13.511106	13.511106	1.6864992	0.0068	
761	1657	12.513445	12.513445	1.561968	0.0152	
762	1692	11.572212	11.572212	1.4444803	0.0308	
763	1713	11.61612	11.61612	1.4499611	0.0298	
764	1874	11.61612	11.61612	1.4499611	0.0298	

Cut-Points		Contal and O'Quigley Method				
**Cut Level**	**BNP_CD_BL**	**SK**	**Absolute SK**	**Q Statistic**	**P-value**	**Selected Cut-Point**
765	1894	11.61612	11.61612	1.4499611	0.0298	
766	1933	11.669023	11.669023	1.4565645	0.0287	
767	1934.8	11.740484	11.740484	1.4654846	0.0273	
768	2000	11.761202	11.761202	1.4680707	0.0269	
769	2177	11.761202	11.761202	1.4680707	0.0269	
770	2302	11.805111	11.805111	1.4735515	0.0260	
771	2404.1	10.871987	10.871987	1.357076	0.0503	
772	2490.9	9.8778938	9.8778938	1.2329901	0.0956	
773	2790	9.8778938	9.8778938	1.2329901	0.0956	
774	3014	8.9745795	8.9745795	1.1202356	0.1626	
775	3047	8.9965731	8.9965731	1.1229809	0.1606	
776	3164	8.0370826	8.0370826	1.0032142	0.2672	

Cut-Points		Contal and O'Quigley Method				
**Cut Level**	**BNP_CD_BL**	**SK**	**Absolute SK**	**Q Statistic**	**P-value**	**Selected Cut-Point**
777	3189	8.091908	8.091908	1.0100577	0.2599	
778	3517	8.091908	8.091908	1.0100577	0.2599	
779	4116	8.1274148	8.1274148	1.0144898	0.2553	
780	4377	8.1274148	8.1274148	1.0144898	0.2553	
781	4870	7.1309332	7.1309332	0.8901058	0.3000	
782	5039	7.1309332	7.1309332	0.8901058	0.3000	
783	5047	7.1978095	7.1978095	0.8984535	0.3000	
784	5447	7.1989737	7.1989737	0.8985988	0.3000	
785	5916	6.2121664	6.2121664	0.7754224	0.3000	
786	6169	6.2133305	6.2133305	0.7755677	0.3000	
787	7603	6.2133305	6.2133305	0.7755677	0.3000	
788	7665	5.2240842	5.2240842	0.6520868	0.3000	

Cut-Points		Contal and O'Quigley Method				
**Cut Level**	**BNP_CD_BL**	**SK**	**Absolute SK**	**Q Statistic**	**P-value**	**Selected Cut-Point**
789	9139	4.373915	4.373915	0.545966	0.3000	
790	9587	3.3858867	3.3858867	0.4226371	0.3000	
791	11999	3.4825724	3.4825724	0.4347058	0.3000	
792	13212	3.4825724	3.4825724	0.4347058	0.3000	
793	13254	2.7178796	2.7178796	0.3392544	0.3000	
794	15614	1.8523195	1.8523195	0.2312124	0.3000	
795	22400	0.8705122	0.8705122	0.1086601	0.3000	
796	32434	0.9525326	0.9525326	0.1188981	0.3000	
