# Supplementary material for: Impact of genotype and phenotype on cardiac biomarkers in patients with transthyretin amyloidosis – Report from the Transthyretin Amyloidosis Outcome Survey (THAOS)
Source: PLoS One. 2017 Apr 6;12(4):e0173086. doi: 10.1371/journal.pone.0173086 (PMC5383030; doi:10.1371/journal.pone.0173086)
Supplement: S1 Supporting Information — (ZIP) [file pone.0173086.s001.zip › S14_Table_C_O'Q_Optimal Cutpoint_NT-proBNP.pdf]

The SAS System

OPTIMAL DICHOTOMIZATION OF CONTINUOUS VARIABLES

EXPLORATION OF CUTPOINT FOR NTBNP_CD_BL IN EXPLANING DEATH_FL

RANGES FOR NTBNP_CD_BL

		**Percentile**								
**Patient Subset**	**N**	**Min**	**5**	**10**	**25**	**Med**	**75**	**90**	**95**	**Max**
All	550	1	21	32	73	337.9	2584	7269.39	17722	296450

The SAS System

OPTIMAL DICHOTOMIZATION OF CONTINUOUS VARIABLES

Cut-Points		Contal and O'Quigley Method				
**Cut Level**	**NTBNP_CD_BL**	**SK**	**Absolute SK**	**Q Statistic**	**P-value**	**Selected Cut-Point**
1	1	0	0	0	0.3000	
2	9	0.0227765	0.0227765	0.0036182	0.3000	
3	13	0.0227765	0.0227765	0.0036182	0.3000	
4	14	0.045553	0.045553	0.0072364	0.3000	
5	15	0.045553	0.045553	0.0072364	0.3000	
6	16.94	0.2146368	0.2146368	0.0340963	0.3000	
7	17	0.5365871	0.5365871	0.0852399	0.3000	
8	19	0.6535319	0.6535319	0.1038173	0.3000	

Cut-Points		Contal and O'Quigley Method				
**Cut Level**	**NTBNP_CD_BL**	**SK**	**Absolute SK**	**Q Statistic**	**P-value**	**Selected Cut-Point**
9	20	0.6535319	0.6535319	0.1038173	0.3000	
10	21	1.4074209	1.4074209	0.2235769	0.3000	
11	22	1.5891629	1.5891629	0.2524476	0.3000	
12	23	1.9278383	1.9278383	0.3062482	0.3000	
13	24	2.2232239	2.2232239	0.3531719	0.3000	
14	25	2.4358008	2.4358008	0.3869409	0.3000	
15	26	2.4467701	2.4467701	0.3886835	0.3000	
16	27	2.5944629	2.5944629	0.4121453	0.3000	
17	28	2.7421557	2.7421557	0.4356072	0.3000	
18	29	2.7936058	2.7936058	0.4437803	0.3000	
19	30	3.0155252	3.0155252	0.4790335	0.3000	
20	31	3.2415432	3.2415432	0.5149377	0.3000	

Cut-Points		Contal and O'Quigley Method				
**Cut Level**	**NTBNP_CD_BL**	**SK**	**Absolute SK**	**Q Statistic**	**P-value**	**Selected Cut-Point**
21	32	3.3410782	3.3410782	0.5307494	0.3000	
22	32.4	4.2142808	4.2142808	0.6694627	0.3000	
23	32.6	4.2370573	4.2370573	0.6730809	0.3000	
24	33	4.2370573	4.2370573	0.6730809	0.3000	
25	33.033	4.5781574	4.5781574	0.7272666	0.3000	
26	33.88	4.9001077	4.9001077	0.7784102	0.3000	
27	34	5.6324309	5.6324309	0.8947439	0.3000	
28	36	5.6324309	5.6324309	0.8947439	0.3000	
29	37	5.7555485	5.7555485	0.9143019	0.3000	
30	38	5.7555485	5.7555485	0.9143019	0.3000	
31	39	5.8986396	5.8986396	0.9370327	0.3000	
32	40	6.1522823	6.1522823	0.9773253	0.3000	

Cut-Points		Contal and O'Quigley Method				
**Cut Level**	**NTBNP_CD_BL**	**SK**	**Absolute SK**	**Q Statistic**	**P-value**	**Selected Cut-Point**
33	41	6.1522823	6.1522823	0.9773253	0.3000	
34	42	6.2407048	6.2407048	0.9913718	0.3000	
35	42.35	6.2574299	6.2574299	0.9940286	0.3000	
36	44	6.727073	6.727073	1.0686341	0.2038	
37	45	7.5527156	7.5527156	1.1997922	0.1124	
38	46	7.6832407	7.6832407	1.2205269	0.1016	
39	47	8.0835592	8.0835592	1.2841197	0.0739	
40	49	8.3757606	8.3757606	1.3305376	0.0580	
41	50	8.9017733	8.9017733	1.4140977	0.0367	
42	51	8.9017733	8.9017733	1.4140977	0.0367	
43	52	9.1520995	9.1520995	1.4538635	0.0292	
44	53	9.1887822	9.1887822	1.4596908	0.0282	

Cut-Points		Contal and O'Quigley Method				
**Cut Level**	**NTBNP_CD_BL**	**SK**	**Absolute SK**	**Q Statistic**	**P-value**	**Selected Cut-Point**
45	54	9.3750309	9.3750309	1.4892774	0.0237	
46	55	9.3750309	9.3750309	1.4892774	0.0237	
47	56	9.6212662	9.6212662	1.5283933	0.0187	
48	57	9.935656	9.935656	1.5783359	0.0137	
49	58	10.083349	10.083349	1.6017978	0.0118	
50	59	10.283369	10.283369	1.6335722	0.0096	
51	60	10.283369	10.283369	1.6335722	0.0096	
52	61	10.400314	10.400314	1.6521496	0.0085	
53	62	10.539	10.539	1.6741806	0.0074	
54	63	10.789326	10.789326	1.7139463	0.0056	
55	64	11.361602	11.361602	1.8048557	0.0030	
56	66	11.413053	11.413053	1.8130289	0.0028	

Cut-Points		Contal and O'Quigley Method				
**Cut Level**	**NTBNP_CD_BL**	**SK**	**Absolute SK**	**Q Statistic**	**P-value**	**Selected Cut-Point**
57	67	11.525091	11.525091	1.8308268	0.0025	
58	68	11.642036	11.642036	1.8494042	0.0021	
59	69	11.693486	11.693486	1.8575773	0.0020	
60	71	12.015436	12.015436	1.908721	0.0014	
61	72	12.163129	12.163129	1.9321828	0.0011	
62	73	12.251552	12.251552	1.9462293	0.0010	
63	74	12.468254	12.468254	1.9806537	0.0008	
64	75	12.71449	12.71449	2.0197696	0.0006	
65	76	12.884959	12.884959	2.0468496	0.0005	
66	76.23	12.884959	12.884959	2.0468496	0.0005	
67	77	13.528859	13.528859	2.1491369	0.0002	
68	81	13.651977	13.651977	2.1686949	0.0002	

Cut-Points		Contal and O'Quigley Method				
**Cut Level**	**NTBNP_CD_BL**	**SK**	**Absolute SK**	**Q Statistic**	**P-value**	**Selected Cut-Point**
69	82	13.76304	13.76304	2.1863378	0.0001	
70	83	13.831904	13.831904	2.1972773	0.0001	
71	84.7	14.153854	14.153854	2.2484209	<.0001	
72	85	14.475805	14.475805	2.2995646	<.0001	
73	87	14.92828	14.92828	2.3714429	<.0001	
74	88	15.066237	15.066237	2.3933582	<.0001	
75	89	15.066237	15.066237	2.3933582	<.0001	
76	90	15.278814	15.278814	2.4271273	<.0001	
77	91	15.426507	15.426507	2.4505891	<.0001	
78	93	15.495371	15.495371	2.4615286	<.0001	
79	94	15.495371	15.495371	2.4615286	<.0001	
80	95	15.60657	15.60657	2.4791932	<.0001	

Cut-Points		Contal and O'Quigley Method				
**Cut Level**	**NTBNP_CD_BL**	**SK**	**Absolute SK**	**Q Statistic**	**P-value**	**Selected Cut-Point**
81	96	15.819147	15.819147	2.5129623	<.0001	
82	97	15.942265	15.942265	2.5325202	<.0001	
83	98	16.517858	16.517858	2.6239565	<.0001	
84	99	16.65957	16.65957	2.6464683	<.0001	
85	100	16.841546	16.841546	2.6753761	<.0001	
86	101	17.163496	17.163496	2.7265198	<.0001	
87	101.64	17.608564	17.608564	2.7972214	<.0001	
88	102	17.608564	17.608564	2.7972214	<.0001	
89	103	17.731682	17.731682	2.8167793	<.0001	
90	104	17.731682	17.731682	2.8167793	<.0001	
91	106	17.825599	17.825599	2.8316986	<.0001	
92	108	17.956124	17.956124	2.8524333	<.0001	

Cut-Points		Contal and O'Quigley Method				
**Cut Level**	**NTBNP_CD_BL**	**SK**	**Absolute SK**	**Q Statistic**	**P-value**	**Selected Cut-Point**
93	109	18.095996	18.095996	2.8746528	<.0001	
94	110	18.212941	18.212941	2.8932302	<.0001	
95	110.11	18.433475	18.433475	2.9282633	<.0001	
96	112	18.755425	18.755425	2.9794069	<.0001	
97	113	18.755425	18.755425	2.9794069	<.0001	
98	116	18.878543	18.878543	2.9989649	<.0001	
99	117	18.878543	18.878543	2.9989649	<.0001	
100	122	18.878543	18.878543	2.9989649	<.0001	
101	124	19.001661	19.001661	3.0185228	<.0001	
102	125	19.053111	19.053111	3.026696	<.0001	
103	129	19.375061	19.375061	3.0778396	<.0001	
104	131	19.375061	19.375061	3.0778396	<.0001	

Cut-Points		Contal and O'Quigley Method				
**Cut Level**	**NTBNP_CD_BL**	**SK**	**Absolute SK**	**Q Statistic**	**P-value**	**Selected Cut-Point**
105	135	19.375061	19.375061	3.0778396	<.0001	
106	136	19.587638	19.587638	3.1116087	<.0001	
107	137	19.681555	19.681555	3.1265279	<.0001	
108	138	19.681555	19.681555	3.1265279	<.0001	
109	140	19.681555	19.681555	3.1265279	<.0001	
110	142	19.681555	19.681555	3.1265279	<.0001	
111	144	19.681555	19.681555	3.1265279	<.0001	
112	145	19.689716	19.689716	3.1278243	<.0001	
113	146	19.886163	19.886163	3.1590311	<.0001	
114	147	19.946197	19.946197	3.1685679	<.0001	
115	150	19.946197	19.946197	3.1685679	<.0001	
116	153	20.14838	20.14838	3.2006857	<.0001	

Cut-Points		Contal and O'Quigley Method				
**Cut Level**	**NTBNP_CD_BL**	**SK**	**Absolute SK**	**Q Statistic**	**P-value**	**Selected Cut-Point**
117	155	20.151068	20.151068	3.2011127	<.0001	
118	159	20.180682	20.180682	3.2058171	<.0001	
119	163	20.798942	20.798942	3.3040312	<.0001	
120	165	21.049268	21.049268	3.3437969	<.0001	
121	166	21.13769	21.13769	3.3578433	<.0001	
122	169	21.285383	21.285383	3.3813052	<.0001	
123	170	21.3793	21.3793	3.3962244	<.0001	
124	171	21.575748	21.575748	3.4274313	<.0001	
125	174	21.608846	21.608846	3.4326892	<.0001	
126	177	21.756539	21.756539	3.456151	<.0001	
127	177.87	21.879657	21.879657	3.475709	<.0001	
128	180	22.201607	22.201607	3.5268526	<.0001	

Cut-Points		Contal and O'Quigley Method				
**Cut Level**	**NTBNP_CD_BL**	**SK**	**Absolute SK**	**Q Statistic**	**P-value**	**Selected Cut-Point**
129	182	22.354909	22.354909	3.5512055	<.0001	
130	188	22.502602	22.502602	3.5746673	<.0001	
131	190	22.625719	22.625719	3.5942253	<.0001	
132	201	22.625719	22.625719	3.5942253	<.0001	
133	202	22.807461	22.807461	3.623096	<.0001	
134	205	22.895884	22.895884	3.6371425	<.0001	
135	206	22.895884	22.895884	3.6371425	<.0001	
136	213	23.026409	23.026409	3.6578771	<.0001	
137	218	23.276735	23.276735	3.6976429	<.0001	
138	220.22	23.276735	23.276735	3.6976429	<.0001	
139	236	23.598685	23.598685	3.7487865	<.0001	
140	237	23.598685	23.598685	3.7487865	<.0001	

Cut-Points		Contal and O'Quigley Method				
**Cut Level**	**NTBNP_CD_BL**	**SK**	**Absolute SK**	**Q Statistic**	**P-value**	**Selected Cut-Point**
141	243	23.68198	23.68198	3.7620183	<.0001	
142	253	23.68198	23.68198	3.7620183	<.0001	
143	259	23.829672	23.829672	3.7854801	<.0001	
144	262	24.125982	24.125982	3.8325506	<.0001	
145	265	24.214404	24.214404	3.846597	<.0001	
146	268	24.287941	24.287941	3.8582788	<.0001	
147	276	24.376364	24.376364	3.8723252	<.0001	
148	277	24.427814	24.427814	3.8804984	<.0001	
149	279	24.550932	24.550932	3.9000563	<.0001	
150	279.51	24.550932	24.550932	3.9000563	<.0001	
151	284	24.872882	24.872882	3.9512	<.0001	
152	296	25.06933	25.06933	3.9824069	<.0001	

Cut-Points		Contal and O'Quigley Method				
**Cut Level**	**NTBNP_CD_BL**	**SK**	**Absolute SK**	**Q Statistic**	**P-value**	**Selected Cut-Point**
153	296.45	25.06933	25.06933	3.9824069	<.0001	
154	303	25.06933	25.06933	3.9824069	<.0001	
155	304	25.102428	25.102428	3.9876647	<.0001	
156	305	25.110589	25.110589	3.9889611	<.0001	
157	308	25.158208	25.158208	3.9965256	<.0001	
158	321	25.480158	25.480158	4.0476692	<.0001	
159	322	25.549022	25.549022	4.0586087	<.0001	
160	325	25.571799	25.571799	4.0622269	<.0001	<====
161	326	24.839125	24.839125	3.9458374	<.0001	
162	329	24.839125	24.839125	3.9458374	<.0001	
163	330	24.839125	24.839125	3.9458374	<.0001	
164	333	23.903544	23.903544	3.7972151	<.0001	

Cut-Points		Contal and O'Quigley Method				
**Cut Level**	**NTBNP_CD_BL**	**SK**	**Absolute SK**	**Q Statistic**	**P-value**	**Selected Cut-Point**
165	337	23.991967	23.991967	3.8112616	<.0001	
166	338.8	23.991967	23.991967	3.8112616	<.0001	
167	345	23.991967	23.991967	3.8112616	<.0001	
168	346	23.991967	23.991967	3.8112616	<.0001	
169	349	23.991967	23.991967	3.8112616	<.0001	
170	357	24.025065	24.025065	3.8165194	<.0001	
171	358	24.047842	24.047842	3.8201376	<.0001	
172	372	24.195535	24.195535	3.8435995	<.0001	
173	373	24.517485	24.517485	3.8947431	<.0001	
174	385	24.517485	24.517485	3.8947431	<.0001	
175	387	24.713933	24.713933	3.92595	<.0001	
176	399	24.844458	24.844458	3.9466847	<.0001	

Cut-Points		Contal and O'Quigley Method				
**Cut Level**	**NTBNP_CD_BL**	**SK**	**Absolute SK**	**Q Statistic**	**P-value**	**Selected Cut-Point**
177	414.2	25.013542	25.013542	3.9735446	<.0001	
178	423	25.024511	25.024511	3.9752871	<.0001	
179	437.052	25.038338	25.038338	3.9774836	<.0001	
180	445	25.22008	25.22008	4.0063543	<.0001	
181	452	25.343197	25.343197	4.0259123	<.0001	
182	465.85	25.562145	25.562145	4.0606933	<.0001	
183	469.1	24.584921	24.584921	3.9054558	<.0001	
184	474	24.658459	24.658459	3.9171376	<.0001	
185	488.9	24.718493	24.718493	3.9266743	<.0001	
186	499.73	23.835438	23.835438	3.786396	<.0001	
187	513	23.958555	23.958555	3.8059539	<.0001	
188	538	23.958555	23.958555	3.8059539	<.0001	

Cut-Points		Contal and O'Quigley Method				
**Cut Level**	**NTBNP_CD_BL**	**SK**	**Absolute SK**	**Q Statistic**	**P-value**	**Selected Cut-Point**
189	540	24.010005	24.010005	3.8141271	<.0001	
190	544	24.179089	24.179089	3.840987	<.0001	
191	566	24.201866	24.201866	3.8446052	<.0001	
192	576	24.201866	24.201866	3.8446052	<.0001	
193	642	23.452192	23.452192	3.7255152	<.0001	
194	645	23.452192	23.452192	3.7255152	<.0001	
195	649	22.683287	22.683287	3.6033703	<.0001	
196	674	21.756825	21.756825	3.4561964	<.0001	
197	677.6	20.789923	20.789923	3.3025985	<.0001	
198	680	21.111873	21.111873	3.3537422	<.0001	
199	703.01	21.111873	21.111873	3.3537422	<.0001	
200	729	21.293615	21.293615	3.3826129	<.0001	

Cut-Points		Contal and O'Quigley Method				
**Cut Level**	**NTBNP_CD_BL**	**SK**	**Absolute SK**	**Q Statistic**	**P-value**	**Selected Cut-Point**
201	744	21.31034	21.31034	3.3852698	<.0001	
202	748	21.327065	21.327065	3.3879267	<.0001	
203	753	21.349842	21.349842	3.3915448	<.0001	
204	755	21.531584	21.531584	3.4204156	<.0001	
205	775	21.531584	21.531584	3.4204156	<.0001	
206	784	21.531584	21.531584	3.4204156	<.0001	
207	799	21.531584	21.531584	3.4204156	<.0001	
208	800	21.728032	21.728032	3.4516225	<.0001	
209	803	21.757646	21.757646	3.4563269	<.0001	
210	807	21.939388	21.939388	3.4851976	<.0001	
211	810	21.939388	21.939388	3.4851976	<.0001	
212	813	21.939388	21.939388	3.4851976	<.0001	

Cut-Points		Contal and O'Quigley Method				
**Cut Level**	**NTBNP_CD_BL**	**SK**	**Absolute SK**	**Q Statistic**	**P-value**	**Selected Cut-Point**
213	821	21.939388	21.939388	3.4851976	<.0001	
214	851	22.062506	22.062506	3.5047556	<.0001	
215	887	22.17945	22.17945	3.5233329	<.0001	
216	889	22.202227	22.202227	3.5269511	<.0001	
217	927	22.34992	22.34992	3.5504129	<.0001	
218	939	22.34992	22.34992	3.5504129	<.0001	
219	960	22.519003	22.519003	3.5772729	<.0001	
220	1076	22.527164	22.527164	3.5785692	<.0001	
221	1093	22.527164	22.527164	3.5785692	<.0001	
222	1098	22.650282	22.650282	3.5981271	<.0001	
223	1146	22.701732	22.701732	3.6063003	<.0001	
224	1149	22.790154	22.790154	3.6203467	<.0001	

Cut-Points		Contal and O'Quigley Method				
**Cut Level**	**NTBNP_CD_BL**	**SK**	**Absolute SK**	**Q Statistic**	**P-value**	**Selected Cut-Point**
225	1151.92	22.97453	22.97453	3.6496358	<.0001	
226	1158	22.997306	22.997306	3.653254	<.0001	
227	1159	23.044925	23.044925	3.6608185	<.0001	
228	1193	22.104959	22.104959	3.5114995	<.0001	
229	1215	22.104959	22.104959	3.5114995	<.0001	
230	1219.68	21.188253	21.188253	3.3658756	<.0001	
231	1225	21.188253	21.188253	3.3658756	<.0001	
232	1232	21.188253	21.188253	3.3658756	<.0001	
233	1234	21.40083	21.40083	3.3996446	<.0001	
234	1244	21.40083	21.40083	3.3996446	<.0001	
235	1247	21.460864	21.460864	3.4091814	<.0001	
236	1274	21.629948	21.629948	3.4360413	<.0001	

Cut-Points		Contal and O'Quigley Method				
**Cut Level**	**NTBNP_CD_BL**	**SK**	**Absolute SK**	**Q Statistic**	**P-value**	**Selected Cut-Point**
237	1290	20.826396	20.826396	3.3083924	<.0001	
238	1320	20.882271	20.882271	3.3172685	<.0001	
239	1422	19.987553	19.987553	3.1751374	<.0001	
240	1424	20.309503	20.309503	3.2262811	<.0001	
241	1487	20.559829	20.559829	3.2660468	<.0001	
242	1495	20.559829	20.559829	3.2660468	<.0001	
243	1632	20.582606	20.582606	3.269665	<.0001	
244	1649	20.6659	20.6659	3.2828968	<.0001	
245	1654	20.6659	20.6659	3.2828968	<.0001	
246	1661	20.6659	20.6659	3.2828968	<.0001	
247	1688	20.6659	20.6659	3.2828968	<.0001	
248	1702	20.6659	20.6659	3.2828968	<.0001	

Cut-Points		Contal and O'Quigley Method				
**Cut Level**	**NTBNP_CD_BL**	**SK**	**Absolute SK**	**Q Statistic**	**P-value**	**Selected Cut-Point**
249	1707	20.834984	20.834984	3.3097567	<.0001	
250	1726	20.85776	20.85776	3.3133749	<.0001	
251	1762	20.941055	20.941055	3.3266067	<.0001	
252	1763	20.943743	20.943743	3.3270337	<.0001	
253	1768	21.012607	21.012607	3.3379732	<.0001	
254	1769	21.012607	21.012607	3.3379732	<.0001	
255	1810	21.181691	21.181691	3.3648331	<.0001	
256	1822	21.181691	21.181691	3.3648331	<.0001	
257	1824	21.22931	21.22931	3.3723976	<.0001	
258	1876	21.22931	21.22931	3.3723976	<.0001	
259	1883	21.22931	21.22931	3.3723976	<.0001	
260	1900	21.22931	21.22931	3.3723976	<.0001	

Cut-Points		Contal and O'Quigley Method				
**Cut Level**	**NTBNP_CD_BL**	**SK**	**Absolute SK**	**Q Statistic**	**P-value**	**Selected Cut-Point**
261	1903	21.22931	21.22931	3.3723976	<.0001	
262	1906	21.357833	21.357833	3.3928143	<.0001	
263	1909	21.357833	21.357833	3.3928143	<.0001	
264	1910	21.357833	21.357833	3.3928143	<.0001	
265	1921	21.357833	21.357833	3.3928143	<.0001	
266	1933	21.409283	21.409283	3.4009874	<.0001	
267	1966	20.581441	20.581441	3.2694799	<.0001	
268	1987	19.637164	19.637164	3.1194762	<.0001	
269	1990	19.637164	19.637164	3.1194762	<.0001	
270	2036	19.637164	19.637164	3.1194762	<.0001	
271	2065	19.666779	19.666779	3.1241806	<.0001	
272	2084	19.666779	19.666779	3.1241806	<.0001	

Cut-Points		Contal and O'Quigley Method				
**Cut Level**	**NTBNP_CD_BL**	**SK**	**Absolute SK**	**Q Statistic**	**P-value**	**Selected Cut-Point**
273	2109	18.805851	18.805851	2.9874172	<.0001	
274	2173	18.822576	18.822576	2.9900741	<.0001	
275	2177	18.836402	18.836402	2.9922705	<.0001	
276	2216	18.836402	18.836402	2.9922705	<.0001	
277	2226	18.859179	18.859179	2.9958887	<.0001	
278	2233	18.870148	18.870148	2.9976313	<.0001	
279	2264	19.166457	19.166457	3.0447017	<.0001	
280	2270	18.289575	18.289575	2.9054039	<.0001	
281	2279	17.562629	17.562629	2.7899243	<.0001	
282	2288	17.585405	17.585405	2.7935425	<.0001	
283	2301	17.708523	17.708523	2.8131004	<.0001	
284	2313	17.708523	17.708523	2.8131004	<.0001	

Cut-Points		Contal and O'Quigley Method				
**Cut Level**	**NTBNP_CD_BL**	**SK**	**Absolute SK**	**Q Statistic**	**P-value**	**Selected Cut-Point**
285	2352	17.914935	17.914935	2.8458901	<.0001	
286	2353	17.92034	17.92034	2.8467488	<.0001	
287	2365	17.92034	17.92034	2.8467488	<.0001	
288	2414	17.92034	17.92034	2.8467488	<.0001	
289	2457	17.92034	17.92034	2.8467488	<.0001	
290	2459	18.089424	18.089424	2.8736088	<.0001	
291	2513	18.089424	18.089424	2.8736088	<.0001	
292	2515	18.112201	18.112201	2.8772269	<.0001	
293	2527	18.134977	18.134977	2.8808451	<.0001	
294	2583	18.182596	18.182596	2.8884096	<.0001	
295	2584	18.182596	18.182596	2.8884096	<.0001	
296	2659	18.182596	18.182596	2.8884096	<.0001	

Cut-Points		Contal and O'Quigley Method				
**Cut Level**	**NTBNP_CD_BL**	**SK**	**Absolute SK**	**Q Statistic**	**P-value**	**Selected Cut-Point**
297	2676.52	18.199321	18.199321	2.8910665	<.0001	
298	2678	18.199321	18.199321	2.8910665	<.0001	
299	2721	18.216046	18.216046	2.8937234	<.0001	
300	2762	18.238822	18.238822	2.8973416	<.0001	
301	2815	18.560773	18.560773	2.9484852	<.0001	
302	2940	18.560773	18.560773	2.9484852	<.0001	
303	2965	18.560773	18.560773	2.9484852	<.0001	
304	3025	18.639141	18.639141	2.9609345	<.0001	
305	3050	18.639141	18.639141	2.9609345	<.0001	
306	3089	18.639141	18.639141	2.9609345	<.0001	
307	3090	17.738676	17.738676	2.8178904	<.0001	
308	3102	17.76829	17.76829	2.8225948	<.0001	

Cut-Points		Contal and O'Quigley Method				
**Cut Level**	**NTBNP_CD_BL**	**SK**	**Absolute SK**	**Q Statistic**	**P-value**	**Selected Cut-Point**
309	3123	17.81974	17.81974	2.8307679	<.0001	
310	3151	17.852839	17.852839	2.8360258	<.0001	
311	3191	17.852839	17.852839	2.8360258	<.0001	
312	3199	17.852839	17.852839	2.8360258	<.0001	
313	3209	16.904289	16.904289	2.6853432	<.0001	
314	3270	17.034814	17.034814	2.7060779	<.0001	
315	3303	17.034814	17.034814	2.7060779	<.0001	
316	3317	17.05759	17.05759	2.7096961	<.0001	
317	3345.65	17.080367	17.080367	2.7133143	<.0001	
318	3380	17.402317	17.402317	2.7644579	<.0001	
319	3445	17.425094	17.425094	2.7680761	<.0001	
320	3500	17.524629	17.524629	2.7838878	<.0001	

Cut-Points		Contal and O'Quigley Method				
**Cut Level**	**NTBNP_CD_BL**	**SK**	**Absolute SK**	**Q Statistic**	**P-value**	**Selected Cut-Point**
321	3513	17.524629	17.524629	2.7838878	<.0001	
322	3560	17.524629	17.524629	2.7838878	<.0001	
323	3649	17.524629	17.524629	2.7838878	<.0001	
324	3654	17.572247	17.572247	2.7914523	<.0001	
325	3681	17.623698	17.623698	2.7996255	<.0001	
326	3688	16.637524	16.637524	2.6429661	<.0001	
327	3705	16.711061	16.711061	2.654648	<.0001	
328	3711	16.794356	16.794356	2.6678797	<.0001	
329	3833	16.817132	16.817132	2.6714979	<.0001	
330	3840	16.817132	16.817132	2.6714979	<.0001	
331	3859	16.029709	16.029709	2.5464112	<.0001	
332	3887	16.029709	16.029709	2.5464112	<.0001	

Cut-Points		Contal and O'Quigley Method				
**Cut Level**	**NTBNP_CD_BL**	**SK**	**Absolute SK**	**Q Statistic**	**P-value**	**Selected Cut-Point**
333	3925	16.077328	16.077328	2.5539758	<.0001	
334	3945	16.082733	16.082733	2.5548345	<.0001	
335	3956	15.230426	15.230426	2.4194406	<.0001	
336	3959	15.230426	15.230426	2.4194406	<.0001	
337	4038	14.278045	14.278045	2.2681493	<.0001	
338	4065.6	14.300821	14.300821	2.2717675	<.0001	
339	4139	14.300821	14.300821	2.2717675	<.0001	
340	4171	14.300821	14.300821	2.2717675	<.0001	
341	4256	14.300821	14.300821	2.2717675	<.0001	
342	4334	14.323598	14.323598	2.2753857	<.0001	
343	4357	14.340323	14.340323	2.2780426	<.0001	
344	4401	14.340323	14.340323	2.2780426	<.0001	

Cut-Points		Contal and O'Quigley Method				
**Cut Level**	**NTBNP_CD_BL**	**SK**	**Absolute SK**	**Q Statistic**	**P-value**	**Selected Cut-Point**
345	4403	14.340323	14.340323	2.2780426	<.0001	
346	4422	14.391773	14.391773	2.2862157	<.0001	
347	4446.75	14.41455	14.41455	2.2898339	<.0001	
348	4481	14.596292	14.596292	2.3187047	<.0001	
349	4484	14.778034	14.778034	2.3475754	<.0001	
350	4529	14.894979	14.894979	2.3661528	<.0001	
351	4612	14.894979	14.894979	2.3661528	<.0001	
352	4659	14.917755	14.917755	2.369771	<.0001	
353	4702	15.04828	15.04828	2.3905056	<.0001	
354	4729	15.04828	15.04828	2.3905056	<.0001	
355	4783	15.04828	15.04828	2.3905056	<.0001	
356	4803	15.147815	15.147815	2.4063173	<.0001	

Cut-Points		Contal and O'Quigley Method				
**Cut Level**	**NTBNP_CD_BL**	**SK**	**Absolute SK**	**Q Statistic**	**P-value**	**Selected Cut-Point**
357	4862	15.170592	15.170592	2.4099355	<.0001	
358	4866	15.170592	15.170592	2.4099355	<.0001	
359	4954	15.193368	15.193368	2.4135537	<.0001	
360	4998	15.193368	15.193368	2.4135537	<.0001	
361	5014	15.193368	15.193368	2.4135537	<.0001	
362	5025	15.193368	15.193368	2.4135537	<.0001	
363	5291	15.204338	15.204338	2.4152962	<.0001	
364	5473	15.255788	15.255788	2.4234694	<.0001	
365	5476	15.255788	15.255788	2.4234694	<.0001	
366	5594	15.386313	15.386313	2.444204	<.0001	
367	5725	15.437763	15.437763	2.4523772	<.0001	
368	5922	14.506627	14.506627	2.3044609	<.0001	

Cut-Points		Contal and O'Quigley Method				
**Cut Level**	**NTBNP_CD_BL**	**SK**	**Absolute SK**	**Q Statistic**	**P-value**	**Selected Cut-Point**
369	6013.7	14.520454	14.520454	2.3066574	<.0001	
370	6222	14.571904	14.571904	2.3148305	<.0001	
371	6312	14.571904	14.571904	2.3148305	<.0001	
372	6729	14.753646	14.753646	2.3437013	<.0001	
373	6804	14.753646	14.753646	2.3437013	<.0001	
374	6806	14.805096	14.805096	2.3518744	<.0001	
375	6866	14.805096	14.805096	2.3518744	<.0001	
376	6977	14.856546	14.856546	2.3600476	<.0001	
377	7136	14.856546	14.856546	2.3600476	<.0001	
378	7402.78	15.106872	15.106872	2.3998133	<.0001	
379	7415	15.106872	15.106872	2.3998133	<.0001	
380	7532	15.428823	15.428823	2.450957	<.0001	

**Cut-Points**		**Contal and O'Quigley Method**				
**Cut Level**	**NTBNP_CD_BL**	**SK**	**Absolute SK**	**Q Statistic**	**P-value**	**Selected Cut-Point**
381	7589	14.472765	14.472765	2.2990817	<.0001	
382	7669	14.480925	14.480925	2.300378	<.0001	
383	8004	13.559294	13.559294	2.1539715	0.0002	
384	8247	13.603236	13.603236	2.160952	0.0002	
385	8416	13.68653	13.68653	2.1741838	0.0002	
386	8538	13.68653	13.68653	2.1741838	0.0002	
387	8922	13.73798	13.73798	2.182357	0.0001	
388	9041	12.826403	12.826403	2.0375476	0.0005	
389	9408	12.826403	12.826403	2.0375476	0.0005	
390	9622	12.826403	12.826403	2.0375476	0.0005	
391	10535	12.937465	12.937465	2.0551906	0.0004	
392	10909	12.95419	12.95419	2.0578474	0.0004	

Cut-Points		Contal and O'Quigley Method				
**Cut Level**	**NTBNP_CD_BL**	**SK**	**Absolute SK**	**Q Statistic**	**P-value**	**Selected Cut-Point**
393	11035	12.123274	12.123274	1.9258516	0.0012	
394	11938	11.152888	11.152888	1.7717003	0.0038	
395	12156	11.185987	11.185987	1.7769581	0.0036	
396	13056	11.185987	11.185987	1.7769581	0.0036	
397	13153.91	11.229929	11.229929	1.7839386	0.0034	
398	13732	10.266612	10.266612	1.6309101	0.0098	
399	14021	10.266612	10.266612	1.6309101	0.0098	
400	14047	10.266612	10.266612	1.6309101	0.0098	
401	14534.52	9.3069175	9.3069175	1.4784572	0.0253	
402	14557	8.3178868	8.3178868	1.321344	0.0609	
403	15967	8.3178868	8.3178868	1.321344	0.0609	
404	16499.56	7.4289493	7.4289493	1.1801312	0.1234	

Cut-Points		Contal and O'Quigley Method				
**Cut Level**	**NTBNP_CD_BL**	**SK**	**Absolute SK**	**Q Statistic**	**P-value**	**Selected Cut-Point**
405	17722	6.4551388	6.4551388	1.0254358	0.2442	
406	17947.93	6.6028316	6.6028316	1.0488977	0.2215	
407	21048	5.6225778	5.6225778	0.8931787	0.3000	
408	21067	4.7531029	4.7531029	0.7550577	0.3000	
409	21075	4.7531029	4.7531029	0.7550577	0.3000	
410	21539.21	4.7531029	4.7531029	0.7550577	0.3000	
411	21810.25	4.7698279	4.7698279	0.7577145	0.3000	
412	23731	3.863745	3.863745	0.613778	0.3000	
413	25000	3.863745	3.863745	0.613778	0.3000	
414	25096	2.8719053	2.8719053	0.4562186	0.3000	
415	25118	2.1682146	2.1682146	0.3444333	0.3000	
416	26079.13	2.1849397	2.1849397	0.3470902	0.3000	

Cut-Points		Contal and O'Quigley Method				
**Cut Level**	**NTBNP_CD_BL**	**SK**	**Absolute SK**	**Q Statistic**	**P-value**	**Selected Cut-Point**
417	26196	2.50689	2.50689	0.3982339	0.3000	
418	26832.96	2.5757544	2.5757544	0.4091734	0.3000	
419	27949	2.8977047	2.8977047	0.460317	0.3000	
420	28203	2.9086741	2.9086741	0.4620596	0.3000	
421	30000	2.9086741	2.9086741	0.4620596	0.3000	
422	31738	1.9140796	1.9140796	0.3040625	0.3000	
423	35277.55	1.0958216	1.0958216	0.1740776	0.3000	
424	44636.9	1.2435144	1.2435144	0.1975394	0.3000	
425	45077.34	0.5654647	0.5654647	0.0898273	0.3000	
426	49778.19	0.6130834	0.6130834	0.0973918	0.3000	
427	50125.46	0.6130834	0.6130834	0.0973918	0.3000	
428	78542.31	0.6358599	0.6358599	0.10101	0.3000	

Cut-Points		Contal and O'Quigley Method				
**Cut Level**	**NTBNP_CD_BL**	**SK**	**Absolute SK**	**Q Statistic**	**P-value**	**Selected Cut-Point**
429	117919.34	0.6358599	0.6358599	0.10101	0.3000	
430	118139.56	0.6586365	0.6586365	0.1046282	0.3000	
431	213350.83	0.9805868	0.9805868	0.1557718	0.3000	
432	296450	-0.002688	0.0026882	0.000427	0.3000	
