# Supplementary material for: Impact of genotype and phenotype on cardiac biomarkers in patients with transthyretin amyloidosis – Report from the Transthyretin Amyloidosis Outcome Survey (THAOS)
Source: PLoS One. 2017 Apr 6;12(4):e0173086. doi: 10.1371/journal.pone.0173086 (PMC5383030; doi:10.1371/journal.pone.0173086)
Supplement: S1 Supporting Information — (ZIP) [file pone.0173086.s001.zip › S15_Table_List of active Investigators 20160920.docx]

| [Investigator](http://rightrack.pfizer.com/RighTrack/appmanager/portal/desktop?_nfpb=true&PI_Protocol_Parent_actionOverride=%2Fpageflows%2Frightrack%2Fprotocol%2FstudyCenters%2Fsort&_windowLabel=PI_Protocol_Parent&PI_Protocol_Parent_sortcol=19) | Institution |
| --- | --- |
| Dr. Fabio Adrian Barroso | Instituto de Investigaciones Neurológicas Raúl Carrea,  Montañeses 2325,  Buenos Aires,  ARGENTINA |
| Dr Marcelo F Rugiero | Hospital Italiano de Buenos Aires,  Juan D. Peron 4190,  Buenos Aires, C1199ABD,  ARGENTINA |
| Dr. Johan J. Van Cleemput | Afdeling Klinische Cardiologie  O&N I  Herestraat 49 - bus 7003  Leuven, 3000  BELGIUM |
| Prof. Ivailo Tournev | Clinic of Dermatology and Venereology University Hospital Alexandrovska  1 Georgi Sofiisky Street  Sofia, 1431  BULGARIA |
| Dr. Marcia Waddington Cruz | Hospital Universitario Clementino Fraga Filho  Rua Rodolpho Paulo Rocco, 255-30 andar  Ilha do Fundao  Rio de Janeiro, 21941-913  BRAZIL |
| Dr. Nowell M. Fine | Libin Cardiovascular Institute of Alberta  South Health Campus  4448 Front Street SE  Calgary, AB T3M 1M4  CANADA |
| Dr. Arnt Volko Kristen | Universitatsklinikum Heidelberg  Im Neuenheimer Feld 410  Heidelberg, Baden-wurttemberg 69120  GERMANY |
| Prof. Hartmut H.J. Schmidt | Universitaetsklinikum Muenster-Klinik fur Frauenheilkunde und Geburtshilfe  Zentralklinikum  Albert-Schweitzer-Campus 1  Muenster, 48149  GERMANY |
| Prof. Dr. Tim Zimmermann | Universitaetsmedizin der Johannes Gutenberg-Universitaet Mainz  Innere Medizin,Zentrum fuer Klinische Studien (ZKS) am Zentrum fuer Kinder- und Jugendmedizin -  Langenbeckstrasse 1,Gebaude 605  Mainz, Rheinland-Pfalz 55131  GERMANY |
| Burkhard Gess | Uniklinik RWTH Aachen  Klinik fuer Neurologie und Institut fuer Neuropathologie  Pauwelsstrasse 30  Aachen, 52074  GERMANY |
| Prof Dr Med. Henning Moelgaard | Aarhus University Hospital, Skejby  Department of Cardiology B  Brendstrupgaardsvej 100  Aarhus N, 8200  DENMARK |
| Dr. Josep Maria Campistol Plana | Fundacia Clinic Per La Recerca Biomedica  Unidad de transplante renal  C/Rosello 149-153  Barcelona, 8036  SPAIN |
| Juan Buades Reines | Hospital Son Llatzer  Servicio de Cardiologia  Carretera de Manacor Km 4 s/n 3a Planta  Palma de Mallorca, Islas Baleares 07198  SPAIN |
| Jose Gonzalez Costello | Hospital Universitari de Bellvitge  Servicio de Cardiologia  Feixa Llarga s/n  08907 L'Hospitalet de Llobregat  Barcelona, 0000  SPAIN |
| Pablo Garcia Pavia | Hospital Universitario Puerta de Hierro  Cardiomyopathy Unit, Department of Cardiology  Manuel de Falla 1  Majadahonda, 28222  SPAIN |
| Dr. Jose Luis Munoz Blanco | Hospital Gregorio Marañón  Servicio de Neurologia,Hospial Gregorio Marañon  Dr. Esquerdo 46,  Madrid,, Spain. 28007  SPAIN |
| Dr. Violaine Plante-Bordeneuve | Hopital Henri Mondor  Service de Neurologie  51, avenue du Marechal de Lattre de Tassigny  Centre d'Investigation Clinique  Créteil, 94000  FRANCE |
| David Adams | CHU de Bicetre  78 Rue Du General Leclerc  Le Kremlin Bicetre, 94275  FRANCE |
| Dr. Jocelyn Inamo | Hopital Pierre Zobda-Quitman - CHU de Fort de France  BP90632  Fort de France, 97261  FRANCE |
| Dr. Claudio Rapezzi | Azienda Ospedaliero-Universitaria di Bologna - Dr. Rapezzi  Policlinico Sant'Orsala-Malpighi  Via Albertoni 15  Bologna, 40138  ITALY |
| Prof. Giuseppe Vita | AOU Policlinico G. Martino - Messina - Dr. Vita  U.O. di Neurobiologia Clinica e Malattie Neuromuscolari del Policlinico  Via Consolare Valeria  Messina, 98125  ITALY |
| Prof. Giampaolo Merlini | Centro per lo Studio e la Cura delle Amiloidosi Sistemiche - Pavia - Prof. Merlini  Fondazione IRCCS Policlinico S.Matteo Amyloidosis Research Treatment Center  P.le Golgi 19  Pavia, 27100  ITALY |
| Franco Bergesio | Azienda Ospedaliero-Universitaria di Careggi  Nefrologia, Dialisi e Medicina dei Trapianti  Largo Brambilla, 3  Firenze, Firenze 50134  ITALY |
| Dr. Yoshiki  Sekijima | Shinshu University Hospital, Shinshu University Graduate School Of Medicine  3-1-1, Asahi  Matsumoto, Nagano 390-8621  JAPAN |
| Dr. Yukio  Ando | Kumamoto University  1-1-1 Honjo  Department of Diagnostic Medicine  Kumamoto, Kumamoto 860-8556  JAPAN |
| Sonoko  Misawa | Chiba University Hospital  1-8-1 Inohana  Chuo-ku,Chiba-shi  Chiba-shi, Chiba 260-8677  JAPAN |
| Ga Yeon  Lee | Samsung Medical Center  81 Irwon-Ro, Gangnam-gu  Seoul, 06351  KOREA, REPUBLIC OF |
| Dr. Jeeyoung  Oh | Konkuk University Medical Center  120-1, Neungdong-ro  Gwangjin-gu, Seoul 143-729  KOREA, REPUBLIC OF |
| Dr. Maria Alejandra Gonzalez Duarte Briseno | Instituto Nacional De Ciencias Medicas Y Nutricion Salvador Zubiran (Incmnsz)  Jefatura de Trasplantes  Av. Vasco de Quiroga 15 Tlalpan, Belisario Domínguez Seccion XVI  Mexico, DF 14080  MEXICO |
| Dr. Bouke P.C. Hazenberg | University Medical Center Groningen  Hanzeplein 1  Groningen, 9713 GZ  NETHERLANDS |
| Dr. Teresa  Coelho | Unidade Clinica de Paramiloidose Hospital Geral de Santo Antonio  Largo Prof. Abel Salazar  Porto, 4099001  PORTUGAL |
| Dr. Isabel M. Conceicao | Centro Hospitalar Lisboa Norte E.P.E. - Hospital Santa Maria  Av. Professor Egas Moniz  Lisbon, 1649-035  PORTUGAL |
| Dr. Ole B. Suhr | Umea University Hospital  Dept. of Medicine  Umea, 901 85  SWEDEN |
| Dr. Mathew Shane Maurer | Columbia University Medical Center 2  Irving Pavilion 10th floor  5141 Broadway, Rm 3FW-035  New York, NY 10034  UNITED STATES |
| Dr. Sanjiv Jayendra Shah | Northwestern University  Suite 600  201 East Huron Street, Galter 10-240  Chicago, IL 60611  UNITED STATES |
| Dr. Dianna  Quan | UC Denver,Neurology - Academic Office 1  Department of Neurology, Academic Office 1  12631 East 17th Street,Room L15-5121  Aurora, CO 80045  UNITED STATES |
| Dr. Daniel Philip Judge | Johns Hopkins University School of Medicine  Ross Building #1049 720 Rutland Avenue  Baltimore, MD 21205  UNITED STATES |
| Dr. Stephen Scott Gottlieb | University of Maryland  110 South Paca Street  7th Floor  Baltimore, MD 21201  UNITED STATES |
| Nitasha  Sarswat | University of Chicago Medical Center  5841 South Maryland  Chicago, IL 60637  UNITED STATES |
| Dr. Srinivas C. Murali | Allegheny General Hospital, Allegheny Singer Research Institute, Cardiology Research  320 East North Avenue  8th Floor, South Tower  Pittsburgh, PA 15212  UNITED STATES |
| Stanley  Iyadurai | The Ohio University College of Medicine  395 W 12th Avenue  Neuromuscular Division, 7th Floor  Columbus, OH 43210  UNITED STATES |
| Dr. William Gerritt Cotts | Advocate Christ Medical Center  4440 West 95th Street  OPP 6411  Oak Lawn, IL 60453  UNITED STATES |
| Dr. Brian M. Drachman | Penn Philadelphia Heart Institute,Department of Surgery  Philadelphia Heart Institute  39th& Market Streets  Philadelphia, PA 19104  UNITED STATES |
| Dr. Angela  Dispenzieri | Mayo Clinic  Division of Hematology, Department of Internal Medicine  200 First Street SW  Rochester, MN 55905  UNITED STATES |
| Dr. David Eric Steidley | Mayo Clinic  Cardiovascular Diseases  5777 East Mayo Boulevard  Phoenix , AZ 85054  UNITED STATES |
| Dr. Scott L. Hummel | University of Michigan  1500 E Medical Center Drive  SPC 5853  Ann Arbor, MI 48109  UNITED STATES |
| Dr. Daniel J. Lenihan | Vanderbilt University School of Medicine  1215 21st Ave South  MCE 5th Floor  Nashville, TN 37232  UNITED STATES |
| Dr. Hector Osvaldo Ventura | John Ochsner Heart & Vascular Institute  Room 3E108  1514 Jefferson Hwy  New Orleans, LA 70121  UNITED STATES |
| Dr. Daniel L. Jacoby | Yale School of Medicine  20 York Street  New Haven, CT 06510  UNITED STATES |
| James E. Hoffman | University of Miami Hospital & Clinics  1475 NW 12th Avenue  Miami, FL 33136  UNITED STATES |
