# Supplementary material for: Impact of genotype and phenotype on cardiac biomarkers in patients with transthyretin amyloidosis – Report from the Transthyretin Amyloidosis Outcome Survey (THAOS)
Source: PLoS One. 2017 Apr 6;12(4):e0173086. doi: 10.1371/journal.pone.0173086 (PMC5383030; doi:10.1371/journal.pone.0173086)
Supplement: S1 Supporting Information — (ZIP) [file pone.0173086.s001.zip › S11_Table_C_O'Q_Optimal Cutpoint_Troponin_T.pdf]

The SAS System

OPTIMAL DICHOTOMIZATION OF CONTINUOUS VARIABLES

EXPLORATION OF CUTPOINT FOR TROPONINT_CD_BL IN EXPLANING DEATH_FL

RANGES FOR TROPONINT_CD_BL

		**Percentile**								
**Patient Subset**	**N**	**Min**	**5**	**10**	**25**	**Med**	**75**	**90**	**95**	**Max**
All	274	0.001	0.003	0.004	0.01	0.0265	0.05	0.08	0.123	1

OPTIMAL DICHOTOMIZATION OF CONTINUOUS VARIABLES

Cut-Points		Contal and O'Quigley Method				
**Cut Level**	**TROPONINT_CD_BL**	**SK**	**Absolute SK**	**Q Statistic**	**P-value**	**Selected Cut-Point**
1	0.001	0	0	0	0.3000	
2	0.003	0.7149675	0.7149675	0.1248418	0.3000	
3	0.004	1.022221	1.022221	0.1784919	0.3000	
4	0.005	1.7603982	1.7603982	0.3073863	0.3000	
5	0.006	2.0162923	2.0162923	0.3520684	0.3000	
6	0.007	1.2803325	1.2803325	0.2235612	0.3000	
7	0.008	1.4467375	1.4467375	0.2526174	0.3000	
8	0.009	1.4671072	1.4671072	0.2561742	0.3000	

Cut-Points		Contal and O'Quigley Method				
**Cut Level**	**TROPONINT_CD_BL**	**SK**	**Absolute SK**	**Q Statistic**	**P-value**	**Selected Cut-Point**
9	0.01	1.765082	1.765082	0.3082041	0.3000	
10	0.011	13.546993	13.546993	2.3654647	<.0001	
11	0.012	13.546993	13.546993	2.3654647	<.0001	
12	0.013	13.642393	13.642393	2.3821228	<.0001	
13	0.015	13.642393	13.642393	2.3821228	<.0001	
14	0.016	13.669386	13.669386	2.386836	<.0001	
15	0.017	13.669386	13.669386	2.386836	<.0001	
16	0.019	13.669386	13.669386	2.386836	<.0001	
17	0.02	13.669386	13.669386	2.386836	<.0001	
18	0.021	14.32262	14.32262	2.5008984	<.0001	<====
19	0.023	13.429132	13.429132	2.3448849	<.0001	
20	0.024	13.429132	13.429132	2.3448849	<.0001	

Cut-Points		Contal and O'Quigley Method				
**Cut Level**	**TROPONINT_CD_BL**	**SK**	**Absolute SK**	**Q Statistic**	**P-value**	**Selected Cut-Point**
21	0.025	13.458049	13.458049	2.3499341	<.0001	
22	0.026	13.458049	13.458049	2.3499341	<.0001	
23	0.027	12.692828	12.692828	2.2163174	0.0001	
24	0.028	12.921322	12.921322	2.2562152	<.0001	
25	0.029	13.47233	13.47233	2.3524277	<.0001	
26	0.03	13.47233	13.47233	2.3524277	<.0001	
27	0.031	12.832345	12.832345	2.2406788	<.0001	
28	0.033	13.11337	13.11337	2.2897491	<.0001	
29	0.034	13.11337	13.11337	2.2897491	<.0001	
30	0.035	13.189618	13.189618	2.3030628	<.0001	
31	0.036	13.189618	13.189618	2.3030628	<.0001	
32	0.038	13.203043	13.203043	2.305407	<.0001	

Cut-Points		Contal and O'Quigley Method				
**Cut Level**	**TROPONINT_CD_BL**	**SK**	**Absolute SK**	**Q Statistic**	**P-value**	**Selected Cut-Point**
33	0.039	13.203043	13.203043	2.305407	<.0001	
34	0.04	13.60687	13.60687	2.3759199	<.0001	
35	0.041	12.627798	12.627798	2.2049624	0.0001	
36	0.042	12.627798	12.627798	2.2049624	0.0001	
37	0.043	12.627798	12.627798	2.2049624	0.0001	
38	0.045	11.66541	11.66541	2.0369182	0.0005	
39	0.048	12.339638	12.339638	2.1546464	0.0002	
40	0.049	12.673989	12.673989	2.2130279	0.0001	
41	0.05	12.673989	12.673989	2.2130279	0.0001	
42	0.051	11.997336	11.997336	2.0948764	0.0003	
43	0.052	11.997336	11.997336	2.0948764	0.0003	
44	0.053	11.352963	11.352963	1.9823612	0.0008	

Cut-Points		Contal and O'Quigley Method				
**Cut Level**	**TROPONINT_CD_BL**	**SK**	**Absolute SK**	**Q Statistic**	**P-value**	**Selected Cut-Point**
45	0.054	10.40884	10.40884	1.8175063	0.0027	
46	0.06	10.526716	10.526716	1.8380888	0.0023	
47	0.063	10.447381	10.447381	1.824236	0.0026	
48	0.064	9.6678744	9.6678744	1.6881249	0.0067	
49	0.066	9.6678744	9.6678744	1.6881249	0.0067	
50	0.069	10.342103	10.342103	1.8058531	0.0029	
51	0.07	10.362472	10.362472	1.8094099	0.0029	
52	0.072	9.7224466	9.7224466	1.6976539	0.0063	
53	0.073	9.7428163	9.7428163	1.7012106	0.0061	
54	0.078	9.7428163	9.7428163	1.7012106	0.0061	
55	0.08	9.8175634	9.8175634	1.7142624	0.0056	
56	0.083	8.5223183	8.5223183	1.4880973	0.0239	

**Cut-Points**		**Contal and O'Quigley Method**				
**Cut Level**	**TROPONINT_CD_BL**	**SK**	**Absolute SK**	**Q Statistic**	**P-value**	**Selected Cut-Point**
57	0.084	7.5357435	7.5357435	1.3158297	0.0627	
58	0.09	7.5357435	7.5357435	1.3158297	0.0627	
59	0.099	7.5357435	7.5357435	1.3158297	0.0627	
60	0.1	7.5357435	7.5357435	1.3158297	0.0627	
61	0.11	5.8815406	5.8815406	1.0269864	0.2426	
62	0.118	5.215891	5.215891	0.9107561	0.3000	
63	0.12	5.215891	5.215891	0.9107561	0.3000	
64	0.121	5.2535034	5.2535034	0.9173237	0.3000	
65	0.123	5.2535034	5.2535034	0.9173237	0.3000	
66	0.134	5.2535034	5.2535034	0.9173237	0.3000	
67	0.139	4.2824201	4.2824201	0.7477611	0.3000	
68	0.14	3.3291233	3.3291233	0.5813042	0.3000	

Cut-Points		Contal and O'Quigley Method				
**Cut Level**	**TROPONINT_CD_BL**	**SK**	**Absolute SK**	**Q Statistic**	**P-value**	**Selected Cut-Point**
69	0.167	1.6678374	1.6678374	0.2912241	0.3000	
70	0.17	1.6882071	1.6882071	0.2947809	0.3000	
71	0.192	1.6882071	1.6882071	0.2947809	0.3000	
72	0.2	0.8297588	0.8297588	0.1448857	0.3000	
73	0.231	0.914407	0.914407	0.1596662	0.3000	
74	0.26	0.914407	0.914407	0.1596662	0.3000	
75	1	-0.02037	0.0203697	0.0035568	0.3000	
