# Supplementary material for: Impact of genotype and phenotype on cardiac biomarkers in patients with transthyretin amyloidosis – Report from the Transthyretin Amyloidosis Outcome Survey (THAOS)
Source: PLoS One. 2017 Apr 6;12(4):e0173086. doi: 10.1371/journal.pone.0173086 (PMC5383030; doi:10.1371/journal.pone.0173086)
Supplement: S1 Supporting Information — (ZIP) [file pone.0173086.s001.zip › S12_Table_C_O'Q_Optimal Cutpoint_Troponin_I.pdf]

OPTIMAL DICHOTOMIZATION OF CONTINUOUS VARIABLES

EXPLORATION OF CUTPOINT FOR TROPONINI_CD_BL IN EXPLANING DEATH_FL

RANGES FOR TROPONINI_CD_BL

		**Percentile**								
**Patient Subset**	**N**	**Min**	**5**	**10**	**25**	**Med**	**75**	**90**	**95**	**Max**
All	108	0	0.01	0.02	0.035	0.075	0.125	0.2	0.58	1

The SAS System

OPTIMAL DICHOTOMIZATION OF CONTINUOUS VARIABLES

Cut-Points		Contal and O'Quigley Method				
Cut Level	TROPONINI_CD_BL	SK	Absolute SK	Q Statistic	P-value	Selected Cut-Point
1	0	0	0	0	0.3000	
2	0.001	0.3655836	0.3655836	0.0760502	0.3000	
3	0.01	0.5178238	0.5178238	0.1077198	0.3000	
4	0.017	2.282496	2.282496	0.4748143	0.3000	
5	0.02	2.282496	2.282496	0.4748143	0.3000	
6	0.03	2.9691228	2.9691228	0.6176492	0.3000	
7	0.04	4.7467317	4.7467317	0.9874347	0.3000	
8	0.05	4.9788425	4.9788425	1.0357194	0.2340	

Cut-Points		Contal and O'Quigley Method				
**Cut Level**	**TROPONINI_CD_BL**	**SK**	**Absolute SK**	**Q Statistic**	**P-value**	**Selected Cut-Point**
9	0.06	5.4409589	5.4409589	1.1318507	0.1543	
10	0.063	7.9136414	7.9136414	1.6462284	0.0089	<====
11	0.07	7.279225	7.279225	1.5142544	0.0204	
12	0.08	6.7949276	6.7949276	1.4135089	0.0368	
13	0.09	6.6295371	6.6295371	1.3791037	0.0446	
14	0.1	7.2037994	7.2037994	1.4985641	0.0224	
15	0.11	4.5775156	4.5775156	0.9522337	0.3000	
16	0.12	4.5775156	4.5775156	0.9522337	0.3000	
17	0.13	2.3029201	2.3029201	0.4790629	0.3000	
18	0.14	1.5037733	1.5037733	0.3128211	0.3000	
19	0.15	0.469274	0.469274	0.0976203	0.3000	
20	0.151	1.0132408	1.0132408	0.2107785	0.3000	

Cut-Points		Contal and O'Quigley Method				
**Cut Level**	**TROPONINI_CD_BL**	**SK**	**Absolute SK**	**Q Statistic**	**P-value**	**Selected Cut-Point**
21	0.16	1.5956101	1.5956101	0.3319254	0.3000	
22	0.17	1.5956101	1.5956101	0.3319254	0.3000	
23	0.2	1.8143505	1.8143505	0.3774287	0.3000	
24	0.21	0.2732941	0.2732941	0.0568518	0.3000	
25	0.23	0.7264967	0.7264967	0.1511289	0.3000	
26	0.26	0.7264967	0.7264967	0.1511289	0.3000	
27	0.34	1.1796994	1.1796994	0.2454059	0.3000	
28	0.58	0.2058711	0.2058711	0.0428262	0.3000	
29	0.64	0.2058711	0.2058711	0.0428262	0.3000	
30	0.73	0.2058711	0.2058711	0.0428262	0.3000	
31	0.79	0.2185293	0.2185293	0.0454594	0.3000	
32	0.86	-0.682369	0.6823693	0.1419493	0.3000	

Cut-Points		Contal and O'Quigley Method				
**Cut Level**	**TROPONINI_CD_BL**	**SK**	**Absolute SK**	**Q Statistic**	**P-value**	**Selected Cut-Point**
33	1	-0.682369	0.6823693	0.1419493	0.3000	
