# Supplementary figures and images for: Impact of genotype and phenotype on cardiac biomarkers in patients with transthyretin amyloidosis – Report from the Transthyretin Amyloidosis Outcome Survey (THAOS)
Source: PLoS One. 2017 Apr 6;12(4):e0173086. doi: 10.1371/journal.pone.0173086 (PMC5383030; doi:10.1371/journal.pone.0173086)

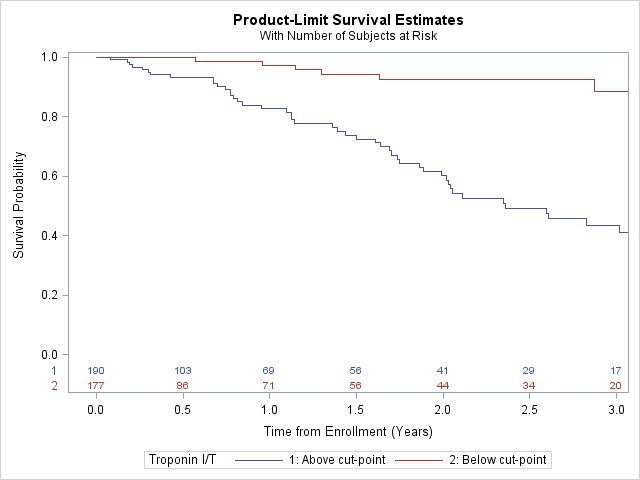

Supplement: S1 Supporting Information — (ZIP) [file pone.0173086.s001.zip › S3_Fig_KM_Troponin I-T.png]

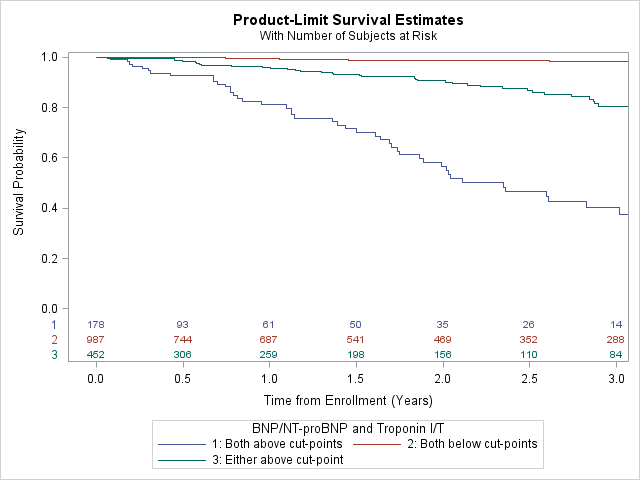

Supplement: S1 Supporting Information — (ZIP) [file pone.0173086.s001.zip › S1_Fig_KM_BNP-NT_Troponin I-T.png]

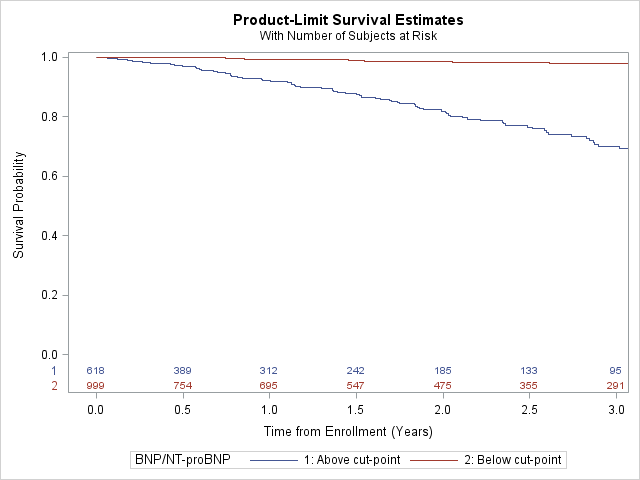

Supplement: S1 Supporting Information — (ZIP) [file pone.0173086.s001.zip › S2_Fig_KM_BNP-NTproBNP.png]
